# *Physcomitrium patens* Infection by *Colletotrichum gloeosporioides*: Understanding the Fungal–Bryophyte Interaction by Microscopy, Phenomics and RNA Sequencing

**DOI:** 10.3390/jof7080677

**Published:** 2021-08-22

**Authors:** Adriana Otero-Blanca, Yordanis Pérez-Llano, Guillermo Reboledo-Blanco, Verónica Lira-Ruan, Daniel Padilla-Chacon, Jorge Luis Folch-Mallol, María del Rayo Sánchez-Carbente, Inés Ponce De León, Ramón Alberto Batista-García

**Affiliations:** 1 Centro de Investigación en Dinámica Celular, Instituto de Investigación en Ciencias Básicas y Aplicadas, Universidad Autónoma del Estado de Morelos, Cuernavaca 62209, Mexico; adriana.ob1990@gmail.com (A.O.-B.); yordanis.perezllano@yahoo.com (Y.P.-L.); katlira@uaem.mx (V.L.-R.); 2 Departamento de Biología Molecular, Instituto de Investigaciones Biológicas Clemente Estable, Montevideo 11600, Uruguay; greboledo84@gmail.com (G.R.-B.); iponce@iibce.edu.uy (I.P.D.L.); 3 Consejo Nacional de Ciencia y Tecnología (CONACyT), Colegio de Postgraduados de México, Campus Montecillo, Texcoco 56230, Mexico; daniel.padilla@colpos.mx; 4 Centro de Investigación en Biotecnología, Universidad Autónoma del Estado de Morelos, Cuernavaca 62209, Mexico; jordi@uaem.mx (J.L.F.-M.); maria.sanchez@uaem.mx (M.d.R.S.-C.)

**Keywords:** *Physcomitrium (Physcomitrella) patens*, *Colletotrichum gloeosporioides*, fungal–bryophyte interaction, transcriptomics, phenomics

## Abstract

Anthracnose caused by the hemibiotroph fungus *Colletotrichum gloeosporioides* is a devastating plant disease with an extensive impact on plant productivity. The process of colonization and disease progression of *C. gloeosporioides* has been studied in a number of angiosperm crops. To better understand the evolution of the plant response to pathogens, the study of this complex interaction has been extended to bryophytes. The model moss *Physcomitrium patens* Hedw. B&S (former *Physcomitrella patens*) is sensitive to known bacterial and fungal phytopathogens, including *C. gloeosporioides*, which cause infection and cell death. *P. patens* responses to these microorganisms resemble that of the angiosperms. However, the molecular events during the interaction of *P. patens* and *C. gloeosporioides* have not been explored. In this work, we present a comprehensive approach using microscopy, phenomics and RNA-seq analysis to explore the defense response of *P. patens* to *C. gloeosporioides*. Microscopy analysis showed that appressoria are already formed at 24 h after inoculation (hai) and tissue colonization and cell death occur at 24 hai and is massive at 48 hai. Consequently, the phenomics analysis showed progressing browning of moss tissues and impaired photosynthesis from 24 to 48 hai. The transcriptomic analysis revealed that more than 1200 *P. patens* genes were differentially expressed in response to *Colletotrichum* infection. The analysis of differentially expressed gene function showed that the *C. gloeosporioides* infection led to a transcription reprogramming in *P. patens* that upregulated the genes related to pathogen recognition, secondary metabolism, cell wall reinforcement and regulation of gene expression. In accordance with the observed phenomics results, some photosynthesis and chloroplast-related genes were repressed, indicating that, under attack, *P. patens* changes its transcription from primary metabolism to defend itself from the pathogen.

## 1. Introduction

*Colletotrichum gloeosporioides* is a hemibiotrophic ascomycete fungus that, together with other members of the genus *Colletotrichum* (*C. graminicola, C. lindemuthianum*, *C. lupini, C. musae*), produces anthracnose in many plants [1,2]. The species complex *C. gloeosporioides* is considered the main causal agent of anthracnose, infecting more than 470 plant species [2] and causing yield losses of up to 50% in both ornamental plants (i.e., anthurium, tulip tree, geranium, lupines, etc.) [3,4,5,6] and economically relevant crops (i.e., banana, mango, avocado, apple, citrus, olive, etc.) [1]. The incidence of this fungus has been mostly studied in vascular plants, which develop dark lesions on stems, leaves, fruits and flowers during fungal infection [1].

Plants under biotic stress activate defense responses mediated by cell surface-localized pattern recognition receptors (PRRs) and intracellular disease resistance proteins, which detect pathogen-associated molecular patterns (PAMPs) and effectors, respectively, leading to pattern-triggered immunity (PTI) and effector-triggered immunity (ETI) [7,8]. Defense responses activated by PTI and ETI include mitogen-activated protein kinases (MAPKs) cascades, calcium signaling, increase in hormone production, reactive oxygen species (ROS) generation and transcriptional regulation of defense genes [7]. Recent studies revealed that immune pathways activated by cell-surface and intracellular receptors mutually potentiate to activate effective resistance against a pathogen [8]. The plant response to *C. gloeosporioides* attack has been studied in vascular plants. Dual RNA-sequencing profiles during the interaction of *C. gloeosporioides* with tomato fruits (*Solanum lycopersicum* L.) and strawberry leaves (*Fragaria ananassa* Duch) showed that this fungus induced in host plant cells a salicylic acid-dependent hypersensitive response (HR), autophagy, MAPKKK, phenylalanine and flavonoid biosynthesis, and plant cell wall modification [9,10]. In addition, the analysis of miRNA sequencing data generated from *C. gloeosporioides*-inoculated tea leaves (*Camellia sinesia* L., Kuntze) revealed that the main metabolic processes upregulated by miRNA during the fungus–plant interaction were related to receptor kinases, ROS scavenging, auxin and salicylic acid-mediated pathways [11]. Despite the plant defense response, *C. gloeosporioides* possess efficient mechanisms to colonize the host and has specific effector proteins that disrupt host response mechanisms.

Plant interactions with fungi are ubiquitous and are registered in fossil records [12,13]. The fungus–plant pathogenic relationships date back to 400 million years ago [14], when the first terrestrial plants colonized the land [15]. Bryophytes (non-vascular plants) and tracheophytes (vascular plants) are descendant of these early land plants [15,16]. Phytopathogenic microbes are capable of infecting bryophytes, which include mosses, liverworts and hornworts. The liverwort *Marchantia polymorpha* L. is colonized by fungal endophytes [17]. The infection of model mosses *Physcomitrium patens* Hedw. B&S (formerly *Physcomitrella patens*) and *Funaria hygrometrica* Hedw. by fungi (i.e., *Alternaria alternata, Botrytis cinerea, Cladosporium oxysporium, Epicoccum nigrum, Fusarium avenaceum* and *Fusarium oxysporium*)*,* oomycetes (i.e., *Pythium debaryanum* and *Pythium irregulare*) or bacteria (i.e., *Pectobacterium* spp.) have been previously documented [18,19,20,21]. *B. cinerea* is a necrotrophic ascomycete that infects *Polytrichum juniperinum* Hedw. in nature [22] and *P. patens* in laboratory conditions [23]. The defense mechanisms of *P. patens* towards pathogens were recently reviewed [24]. Several pathogens, such as bacteria, oomycetes and fungi, that attack flowering plants can also cause disease and death in *P. patens* [20,25]. *P. patens* is one of the non-vascular plant models mostly used in plant biology studies; thus, an analysis of its defense mechanisms against pathogens allows a better understanding of the evolution of plant defense mechanisms from mosses to seed plants.

Although a vast knowledge is available for angiosperm–pathogen interactions, few omics studies have analyzed the effects of widely distributed pathogen fungi (i.e., *C. gloeosporioides*) on bryophytes [24]. In this study, we investigated the interaction between this fungal pathogen and *P. patens* by microscopy, phenomics and RNA-sequencing. Studies of fungal–bryophyte interactions at the genomic scale could reveal the molecular mechanisms and ecosystem functioning effects of detrimental or endophytic fungi on metabolite production, nutrient acquisition, plant–pathogen crosstalk and resistance, and abiotic stress alleviation. It could also provide insights into the coevolution mechanisms of pathogens with bryophytes and the fungal infection strategies different from those described in vascular plants.

## 2. Results and Discussion

### 2.1. Colletotrichum gloeosporioides Successfully Infects Physcomitrium patens

**Figure 1:**
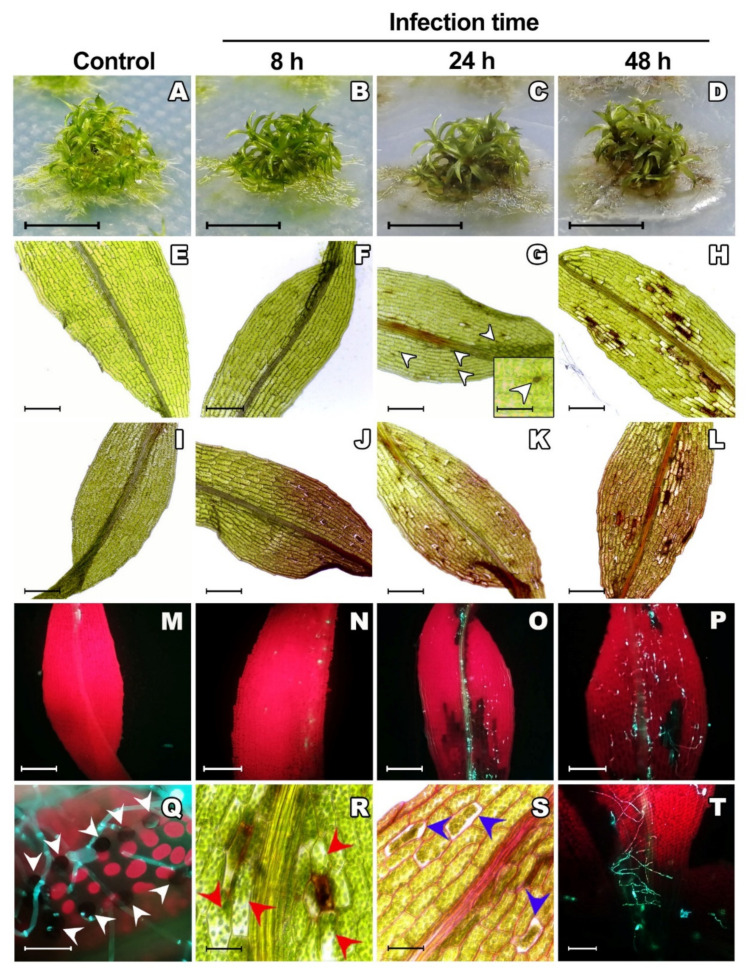
Symptom development in *P. patens* inoculated with *C. gloeosporioides*. (**A**–**D**) Images of *P. patens* colonies at different times of infection: control without inoculation, 8 h after inoculation (hai), 24 hai and 48 hai, respectively. Bars: 1 cm. (**E**–**H**) Images of unstained *P. patens* phyllids at different times of infection: control without inoculation, 8 hai, 24 hai and 48 hai, respectively. (G insert) Appressorium over a phyllid at 24 hai. White arrowheads point to appressoria; bar: 50 µm. (**I**–**L**) Images of *P. patens* phyllids stained with safranin-O at different times of infection: control without inoculation, 8 hai, 24 hai and 48 hai, respectively. (**M**–**P**) Images of *P. patens* phyllids stained with solophenyl flavine at different times of infection: control without inoculation, 8 hai, 24 hai and 48 hai, respectively. Images E-P, bars: 200 µm. (**Q**) Phyllid inoculated with *C. gloeosporioides* and stained with solophenyl flavine where appressoria formation can be seen at 24 hai (white arrowhead). (**R**) Phyllid inoculated with *C. gloeosporioides*, where the migration of chloroplasts to the site of infection can be seen (red arrowheads). (**S**) Phyllid inoculated with *C. gloeosporioides* and stained with safranin-O, where the shrinkage of the cytoplasm and staining of the plant cell walls can be seen, which reflects the reinforcement of the wall with phenolic compounds (24 hai). (**T**) Phyllid inoculated with *C. gloeosporioides* at 24 hai and stained with solophenyl flavine, where hyphae growth can be seen. Images Q-T, bars: 50 µm.

*C. gloeosporioides* infection produced visible disease symptoms in *P. patens* colonies at 24 h after inoculation (hai), turning the protonema brown colored and with a macerated appearance. Fungal colonization progressed successfully over time (24 and 48 hai), and the most prominent symptoms were observed at 48 hai (Figure 1A–D). Microscopic examination of phyllid cells infected with *C. gloeosporioides* evidenced tissue lesions at 24 and 48 hai (Figure 1E–H) and chloroplast browning (Figure 1H). Safranin-O staining demonstrated that the cell walls were reinforced in response to fungal dissemination (Figure 1I–L), while solophenyl flavine staining revealed that *C. gloeosporioides* conidia were already germinated at 8 hai and fungal colonization increased at 24 and 48 hai (Figure 1M–P). The formation of melanized appressoria on the phyllid surface and the presence of secondary hypha were evident at 24 hai (Figure 1G,Q). The migration and relocation of chloroplasts in the cells surrounding the infected cells, as well as cytoplasmic shrinkage were observed at 24 hai (Figure 1R,S). These observations confirmed the progress of *C. gloeosporioides* colonization at 24 hai, similarly as shown by solophenyl flavine staining in Figure 1T. Similar as other pathogens that infect mosses and have hemibiotrophic or necrotrophic lifestyles, *C. gloeosporioides* produces necrosis and causes maceration, eventually leading to moss death [18,25,26].

Appressorium formation during the early stage of *C. gloeosporioides* colonization is an important step in the process of fungal infection of host plants. These specialized cells generate a highly localized turgor pressure to drill plant cells [27]. This strategy has been extensively reported during *C. gloeosporioides* infection in vascular plants, such as tomato (*S. lycopersicum* L.), strawberry (*Fragaria ananassa* Duch.) and mango (*Mangifera indica* L.) [9,28,29], and in non-vascular plants, such as *P. patens*. Because *C. gloeosporioides* directly penetrates the host cell walls, their molecular composition is extensively modified and strengthened by lignification in flowering plants or accumulation of callose and phenolic compounds in non-vascular plants [30]. The positive staining with safranin-O on cells from 8 hai onward demonstrated that lignan-like compounds are accumulated in *P. patens* cells to reinforce its cell walls as a defense mechanism against fungal infection [22,23,24]. Consistent with our results, infection with other pathogens, such as the hemibiotrophic oomycete *Phytophthora capsici* and the necrotrophic fungus *B. cinerea*, provoke browning of chloroplasts and its migration towards the active infection site and cytoplasmatic shrinkage [30]. This reprograming of host cell response has been also described in liverwort and hornwort during colonization by symbiotic fungi [31,32]. Moreover, the cell death process triggered as part of the HR response, evidenced by the cytoplasmic collapse and chloroplast browning, indicates that *P. patens* attempted to control *C. gloeosporioides* infection, as was previously reported [23]. HR is triggered during association with pathogens; it plays an important role in restricting pathogen growth and also regulates the local and distant tissues defense responses [33].

All these findings are consistent with those observed in *P. patens* colonized with other pathogens, such as *A. alternata, B. cinerea, F. avenaceum, P. debaryanum* and *P. irregulare* [18,19,30]. Similar responses have been well described in vascular plants infected by fungi (i.e., *C. gloeosporioides*) [34,35,36]; however, their conservation during the evolution of bryophyte–microbe interactions remain unclear. Interestingly, chloroplast repositioning was recently observed as a stress response in the liverwort *M. polymorpha* exposed to polycyclic aromatic hydrocarbons [37]. Thus, this phenomenon, not fully understood, could play a role in bryophyte responses to biotic and abiotic stress. In summary, our results suggest that reprogramming of moss host cells induced by phytopathogenic fungi is a conserved response to microbe colonization.

### 2.2. Phenomics Characterization of the Colletotrichum gloeosporioides–Physcomitrium patens Interaction

**Figure 2:**
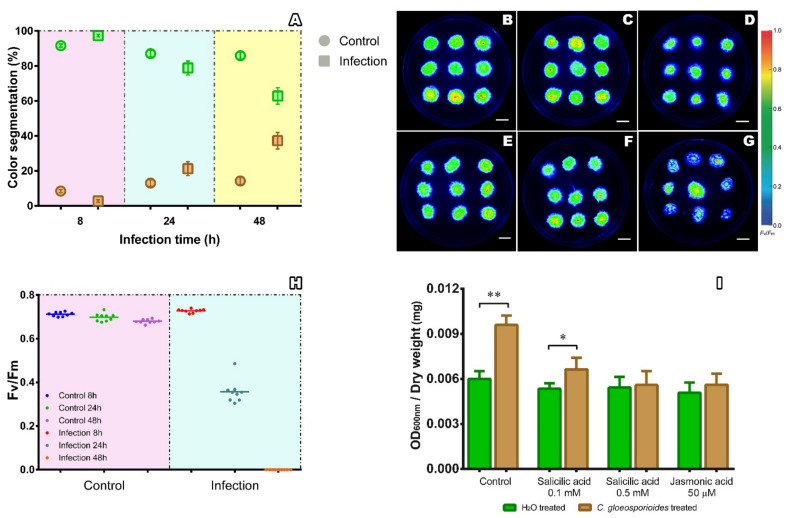
Phenomics analysis of *P. patens* during its interaction with *C. gloeosporioides*. (**A**) Effect of the interaction between *P. patens* and *C. gloeosporioides* on the green and brown color segmentation. The circles represent the controls and the squares the infected samples. The green color represents the green tissue portion, and the brown color represents the brown tissue. Each point reflects the mean (±standard deviation) of three images with nine colonies each (*n* = 27). (**B**–**G**) Fluorescent images of the moss colonies: (**B**–**D**) control without inoculation, at 8 hai, 24 hai and 48 hai, respectively; (**E**–**G**) infected colonies at 8 hai, 24 hai and 48 hai, respectively. Bars: 1 cm. (**H**) Mean of the quantum yield of Photosystem II (± standard deviation) of the *P. patens* colonies: control without inoculation (left panel) and infected samples (right panel). (**I**) Measurement of cell damage by Evans blue staining at 72 hai of colonies previously treated with hormones. Control plants had no hormone treatment before the *C. gloeosporioides* inoculation. The data are expressed as the optical density (OD) at 600 nm per milligram dry weight. The values in the graph are the means (± standard deviation) of eight independent replicas of the moss, and the asterisks on the bars indicate statistically significant differences (*p* ≤ 0.01; *n* = 8).

Phenomics technologies have evolved as an emerging tool to bridge the genotype-to-phenotype knowledge gap and serve as an image-based phenotyping technique to efficiently transform pictures into accurate phenotyping measurements [38]. We used high-throughput phenotyping to characterize the infection of *P. patens* by *C. gloeosporioides*. A color segmentation analysis was performed to quantify the *C. gloeosporioides* infection in *P. patens* (Figure 2A). The percentage of brown tissue in the colonies increased up to 40% at 48 hai, while the percentage of green tissue decreased to 60% at the end of the experiment. As expected, these effects were not observed in the controls (non-inoculated mosses) (Figure 2A). Color segmentation analysis arrays the degree of infection from green, regarded as healthy tissue, to brown, meaning necrotic tissue. The increase in brown segmentation over time correlates to the browning of chloroplasts and cell walls observed in the microscopy analysis (Figure 1H,R). Furthermore, the production of ROS in infected cells and photosynthetic imbalance could increase the brown segmentation in moss colonies. On the other hand, the synthesis of plant cell wall-degrading enzymes during necrotrophic fungal growth causes maceration of the moss tissues and finally triggers cellular death, reflecting a detrimental green segmentation.

The quantum yield of photosynthesis (*Fv/Fm*), estimated using the chlorophyll *a* fluorescence values, has been widely used as an indirect measure of stress in plants [39,40,41,42]. This parameter was evaluated in infected plants and compared to the controls at different post inoculation times (Figure 2B–H). At 8 hai, there are no significant differences in *Fv/Fm* between the controls and infected mosses (Figure 2B,E,H). However, *Fv/Fm* decreases by 45% at 24 hai (Figure 2C,F,H), and at 48 hai, the *Fv/Fm* value decreases below the equipment threshold (Figure 2G,H). These results are consistent with those obtained for the color segmentation analysis and suggest that photosynthesis is drastically affected by *C. gloeosporioides* infection. Similar results have been reported for different wheat varieties (*Triticum aestivum* L.) susceptible to the fungus *Zymoseptoria tritici*, where *Fv/Fm* decreases up to 40% in infected plants [43]. In contrast, açaí palm leaves (*Euterpe oleracea* Mart.) infected with *Colletotrichum* spp. showed a decrease of 13% in *Fv/Fm* [44].

### 2.3. Salicylic Acid and Jasmonic Acid Improved Resistance of Physcomitrium patens to Infection by Colletotrichum gloeosporioides

Phytohormones play crucial roles in flowering plant defense responses to a wide range of pathogens. The sensing of fungal colonization initiates complex signaling pathways in infected vascular plants and triggers hormone synthesis, these being ethylene, salicylic acid, jasmonic acid, abscisic acid and auxin, known to play major roles in mediating plant defense responses against fungal infections [45,46,47]. Similarly, several phytohormones, such as salicylic acid, abscisic acid and auxin, participate in the defense response of mosses against *B. cinerea*, *Pectobacterium wasabiae*, *P. irregulare* and *P. debaryanum* [19,30,48,49]. Here, we evaluated the role of salicylic acid and jasmonic acid in the progress of *C. gloeosporioides* infection in *P. patens*.

Moss colonies grown on media supplemented with salicylic acid and jasmonic acid showed less infection levels, reflected in lower cell damage (Figure 2I). This result indicates that the incorporation of salicylic acid and jasmonic acid had a positive effect on *P. patens*’ responses against *C. gloeosporioides* infection. In the moss, the hormone levels vary during biotic stress [30,50]. For example, *P. patens* increases the salicylic acid levels in response to *B. cinerea* infection. Salicylic acid also induces the expression of the phenylalanine ammonia lyase gene (*pal*), reflecting the role of salicylic acid in moss defense responses to *B. cinerea* [30]. Moreover, *P. patens* colonies exposed to salicylic acid show higher resistance to the infection by *P. wasabiae* [48]. Other plant pathogens, such as the oomycete *P. irregulare*, also turns on a salicylic acid-like response in the moss *Amblystegium serpens* Hedw. [49]. Contrarily, salicylic acid treatments of the liverwort *M. polymorpha* promotes the necrotrophic infection by the fungus *Irpex lacteus* [51]. This is because salicylic acid acts antagonistically to 12-oxophytodienoic acid (OPDA, a jasmonic acid precursor), which is the main mediator of *M. polymorpha* immunity against *I. lacteus* [52]. Interestingly, *P. patens* also increases its resistance to infection by addition of jasmonic acid at the medium culture (Figure 2I). Although *P. patens* does not synthetize jasmonic acid, the moss expresses functionally active genes for all jasmonic acid signaling components [53]. Jasmonates and OPDA also induce the expression of *pal* in moss plants [54]. The major role of hormones in biotic stress caused by pathogens has been documented extensively in vascular plants, such as for sorghum (*Sorghum bicolor* L.), potato (*Solanum tuberosum* L.) and wheat (*T. aestivum* L.), where the accumulation of salicylic acid resulted in greater expression of plant defense genes [55,56,57]. In vascular plants, salicylic acid mainly activates the defense responses against biotrophic pathogens, while jasmonic acid and ethylene induce defense responses against necrotrophic pathogens [58].

### 2.4. Transcriptomic Analysis in Physcomitrium patens

**Figure 3:**
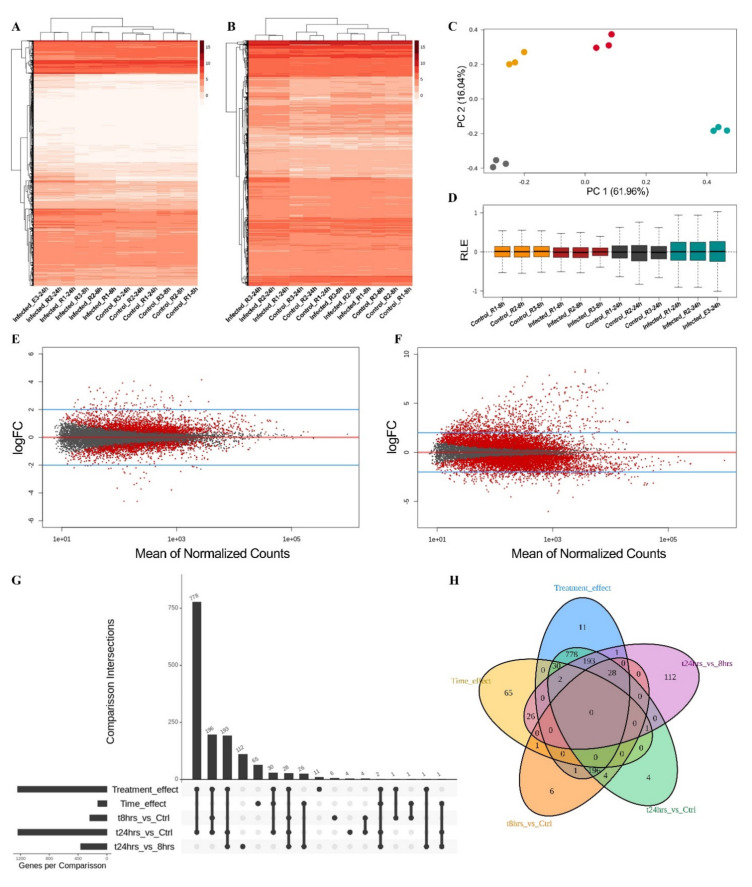
Differential gene expression analysis of *P. patens* under infection with *C. gloeosporioides*. (**A**,**B**) Effect of filtering out the genes with low read counts on the gene expression profile and discrimination between experimental groups by unsupervised clustering. (**C**) Principal component analysis (PCA) after upper-quartile normalization, showing the clustering of the treatment groups. (**D**) Relative log expression (RLE) plot after upper-quartile normalization, showing the read distribution across samples. (**E**,**F**) Mean difference plots of differentially expressed genes under a time effect and treatment effect, respectively. (**G**) Upset plot showing the sets of differentially expressed genes from the different comparisons between samples, including the quantitative analysis of the aggregate intersections between comparisons. The vertical bars show the number of intersecting genes between comparisons, denoted by the connected black circles below the histogram. The horizontal bars show the gene set size. (**H**) Venn diagram, showing the overlap among the differentially expressed genes of different comparisons.

A total of 93.7% of moss reads were successfully mapped to the genomes of *P. patens*, including the nuclear, chloroplast and mitochondrial genomes. Figure 3A,B show the effect of filtering out the genes with low read counts on the gene expression profile and discrimination between experimental groups by unsupervised clustering. Principal component analysis showed the clustering of the treatment groups after upper-quartile normalization (Figure 3C). The effect of the normalization process was also observed in the relative logarithmic expression (RLE) values, which showed uniform distribution of reads across samples (Figure 3D).

For differential gene expression analysis, the time effect (24 hai vs. 8 hai) and the treatment effect (infected moss vs. non-infected moss) were simultaneously evaluated (Figure 3E,F). A statistical analysis was also performed to establish the set of differentially expressed genes from the different comparisons between samples (Figure 3G). This analysis included the following comparisons: (i) treatment effect (differentially expressed genes to the infection by *C. gloeosporioides* vs. the control (non-infected moss)); (ii) time effect (differentially expressed genes over time); (iii) 8 hai vs. non-infected mosses; (iv) 24 hai vs. non-infected mosses; and (v) 24 hai vs. 8 hai (Figure 3G,H). The multivariate analysis evidenced that the comparisons (i) of treatment effect and (iv) 24 hai vs. non-infected mosses showed the higher amount of differentially expressed genes, 1240 and 1236, respectively. All differentially expressed genes found in the later comparison were also identified as differentially expressed genes in the treatment effect analysis. These results indicated that the moss infection by *C. gloeosporioides* (treatment–infection–effect) denoted the bigger transcriptional reprogramming in *P. patens*.

Interestingly, the analysis (v) 24 hai vs. 8 hai was not informative about the moss defense response during fungal infection since 363 genes were differentially expressed and 112 of them were exclusive of this comparison (Figure 3G,H), which did not reflect any enriched biological process. The analysis revealed 236 differentially expressed genes (all upregulated) at 8 hai, while 836 upregulated and 400 downregulated genes were found at 24 hai. Surprisingly, only 4 and 6 differentially expressed genes were exclusively identified in the comparisons (iv) 24 hai vs. non-infected mosses and (iii) 8 hai vs. non-infected mosses, respectively (Figure 3G,H), indicating that the results of these comparisons are redundant with that of the treatment effect. In addition, the time effect analysis was also a non-informative comparison regarding the defense response of the plant. Sixty-five differentially expressed genes were exclusive for the time effect (Figure 3H). These genes are involved in the circadian cycle of the plant since the 8 hai samples were collected closely to the photoperiod change. As our results show, the infection effect was the category with a more comprehensive influence on transcriptional reprogramming of *P. patens* by *C. gloeosporioides* infection. The moss upregulated and downregulated 837 and 403 genes, respectively (1240 genes in total) (Tables S1 and S2). Eleven differentially expressed genes were exclusively found in this comparison (Figure 3G,H). In the following, our attention was focused on differentially expressed genes due to the treatment–infection–effect.

**Figure 4:**
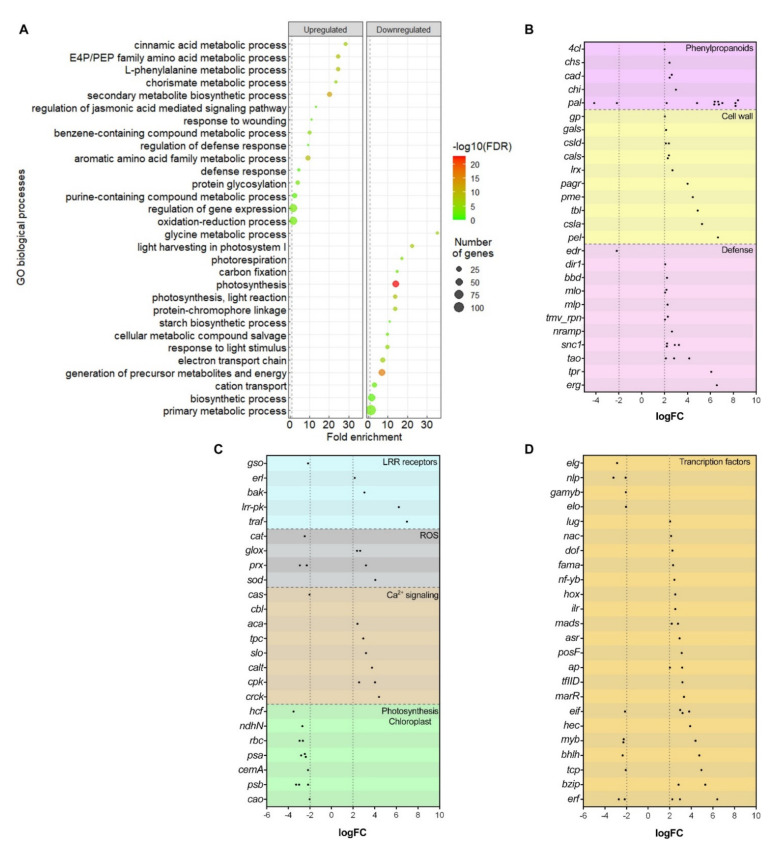
Enriched biological processes and their genes. (**A**) Gene ontology enrichment analysis of differentially expressed genes in *P. patens*. The most significant biological processes are represented and ordered according to their enrichment. The dot size indicates the number of differentially expressed genes associated with the process, function or component and the dot color indicates the significance of the enrichment (−log_10_ FDR). The vertical grey dashed line represents a fold enrichment of 1. E4P: erythrose 4-phosphate; PEP: Phosphoenolpyruvate. (**B**) Differential expression of genes corresponding to phenylpropanoids, cell wall reinforcement and defense biological processes. *4cll*: 4-Coumarate-CoA ligase*; chs*: Chalcone synthase*; cad*: Cinnamyl alcohol dehydrogenase*; chi*: Chalcone-flavonone isomerase*; pal*: Phenylalanine ammonia-lyase*; gp*: Vegetative cell wall protein gp1*; gals*: Galactan beta-1,4-galactosyltransferase*; csld*: Cellulose synthase*; cals*: Callose synthase*; lrx*: Leucine-rich repeat extensin-like protein*; pagr*: Protein pectic arabinogalactan synthesis-related*; pme*: Pectinesterase*; tbl*: Protein trichome birefringence*; csla*: beta-Mannosyltransferase*; pel:* Pectate lyase*; edr*: Protein Enhanced Disease Resistance*; dir1*: Putative lipid-transfer protein DIR1*; bbd*: bifunctional nuclease 1-like*; mlo*: MLO-like protein*; mlp*: MLP-like protein*; tmv_rpn*: TMV resistance protein N-like*; nramp*: Nramp-domain-containing protein*; snc1*: Protein suppressor of NPR1-1, constitutive 1*; tao*: Disease resistance protein TAO1*; tpr*: Topless-related protein*; erg*: Elicitor-responsive protein*; gso*: LRR receptor-like ser/thr-protein kinase GSO1*; erl*: LRR receptor-like ser/thr-protein kinase ERL1. (**C**) Differential expression of genes corresponding to receptors, ROS, Ca^2+^ signaling and photosynthesis/chloroplast biological processes. *bak*: LRR receptor kinase BAK1*; lrr-pk*: Probable leucine-rich repeat receptor-like protein kinase*; traf*: TNF receptor-associated factor homolog 1a*; cat*: Catalase isozyme 2*; glox*: Aldehyde oxidase GLOX-like*; prx*: Peroxidases N1-like, A2-like and Thioredoxin peroxidase 1*; sod*: Superoxide dismutase [Cu-Zn]*; cas*: Calcium sensing receptor*; cbl*: Calcineurin B-like protein*; aca*: Calcium-transporting ATPase*; tpc*: Two pore calcium channel protein*; slo*: Calcium-activated potassium channel slowpoke-like*; calt*: Caltractin*; cpk*: Calcium-dependent protein kinase*; crck*: Calmodulin-binding receptor-like cytoplasmic kinase*; hcf*: High chlorophyll fluorescence phenotype 173*; ndhN*: NAD(P)H-quinone oxidoreductase subunit N*; rbc*: Ribulose-1,5-Bisphosphate Carboxylase/Oxygenase*; psa*: Photosystem I–apoprotein*; cemA*: Chloroplast envelope membrane protein*; psb*: Photosystem II protein*; cao*: Chlorophyllide a oxygenase. (**D**) Differential expression of genes corresponding to Transcription factors. *elg*: Transcription factor EGL1*; nlp*: Protein NLP1*; gamyb*: Transcription factor GAMYB*; elo:* Transcription elongation factor B polypeptide 3*; lug*: Transcriptional corepressor LEUNIG*; nac*: NAC domain-containing protein*; dof*: DOF zinc finger protein*; fama*: Transcription factor FAMA*; nf-yb*: Nuclear transcription factor Y subunit B-3*; hox*: Homeobox-leucine zipper protein HOX20*; ilr:* Transcription factor ILR3*; mads*: Putative MADS-domain transcription factor*; asr*: Trihelix transcription factor ASR3*; posF*: Probable transcription factor PosF21*; ap*: AP-1 complex*; tfIID*: Transcription initiation factor TFIID*; marR*: Transcriptional regulator, MarR family*; eif*: Eukaryotic translation initiation factor*; hec*: Transcription factor HEC2*; myb*: Transcription factor MYB21, PHL7 and MYB3R-5*; bhlh*: Transcription factor bHLH66 and bHLH49*; tcp*: Transcription factor TCP15*; bzip*: Basic leucine zipper transcription factor 60 and 61*; erf*: Ethylene-Responsive Transcription Factor RAP 2.11, ERF039, ERF084 and ERF043.

Gene ontology (GO) enrichment analysis showed that the most enriched metabolic processes (fold enrichment > 25) were the cinnamic acid pathway, erythrose 4-phosphate/phosphoenolpyruvate (E4P/PEP) family amino acid pathway, L-phenylalanine and chorismate metabolic process (Figure 4A). All these processes are related with the shikimate and phenylpropanoids pathways, from which salicylic acid and phenolic compounds are synthetized [59]. Secondary metabolite biosynthetic processes were also significantly enriched (fold enrichment > 20), reflecting that moss secondary metabolism plays a pivotal role in *P. patens* defense response to fungal infection [24]. Defense response metabolism, including response to wounding, and the regulation of these processes were also upregulated (Figure 4A). Oxidation–reduction processes were also enriched, probably as a moss response to the production of ROS during fungal infection [50]. Finally, the regulation of gene expression was enriched (Figure 4A), reflecting the extensive transcriptional reprogramming that occurred in *P. patens* by *C. gloeosporioides* colonization. Transcriptional reprogramming has been previously reported for many plants during fungal infections [60,61,62].

Among the biological processes, several metabolic pathways related to photosynthesis, chloroplast functioning and starch biosynthesis were downregulated (Figure 4A). The primary metabolism was also turned off with more than 75 genes downregulated. Interestingly, glycine metabolic process was dramatically affected during fungal infection (Figure 4A). High glycine contents in plants are pivotal to produce plant glycine-rich domain proteins (GRPs), which are involved in plant stress alleviation to diverse multiple abiotic stressors, such as thermic and hydric tolerance [63]. Some of these GRPs also participate directly in blocking plant viral infection [64], while others have showed anti-fungal and anti-bacterial activity against *B. cinerea, F. oxysporium, Mycosphaerella oxysporium* and *Pseudomonas syringae* [65,66].

#### 2.4.1. Phenylpropanoids Pathway and Cell Wall Reinforcement

Phenylpropanoid biosynthesis plays an important role in plants since many secondary metabolites involved in signaling and plant defense are obtained from this biochemical pathway [67]. For example, salicylic acid and polyphenol compounds (i.e., flavonoids, phenylpropanoids, lignin and lignin-like metabolites), which participate by activating different mechanisms during the reinforcement of the plant cell wall, are obtained from phenylpropanoid metabolism [68]. Genes encoding five of the main enzymes (i.e., phenylalanine ammonium lyase (PAL), chalcone synthase (CHS), chalcone-flavanone isomerase (CHI), cinnamyl alcohol dehydrogenase (CAD) and 4-coumarate CoA ligase (4CL)) involved in the biosynthesis of polyphenol compounds were overexpressed in *P. patens* during *C. gloeosporioides* interaction. While *chs, chi, cad* and *4cl* increased 4–8-fold their expression, *pal* genes were strongly upregulated. Interestingly, 10 different *pal* genes were upregulated, some of them until 330-fold (Table S3), suggesting that the expression of this enzyme-encoding genes (the first and committed step in the phenylpropanoid pathway) could be relevant during the moss–fungus interaction. *pal* genes are found extensively in plants, including mosses, and they are involved in the response to different abiotic and biotic stimuli, such as pathogenic infection [69,70,71]. It also has been proposed that high levels of PAL concentrations increase the metabolic flux through the pathway [72].

The presence of lignin in the *P. patens* cell wall has not been reported. However, the presence of lignans (lignin-like compounds) has been described as a cell wall fortification response in this moss [73]. Lignans constitute abundant classes of phenylpropanoids synthetized in many plants and they are related to plant defense systems. Structurally related lignan production is induced during plant–pathogen interaction [74,75], and we found the *pal* and *chs* genes, encoding the enzymes involved in the early steps of lignan biosynthesis [67], were upregulated during the *P. patens* fungal infection (Figure 4B). All lignan biosynthetic pathways initiate with deamination of phenylalanine by PAL [76]. This correlates with the transcriptomic findings obtained in this study. As discussed early, Figure 1J–L shows the presence of phenolic compounds associated with the cell wall of mosses infected by *C. gloeosporioides*. This observation also correlates with our transcriptomic findings related to the phenylpropanoid pathway.

Several genes related to cell wall biogenesis and modification were also overexpressed, including those encoding for callose synthase (CALS), cellulose synthase (CSLD), protein pectic arabinogalactan synthesis-related (PAGR) and β-mannosyl transferase (CSLA), among others (Figure 4B). The cell walls of *P. patens* are mainly composed of cellulose, hemicellulose (mainly mannans), arabinogalactan-proteins and, to a lesser extent, pectin (mainly homogalacturonan) [77]. Therefore, genes coding for synthesis of these components are overexpressed to strengthen the cell wall in response to pathogen attack. For example, *cals* genes are involved in the callose synthesis, a polymer of β-1,3-D-glucan. Callose accumulates in plants cell walls in responses to different stresses, including pathogen attack [78]. Local deposition of callose (named papillae) in the cell walls of *P. patens* in the presence of pathogens has been previously reported [50,79]. Papillae provide mechanical and chemical barriers for blocking pathogen penetration or delay the infection process [80].

Other genes that were overexpressed are those coding trichome birefringent protein (TBL) and galactan enzyme β-1,4-galactosyltransferase (GALS). TBL is a protein located in the Golgi complex and is responsible for acetylating polymers present in the cell wall. This protein has been related to resistance to rice blight disease in *Oryza sativa* L. [81]. On the other hand, *gals* genes participate in the biosynthesis of β-1,4-galactan, which is one of the main side chains of galacturonans, the abundant polysaccharides in plant cell walls that are the major component of pectin [82]. The high levels of transcriptional expression of the aforementioned genes suggest that the reinforcement of the cell wall is one of the main defense mechanisms deployed by *P. patens* during *C. gloeosporioides* attack.

#### 2.4.2. Defense-Related Genes Expression

Plants respond to pathogen infection by reprogramming the transcription of defense-related genes involved in signaling, resistance to diseases and tolerance to different stressors [83]. We found differentially expressed defense-related genes in the moss infected by *C. gloeosporioides* (Figure 4B). Two genes, *erg* and *tpr*, coding for elicitor responsive protein (ERG) and transcriptional corepressor (TPR1) (Topless Related 1), respectively, were the most upregulated genes (logFC > 6) related to defense response in *P. patens* during infection by *C. gloeosporioides* (Figure 4B). ERG is a soluble cytosolic protein with a small C2 Ca^2+^-binding domain, which participates in the Ca^2+^ signaling pathways in response to fungal effectors [84]. The transcriptional levels of the *erg* genes were dramatically upregulated in *O. sativa* L. treated with fungal elicitor obtained from mycelia of *Magnaporthe grisea* [85]. On the other hand, *tpr* genes have been shown to be involved in the transcriptional reprogramming of multiple defense genes in plants [86]. During this molecular reprogramming, high transcriptional levels of *snc* genes are needed since TPR1 is a SNC1-asociated protein. TPR1 is absolutely required for SNC1-mediated immune responses, resulting in the transcriptional co-regulation that represses the transcription of defense no death genes (i.e., *dnd1* and *dnd2*) [86]. We also found that *snc* genes were upregulated in *P. patens* infected by *C. gloeosporioides* (Figure 4B). SNC1, a protein belonging to the family of TIR-NB-LRR receptors (Toll-like Interleukin 1 Receptor–Nucleotide Binding–Leucine Rich Repeats), is a plant R protein that participates in triggering salicylic acid-dependent defense mechanisms [87]. SNC1-mediated resistance genes are also required to implement a successful ETI to induce resistance during plant–pathogen interactions [88]. In particular, salicylic acid signaling is recognized as one of the main induced disease resistance responses in plants, including mosses (i.e., *P. patens*) [89]. Salicylic acid mediates both local and systemic response to pathogen attack, resulting in a great regulatory potential to turn on the expression of multiple pathogenesis-related genes and the synthesis of defense compounds with antimicrobial activity involved in both local and systemic acquired resistance [90]. Salicylic acid signaling significantly increases the plant fitness to respond to a variety of pathogens [91].

Genes coding for TAO proteins (target of AvrB operation) were also upregulated in *P. patens* infected by *C. gloeosporioides* (Figure 4B). TAO1, a TIR-NB-LRR disease resistance protein reported as the receptor for the AvrB effector protein of *P. syringae*, has been shown to mediate an effective HR in *Arabidopsis* H&H, resulting in the restriction of pathogen growth [92]. The activation of *tao* genes has also been associated with the expression of genes involved in ethylene biosynthesis [93]. It also has been shown that *tao* genes were transcriptionally upregulated by ethylene precursor 1-aminocyclopropane (ACC) and auxin 1-napthaleneacetic acid (NAA) but downregulated by ethylene and auxin inhibitors [93]. These results indicate that *tao* expression is associated with ethylene and auxin levels in *Arabidopsis* H&H, suggesting that TAO1 could be involved in hormone-dependent defense mechanisms. *nramp* genes participate in the ethylene-defense response and were also upregulated (Figure 4B). NRAMP domain-containing proteins have been described as an evolutionary conserved ethylene signaling components and *nramp* genes are present in genomes of aquatic ancestors of land plants [94]. On the other hand, we found overexpression of the *mlp* gene, which has been recently related to increased drought tolerance and transcriptional level of abscisic acid synthetic genes in tobacco. The overexpression of *mlp* has also been correlated to reduced ROS accumulation and membrane damage during drought stress [95]. In addition, the *edr* gene, which encodes an enhanced disease response protein, was downregulated in the *P. patens* transcriptome (logFC = −2.17). EDR2 is a negative regulator of the salicylic acid-dependent response [96]. These transcriptional profiles together with the fact that salicylic acid improved the resistance of *P. patens* to infection by *C. gloeosporioides* show that hormone-dependent responses play a pivotal role in moss immunity to overcome pathogens.

Other upregulated gene was *dir1* (Figure 4B), which encodes a non-specific lipid transfer protein that plays a key role in systemic acquired resistance [97]. DIR1 is a key mobile component of the systemic acquired resistance (SAR) response and is involved in induction and SAR long distance signaling [98]. DIR1 is translocated through long distances during SAR, from the site of infection to healthy areas of the plant where it induces defense responses [98]. A large-scale transcriptional study showed that *dir1* genes were not differentially expressed in moss tissues during *B. cinerea* necrotrophic colonization [24].

Moreover, the *tmv-rpn, bbd* and *mlo* genes were upregulated during fungal infection (Figure 4B). *tmv-rpn* genes encode proteins that confer resistance to virus and fungal colonization, respectively, by inducing an HR at the infected site [99], while bifunctional nucleases (BBD) have dual RNase and DNase activity [100]. While TMV resistance N-like proteins probably inhibit the spread of fungal infection in the moss, BBD is involved in the formation of abscisic acid-derived callose deposition in the cell wall by necrotrophic fungal growth [100]. Finally, the *mlo* genes are present in the genomes of all vascular plant species and confer resistance to almost all isolates of fungi causing barley powdery mildew [101]. The transcriptional activation of *mlo* genes could suggest that *mlo*-based resistance is a defense mechanism occurring in *P. patens* during infection by *C. gloeosporioides*. Interestingly, *mlo* genes were also induced during *P. patens–B. cinerea* interaction [24]. MLO activity is enhanced by calcium-dependent protein kinases (CPK) [101]. *cpk* genes were also overexpressed in *C. gloeosporioides*-infected *P. patens* tissues (Figure 4C). This fact supports the hypothesis that *mlo*-mediated resistance could have a positive role in moss immunity. Further investigations should be conducted to identify the role played by MLO proteins in moss defense responses against pathogens.

#### 2.4.3. Leucine-Rich Repeat Receptors

Membrane receptor protein kinases containing leucine-rich repeat domains (LRRs) constitute the first line of defense in plants and are key regulators of plant growth and development [102]. LRRs recognize certain ligands and activate pathogen resistance signaling pathways that lead to plant defense responses [102]. Eleven differentially expressed genes encoding LRRs were found in the *P. patens* transcriptome (Tables S1 and S3), from which five are known to be involved in vascular plant immunity (Figure 4C). The *traf* gene showed the highest transcriptional activation (logFC ≈ 7) among genes coding LRRs. TRAF1A is a TNF receptor-associated factor that regulates the plant immune response, mediating the renewal of the SNC1 nuclear-binding domain. The activation of *traf* prevents the hyperaccumulation of SNC1 that could cause autoimmune plant phenotypes [103].

A gene encoding BAK1, a dual specificity kinase acting on both serine/threonine- and tyrosine-containing substrates, was also upregulated. BAK1 forms a protein complex with BIR1 (brassinosteroid insensitive receptor) and FLS2 (flagellin sensing 2 receptor) [104]. In this moss, BAK1 is probably associated only to BIR1 since *P. patens* does not have *fls2* genes [50]. BAK1 is a membrane protein with extracellular LRR and intracellular kinase domains and acts as a coreceptor for other membrane proteins that also contain LRR domains [104]. In *S. lycopersicum* L., BAK1 has been reported as an essential coreceptor for immune response activation mediated by receptor-like protein Ve1 and Cf receptors, which recognize pathogen effectors [105]. In *A. thaliana* H&H, the function of some LRR receptors (i.e., RPL30) was also compromised in *bak1* mutant plants, corroborating the role of *bak1* genes to install defense response in plants [106]. The *lrrc-pk* and *elr* genes, encoding serine/threonine kinases containing LRR domains, were also differentially expressed (logFC > 2). These receptors participate in the recognition of PAMPs in the plant–microbe interaction [107]. Particularly, ELR1 is associated directly with BAK1 in *A. thaliana* H&H and *S. lycopersicum* L. and it has been shown that ELR1 expression reduces the growth of *P. infestans* in infected plants [108]. The role of these LRR in mosses has not been elucidated yet.

Extensins are another important class of plant cell wall proteins with LRR domains. In the *P. patens* transcriptome, we identified an overexpressed gene (*lrx*) that encodes a moss extensin. These proteins play a key role in plant development as regulators of cell wall expansion, or as chains that connect the cell wall with the plasma membrane [109]. However, it is probable that, in this case, LRX is involved in remodeling and strengthening of the moss cell wall. Finally, the *gso* gene, involved in plant development [110], was negatively regulated (logFC = −2.17) in the moss transcriptome (Figure 4C). It is probable that, due to the biotic stress imposed on *P. patens*, the processes of development and plant growth were negatively regulated.

#### 2.4.4. Reactive Oxygen Species

ROS play a central role in plant signaling and immune response, since they have a direct antimicrobial effect and, most importantly, act as local and systemic signaling molecules [111]. In the *P. patens* transcriptome, several genes related to ROS metabolism were differentially expressed, including *glox*, *prx* and *sod* (Figure 4C). Aldehyde oxidase (GLOX), localized in the moss cell wall, catalyzes the oxidation of aldehydes and produces H_2_O_2_ [112]. Likewise, the *prx* genes coding peroxidases A2, N1 and thioredoxin peroxidase are also differentially expressed. Peroxidase N1, a class III peroxidase, was upregulated (logFC > 3). This enzyme is involved in regulating the H_2_O_2_ levels to alleviate damage by oxidative stress [113]. Interestingly peroxidase A2 and thioredoxin peroxidase were downregulated (Figure 4C). Since excessive concentrations of ROS are toxic and can lead to oxidative stress, the balance between ROS production and clearance needs to be delicately regulated to maintain cell homeostasis [114]. In this case, the *sod* genes are relevant, and we found one overexpressed *sod* gene (Figure 4C). Superoxide dismutase (SOD) oxidizes superoxide radicals, producing H_2_O_2_. The detoxification process of ROS includes a reaction that catalyzes the conversion of H_2_O_2_ into H_2_O and O_2_ by catalases or other peroxidases [115]. However, catalases were downregulated in the transcriptomic analysis. This could lead to H_2_O_2_ accumulation in cells with the consequent oxidative stress, which could trigger HR and conclude in programmed cell death [116]. In fact, HR is a successful strategy against biotrophic pathogens, since it confines the microorganism to the infection site [117]. However, contradictorily, when plants recognize necrotrophic or hemibiotrophic pathogens, such as *C. gloeosporioides*, HR only contributes to faster spreading of the pathogen. Probably the transcriptional regulation of some genes involved in ROS alleviation in *P. patens* could be correlated with overexpression of *mlp*, which increased the expression of ROS-related genes (i.e., SOD) in tobacco during abiotic stress [95].

#### 2.4.5. Other Differentially Expressed Genes

To ensure functions such as cell wall strengthening and long distance signaling, it is necessary to transport molecules from the production site in the cell to the cell surface. In many cases, this movement occurs through vesicle-mediated transport [118]. Ten genes coding for proteins involved in vesicular trafficking were overexpressed in the *P. patens* transcriptome (Table S1). The cytoskeleton is essential for vesicular movement [119], and there were 13 genes differentially expressed in *P. patens* related to cytoskeleton dynamics.

Cell signaling pathways are vital in defense responses in plants. Among them, Ca^2+^ signaling plays a fundamental role in the plant immune responses [120]. Several calcium sensors have been characterized to transmit and/or decode phytopathogen inducing Ca^2+^ signals [121]. Among the differentially expressed genes in *P. patens* during its interaction with *C. gloeosporioides*, eight genes related to Ca^2+^ signaling were identified (Figure 4C). The Ca^2+^-dependent protein kinase gene (*cpk*), for example, was overexpressed. As previously discussed, this kinase has been related with salicylic acid-mediated resistance, but also it has been associated to differential expression of defense genes and ROS synthesis [122]. A sequence encoding a Ca^2+^-dependent potassium channel was also upregulated (logFC = 3.18). Potassium is an important molecule for the defense response since it regulates ROS levels and increases the phenols concentration and synthesis of defense compounds [123].

Transcription factors are known to play essential roles in regulation of a variety of genes involved in defense responses of plant cells. They regulate transcriptional networks to activate or suppress gene expression in response to internal and external stimuli [124]. In this case, 39 sequences coding for transcription factors were differentially expressed (Figure 4D), some of them belonging to the ethylene responsive transcription factors family (ERF). In our transcriptome, *erf-rap2.11* was overexpressed (Table S3). This transcription factor binds to the GCC-box pathogenesis-related promoter element [125], which modulates the expression of many pathogenesis–related genes. The MYB transcription factor was also upregulated in *P. patens*, and this type of transcription factor activates the expression of *pal* genes [126]. Several zinc finger motif transcription factors were also found upregulated. This family of transcription factors are related to various cellular processes, including a defense response [127].

No differentially expressed genes encoding WRKY-like transcription factors were found. These transcription factors belong to one of the largest families of transcriptional regulators in plants and are key regulators of plant immunity [128]. The upregulation of WRKY-like transcription factors has been reported in *S. lycopersicum* L. and *F. ananassa* Duch. infected by *C. gloeosporioides* and *C. fructicola*, respectively, where they have been defined as key elements regulating the defense response [9,10]. However, the overexpression of WRKY-like transcription factors in *F. ananassa* Duch. during the interaction with *C. gloeosporioides* occurred at 72 hai (long-time infection). This is consistent with the result obtained in this work since WRKY-like transcription factors were not upregulated in *P. patens* at the early times (8 and 24 hai). In contrast, the transcriptome of *P. patens* infection with *B. cinerea* showed upregulation of several WRKY-like transcription factors from 8 hai [24], which could be related to a faster colonization process of *B. cinerea* compared to *C. gloeosporioides.*

Finally, we found that every differentially expressed gene related to photosynthesis or chloroplasts was downregulated (Figure 4C). Importantly, the *rbc* genes that encode enzyme ribulose bisphosphate carboxylase (RuBisCO), which fixes CO_2_ in the first step of the Calvin Cycle, were downregulated [129]. Genes encoding Photosystem I apoproteins (*psa*), Photosystems II complex proteins (*psb, hcf*), and other genes relevant for chloroplast metabolism, such as those related to the electron transfer across the thylakoid membrane (*ndhN*), chlorophyll biosynthesis (*cao*) and chloroplast envelope membrane proteins (*cemA*), were also downregulated during the moss–fungus interaction. Chloroplasts are an important source of ROS and salicylic acid during the defense response [130]. The negative regulation of so many elements could lead to a malfunction of the organelle, thus resulting in the drop of the quantum yield of Photosystem II previously discussed.

As evidenced, plant defense responses against pathogens are mediated by the activation and repression of a large set of mRNAs. However, nothing is known about the role of non-coding RNAs (i.e., lncRNAs and miRNAs) in the bryophyte–fungus interaction. For example, plant miRNAs can regulate the expression of pathogenesis-related genes during fungal infection [131,132]. The investigation of miRNAs could be a new perspective for further studies to understand bryophyte–fungus interactions since miRNAs are a common component in the cross-kingdom communication between plants and fungi.

## 3. Materials and Methods

### 3.1. Colletotrichum gloeosporioides and Physcomitrium patens Co-Inoculation Assay

*C. gloeosporioides* isolated from orange fruits [25] was grown on Potato-Dextrose Agar (PDA) (Oxoid) at 22 °C, with a photoperiod of 16 h light/8 h dark. Conidia were recovered from 15-day-old cultures and a 5 × 10^5^ conidia/mL spore suspension was prepared using milliQ water. Three milliliters of this solution were sprayed over 21-days-old moss *P. patens* (Gransden wild type) colonies grown on Hoagland solid media (1 mM MgSO_4_, 1.8 mM KH_2_PO_4_, 10 mM KNO_3_, 45 µM FeSO_4_, 1 mM CaCl_2_, 4.5 mM, ammonium tartrate, 1% agar, pH 6.5) at 22 °C and with a 16 h light/8 h dark regime under 60–80 μmol photons m^−2^ s^−1^ white light. Control (non-inoculated) moss cultures were implemented and sprayed with sterile milliQ water.

For microscopic, phenomic and transcriptomic studies, the fungal–moss co-cultures were analyzed at 8 h and 24 hai, time points corresponding to the early (spore germination) and active (moss cell infection) stage of *C. gloeosporioides* colonization, respectively. In addition, 48-h-old (late stage of infection) co-cultures were included for microscopic and phenomic characterization. All experiments were performed in triplicate using three independent biological replicates.

### 3.2. Moss and Fungal Staining

Moss samples were stained with 0.01% safranin O solution for 15 min, rinsed twice with distilled water and observed on a Leica DM500 microscope, (Leica Microsystems, Wetzlar, Germany). To demonstrate *C. gloeosporioides* infection, moss samples were stained with 0.1% solophenyl flavine 7GFE 500 [30] and observed in a fluorescence Zeiss Axiovert 200 M microscope (Oberkochen, Germany). A photographic record was registered in all cases.

### 3.3. Phenomics Analysis

*P. patens*’ colony images were acquired using a semi-automatized imaging acquisition system Scanalyzer^PL^ (LemnaTec GmbH, Aachem, Germany) equipped with a high-resolution (1628 × 1236 pixels) camera (Baster AG, Ahresburg Germany). Moss images were obtained using RGB-LED within the visible spectrum (400–700 nm). The top view of each moss colony, as well as 360° lateral views, were photographed. Tissue pixel area was determined to estimate the digital biomass and senescence was evaluated using the color segmentation profile, which was calculated using the LemnaGrid software (LemnaTec GmbH, Aachem, Germany). This computer algorithm segments and classifies the total canopy into values that correspond to the percentage of colors, taking the optimal condition criterion (green), senescent (yellow) and necrotic (brown).

Chlorophyll fluorescence images were captured from moss colonies using a PSI Open FluorCam FC 800-O system (PSI, Brno, Czech Republic). Previously, *P. patens*’ colonies were adapted to dark conditions for 20 min. Fluorescence maps of the moss colonies were obtained by stimulating the maximum quantum yield of Photosystem II, expressed as *Fv/Fm* (*Fv*: variable fluorescence; *Fm*: maximum fluorescence). The PSI Open FluorCam system is equipped with four LED panels (actinic lights -618 nm-, intensity interval from 200 to 400) divided into two sensor pairs, which measure the initial fluorescence state (*Fo*) and the maximum fluorescence state (*Fm*). Additionally, a CCD camera with a 512 × 512-pixel resolution (12-bit dynamic) was used to obtain the color scale images, which were used to determine the *Fv/Fm* values (0.1–1.0).

### 3.4. Hormone Testing Assay and Moss Cell Damage Evaluation

Three-week-old moss colonies were transferred to Hoagland solid media containing salicylic acid (Sigma, Sant Louis, MO, USA) at 0.1 mM and 0.5 mM, or jasmonic acid (Sigma) at 50 µM final concentrations. *P. patens*’ colonies were incubated during 24 h in the presence of salicylic acid or jasmonic acid, and after that period tissues were inoculated with *C. gloeosporioides* as previously described, and incubated for 72 h to evaluate cell damage using Evans Blue staining [59]. Briefly, moss colonies were incubated for 2 h in 0.1% Evans blue dye solution and washed four times with distilled water to remove the excess dye. To recover the intracellular Evans blue dye, moss colonies were washed with methanol (50%) and SDS (1%) for 30 min at 65 °C. The absorbance of the recovered solution was measured at 600 nm. Each sample consisted of four colonies incubated in 6 mL of the mixture methanol/SDS (50%:1%). Eight samples, corresponding to 32 colonies, were analyzed per experiment. Data are expressed as OD/mg dry weight of moss. Dry weight was determined after moss colonies were treated at 65 °C for 24 h.

A one-way analysis of variance (ANOVA) test was conducted to determine any statistically significant differences. Significance levels were always expressed as a value of *p* < 0.01. The data analysis software GraphPad Prism version 7.03 (GraphPad Software) was used.

### 3.5. RNA Extraction, cDNA Library Preparation and Sequencing

As mentioned before, 8- and 24-h-old co-cultures were recovered, frozen using liquid nitrogen and pulverized. Total RNA was extracted using the RNeasy Plant Mini Kit (Qiagen, Germany). RNA quality control, library preparation and sequencing were performed by Macrogen Inc. (Seoul, Korea). Briefly, RNA integrity number (RIN > 9) was checked using an Agilent Technologies 2100 Bioanalyzer (Agilent Technologies) and cDNA libraries were prepared for paired-end sequencing using 1 μg of RNA and the TruSeq Stranded Total RNA Plant LT Sample Prep. Finally, sequencing was performed on Illumina platform (Illumina, San Diego, CA, USA) to generate paired-end 150 bp reads, obtaining ~50 M reads per sample with Q_20_ > 97.5 %.

### 3.6. RNA-Seq Processing, Differential Expression Analysis and Gene Ontology Enrichment

The quality of the obtained sequences was checked using the FastQC software v0.10.1 [133]. Adaptors and low-quality sequences were removed using the Trimmomatic software v0.39 [134]. Additionally, using the default options, the following parameters were set: adapter sequence TruSeq3 (paired-ended, for MiSeq and HiSeq), HEADCROP: 10, and cutoff: Q_20_. Then the RSubread package version 3 [135] was used to sequence the alignment using the reference genome of *P. patens* (https://phytozome.jgi.doe.gov/pz/portal.html, accessed on 20 November 2019). In addition, the sequence alignment was also performed using *P. patens* available genomes of chloroplasts (NC_005087.1), mitochondria (NC_007945.1) and ribosomal RNA sequences (HM751653.1, X80986.1 and X98013.1).

To perform the differential expression analysis, genes with a low number of read counts (CPM < 0.5) were filtered, aiming to increase the statistical strength of the prediction of differentially expressed genes [136]. Expression data were normalized using the upper quartile method [137] for the removal of variation between samples, and differential expression analysis was performed using DESeq2 [138]. Genes with logFC > |2.0| and FDR < 0.05 were considered differentially expressed.

Gene Ontology (GO) enrichment was conducted following the protocol described by [139]. GO terms were obtained from The Arabidopsis Information Resource (TAIR) platform (https://www.arabidopsis.org/tools/go_term_enrichment.jsp, accessed on 15 April 2020), using the Panther classification system [140] on the *P. patens* database. To determine the enrichment rate, the Fischer’s Exact Test method was used. GO terms with an FDR < 0.05 were considered in this analysis. To summarize the long list of GO terms, redundant GO terms were removed with REViGO (reduce and visualize gene ontology) [141].

## 4. Final Remarks

**Figure 5:**
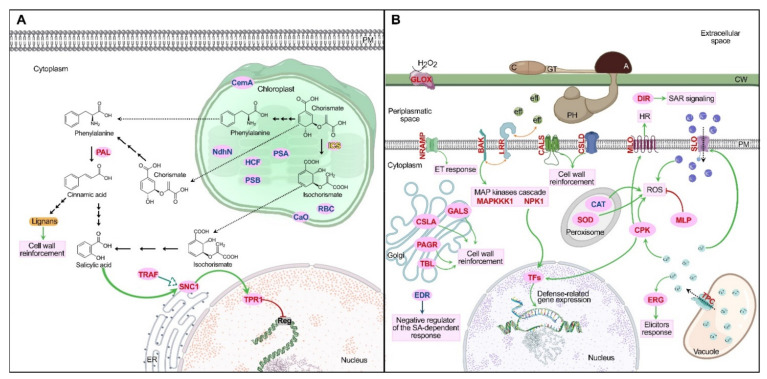
Proposed defense response in *P. patens* infected with *C. gloeosporioides*. (**A**) Salicylic acid synthesis mechanism and signaling pathway during the salicylic acid-dependent defense response; also, the downregulated genes from photosynthesis and chloroplast functions. (**B**) Defense strategies of *P. patens* during infection. Pink ovals indicate enzymes and proteins involved in the defense response. Red, blue and black letters indicate upregulated, downregulated and non-differentially expressed genes, respectively. Green arrows indicate activation, the red trunked line indicates inhibition, the double-headed orange arrows indicate possible interaction, the dotted arrows represent movement from an intracellular space to another and the consecutive black arrows indicate various intermediate reactions. C: conidium; GT: germination tube; A: appressorium; PH: primary hyphae; CW: cell wall; PM plasmatic membrane; HR: hypersensitive response; ROS: reactive oxygen species; ICS: Isochorismate synthase; MAPKKK1: Mitogen-activated protein kinase kinase kinase 1; NPK1: Mitogen-activated protein kinase kinase kinase NPK1; eff: fungal effectors. For other abbreviations, see the description of Figure 4.

In this study, we present a comprehensive description of the cellular and molecular response of the bryophyte *P. patens* during infection with the fungal pathogen *C. gloeosporioides*, which we depict in Figure 5. Although *P. patens* is susceptible to infection with *C. gloeosporioides*, once the pathogen is recognized by receptors, a defense response is activated, evidenced by the induction of genes involved in MAPK signaling cascades, ROS production and homeostasis, calcium signaling, an HR-like response, salicylic acid-dependent signaling, cell wall reinforcement and genes involved in different defense mechanisms. The defense response developed by *P. patens* coincides in several aspects with those observed in vascular plants. During the transcriptional reprogramming that occurs in *P. patens* during *C. gloeosporioides* colonization, several genes related to photosynthesis and chloroplast functioning are downregulated, indicating that the defense response and secondary metabolism processes are favored in moss tissues over metabolic functions of primary metabolism.

In conclusion, this study extends our knowledge of moss defense responses against pathogens and highlights the important molecular players during the evolution of land plant defense against biotic stress.

## Data Availability

Transcriptomic data was deposited in the Sequence Read Archive (SRA) from NCBI under SRA accession: PRJNA751102.

## Supplementary Materials

Available at https://www.mdpi.com/article/10.3390/jof7080677/s1. Table S1: Complete list of upregulated genes in *Physcomitrium patens* during infection by *Colletotrichum gloeosporioides*. Table S2: Complete list of downregulated genes in *Physcomitrium patens* during infection by *Colletotrichum gloeosporioides*. Table S3: Data of upregulated and downregulated genes represented in Figure 4.
