# Supplementary material for: Physcomitrium patens Infection by Colletotrichum gloeosporioides: Understanding the Fungal–Bryophyte Interaction by Microscopy, Phenomics and RNA Sequencing
Source: J Fungi (Basel). 2021 Aug 22;7(8):677. doi: 10.3390/jof7080677 (PMC8401727; doi:10.3390/jof7080677)
Supplement: Supplementary file 1 [file jof-07-00677-s001.zip › jof-1342788-supplementary.pdf]

Supporting Information Table S1: Complete list of upregulated genes in *Physcomitrium patens* during infection by *Colletotrichum gloeosporioides*.

Sequence ID	logFC	FDR	Description
Pp3c21_7680	8.40	1.50e-23	phenylalanine ammonia-lyase-like
Pp3c2_32410	8.19	4.58e-41	phenylalanine ammonia-lyase-like
Pp3c21_7670	8.18	1.18e-20	phenylalanine ammonia-lyase-like
Pp3c2_32330	7.87	1.12e-37	Ribonucleoside-diphosphate reductase large subunit
Pp3c19_10460	7.73	1.63e-39	---NA---
Pp3c27_3700	7.15	3.27e-40	hypothetical protein PHYPA_000021
Pp3c19_13690	7.04	5.45e-35	phenylalanine ammonia-lyase-like
Pp3c7_1110	6.98	3.63e-50	ADP-ribosylation factor GTPase-activating protein AGD1
Pp3c1_28050	6.98	5.12e-09	hypothetical protein PHYPA_011054
Pp3c7_1150	6.97	7.32e-50	TNF receptor-associated factor homolog 1a-like isoform X1
Pp3c24_13090	6.84	6.54e-14	U-box domain-containing protein 6-like
Pp3c11_25660	6.77	3.11e-25	uncharacterized protein LOC112284596
Pp3c2_36540	6.76	4.83e-70	E3 ubiquitin-protein ligase RNF14
Pp3c13_9000	6.73	3.00e-226	phenylalanine ammonia-lyase-like
Pp3c2_30610	6.67	7.51e-157	phenylalanine ammonia-lyase-like
Pp3c12_8070	6.66	5.89e-79	ANION TRANSPORTER 3, CHLOROPLASTIC-RELATED
Pp3c2_36410	6.66	3.90e-70	hypothetical protein PHYPA_008387
Pp3c2_36290	6.66	7.84e-69	hypothetical protein PHYPA_022281, partial
Pp3c4_16570	6.63	1.42e-41	proline-, glutamic acid- and leucine-rich protein 1 isoform X4
Pp3c2_36170	6.61	5.28e-128	protein COP1 SUPPRESSOR 2-like
Pp3c2_32320	6.61	7.92e-44	protein HGH1 homolog
Pp3c22_15350	6.59	4.00e-50	afadin- and alpha-actinin-binding protein-like isoform X1
Pp3c2_36440	6.57	9.82e-169	1-acyl-sn-glycerol-3-phosphate acyltransferase 2
Pp3c2_36350	6.56	7.85e-61	elicitor-responsive protein 3-like
Pp3c16_630	6.50	2.39e-248	SAC3 family protein C isoform X1
Pp3c12_9140	6.43	6.58e-104	hypothetical protein PHYPA_019779
Pp3c19_13170	6.41	5.15e-182	hypothetical protein PHYPA_025245
Pp3c2_32400	6.39	8.19e-60	ethylene-responsive transcription factor RAP2-11-like
Pp3c1_18830	6.37	3.04e-185	phenylalanine ammonia-lyase-like
Pp3c1_18680	6.35	1.28e-169	---NA---
Pp3c1_18940	6.33	3.22e-176	phenylalanine ammonia-lyase-like
Pp3c6_6610	6.28	7.14e-18	filaggrin-like isoform X1
Pp3c3_21190	6.22	6.62e-61	probable leucine-rich repeat receptor-like protein kinase At1g68400
Pp3c6_6560	6.19	1.45e-18	polygalacturonate 4-alpha-galacturonosyltransferase

Pp3c19_6250	6.19	2.28e-66	Rubredoxin 1
Pp3c19_6320	6.16	7.95e-189	aromatic aminotransferase ISS1-like
Pp3c24_18010	6.15	6.05e-191	hypothetical protein PHYPA_018527
Pp3c19_6240	6.14	3.89e-169	uncharacterized protein LOC112273189
Pp3c18_17330	6.13	7.70e-49	probable protein S-acyltransferase 22
Pp3c14_22890	6.12	1.49e-190	hypothetical protein PHYPA_016993
Pp3c24_18000	6.11	7.31e-176	Transposon TX1 uncharacterized
Pp3c19_6330	6.10	5.48e-169	ent-kaurene oxidase-like 3
Pp3c11_2950	6.07	1.93e-203	TPR repeat-containing protein
Pp3c19_9000	6.00	7.42e-182	isoleucine--tRNA ligase, chloroplastic/mitochondrial
Pp3c3_27000	5.94	2.73e-78	inactive poly [ADP-ribose] polymerase RCD1-like isoform X1
Pp3c1_28020	5.94	6.72e-10	probable inactive purple acid phosphatase 27
Pp3c2_30370	5.88	2.49e-127	nucleolar and coiled-body phosphoprotein 1-like
Pp3c3_6500	5.85	4.84e-13	mitogen-activated protein kinase kinase kinase NPK1-like
Pp3c2_30620	5.78	1.28e-141	nucleosome assembly protein 1;3-like isoform X5
Pp3c8_4130	5.72	1.81e-51	pyruvate dehydrogenase E1 component subunit alpha-3, chloroplastic
Pp3c24_2840	5.71	2.79e-67	uncharacterized aarF domain-containing protein kinase At1g79600, chloroplastic-like
Pp3c22_10130	5.70	9.65e-147	Holo-ACP Synthase
Pp3c23_940	5.67	6.68e-15	187-kDa microtubule-associated protein AIR9-like
Pp3c10_21310	5.67	1.45e-108	serine protease inhibitor, Kazal-type family protein
Pp3c21_16620	5.62	9.45e-82	hypothetical protein PHYPA_030933
Pp3c6_4000	5.61	2.97e-43	conserved oligomeric Golgi complex subunit 4
Pp3c21_20650	5.61	1.49e-130	hypothetical protein PHYPA_031097
Pp3c13_24240	5.55	1.62e-120	choline-phosphate cytidylyltransferase 2-like
Pp3c7_22740	5.54	9.23e-52	DEAD-box ATP-dependent RNA helicase 46
Pp3c12_25330	5.53	6.33e-27	probable sodium/metabolite cotransporter BASS1, chloroplastic
Pp3c13_17510	5.46	2.03e-16	UDP-arabinopyranose mutase 2-like
Pp3c17_10090	5.38	3.71e-10	Regulator of rDNA transcription protein 15
Pp3c17_21250	5.34	6.47e-27	uncharacterized protein LOC112279540 isoform X3
Pp3c13_17480	5.32	4.90e-16	hypothetical protein PHYPA_020162
Pp3c12_11940	5.28	3.70e-60	bZIP transcription factor 60-like
Pp3c13_17633	5.27	8.98e-14	beta-mannosyltransferase 1
Pp3c17_10120	5.27	1.42e-12	hypothetical protein PHYPA_023918

Pp3c17_21300	5.25	3.74e-24	hypothetical protein PHYPA_004729
Pp3c4_23930	5.24	2.22e-78	hybrid signal transduction histidine kinase K-like
Pp3c2_29800	5.24	7.21e-175	uncharacterized protein LOC112283113
Pp3c17_21180	5.23	6.27e-23	putative methyltransferase domain protein
Pp3c20_18690	5.22	4.96e-116	NADH dehydrogenase [ubiquinone] iron-sulfur protein 6, mitochondrial
Pp3c12_6960	5.21	1.60e-50	zinc finger CCCH domain-containing protein 19-like
Pp3c17_21150	5.17	4.80e-22	hypothetical protein PHYPA_004720
Pp3c2_35740	5.17	1.13e-137	uncharacterized protein LOC112283107
Pp3c3_18090	5.16	9.94e-14	hypothetical protein PHYPA_029859
Pp3c19_13030	5.14	9.41e-84	hypothetical protein PHYPA_025239
Pp3c18_4480	5.09	2.54e-143	auxin transporter-like protein 2 isoform X1
Pp3c13_17813	5.08	3.65e-15	protein FAR1-RELATED SEQUENCE 5-like
Pp3c2_27270	4.93	9.71e-151	probable 18S rRNA (guanine-N(7))-methyltransferase
Pp3c17_3860	4.93	3.01e-10	transcription factor TCP15-like
Pp3c17_14850	4.89	4.60e-120	hypothetical protein PHYPA_004408, partial
Pp3c3_19730	4.89	2.90e-13	protein trichome birefringence-like 11 isoform X1
Pp3c19_21220	4.87	3.64e-78	hypothetical protein PHYPA_024481
Pp3c10_25030	4.87	2.44e-26	peptidyl-prolyl cis-trans isomerase CYP40-like
Pp3c16_6800	4.85	2.40e-55	cell number regulator 8-like isoform X2
Pp3c12_7050	4.85	1.03e-48	hypothetical protein PHYPA_019159
Pp3c26_2420	4.83	3.04e-91	phenylalanine ammonia-lyase-like
Pp3c19_6660	4.78	4.66e-78	Ubiquitin and WLM domain-containing protein C1442.07c, putative
Pp3c11_21367	4.77	5.51e-05	hypothetical protein PHYPA_005580, partial
Pp3c17_5320	4.77	1.50e-117	squamosa promoter-binding-like protein 15 isoform X4
Pp3c20_18520	4.74	1.12e-08	Retrovirus-related Pol polyprotein from transposon TNT 1-94
Pp3c6_2730	4.73	2.30e-59	transcription factor bHLH66 isoform X1
Pp3c22_14800	4.71	3.42e-46	enoyl-[acyl-carrier-protein] reductase, mitochondrial
Pp3c27_4100	4.71	1.17e-35	serine/arginine repetitive matrix protein 2-like isoform X1
Pp3c6_1770	4.70	8.37e-21	phosphoribosylamine--glycine ligase, chloroplastic
Pp3c13_24020	4.69	3.55e-46	proline-rich protein PRCC
Pp3c23_11380	4.68	1.22e-117	hypothetical protein PHYPA_015014
Pp3c1_16900	4.66	1.23e-109	hypothetical protein PHYPA_018185
Pp3c12_18710	4.65	8.06e-51	ALA-interacting subunit 3
Pp3c11_14690	4.64	4.36e-102	hypothetical protein PHYPA_005683
Pp3c5_1740	4.64	9.67e-75	sister chromatid cohesion protein PDS5 homolog A

Pp3c18_1530	4.60	2.01e-75	homeobox protein prospero-like isoform X1
Pp3c13_12560	4.60	2.89e-09	protein NRT1/ PTR FAMILY 8.1-like
Pp3c8_16520	4.55	5.27e-60	hypothetical protein PHYPA_012862, partial
Pp3c3_8160	4.55	1.66e-85	hypothetical protein PHYPA_022795
Pp3c2_37330	4.53	3.54e-47	transmembrane protein 256 homolog
Pp3c3_6540	4.52	9.24e-126	hypothetical protein PHYPA_022747
Pp3c20_16410	4.52	1.77e-51	magnesium chelatase subunit of protochlorophyllide reductase (chloroplast)
Pp3c13_14480	4.51	1.11e-249	predicted protein
Pp3c7_13100	4.51	1.11e-18	serine/threonine-protein kinase HT1
Pp3c3_8120	4.50	5.31e-79	lipase 1-like isoform X2
Pp3c12_2640	4.47	4.76e-92	carbamoyl-phosphate synthase small chain, chloroplastic
Pp3c3_8080	4.47	2.36e-79	mitochondrial arginine transporter BAC2-like
Pp3c16_23390	4.47	6.23e-50	probable aspartyl aminopeptidase isoform X1
Pp3c5_15640	4.47	1.02e-223	ribonuclease H2 subunit B
Pp3c23_21700	4.47	1.55e-21	truncated basic helix-loop-helix protein A-like
Pp3c18_1550	4.46	1.26e-32	probable pectinesterase 53
Pp3c15_19310	4.46	2.86e-42	Protein ROS1
Pp3c27_3620	4.39	2.89e-65	calmodulin-binding receptor-like cytoplasmic kinase 2 isoform X4
Pp3c7_19630	4.38	3.93e-53	histone-lysine N-methyltransferase ATXR6-like
Pp3c7_17000	4.38	8.40e-40	transcription factor MYB21-like
Pp3c3_14160	4.35	1.27e-51	dual-specificity tyrosine-(Y)-phosphorylation regulated kinase
Pp3c10_15520	4.35	1.18e-115	---NA---
Pp3c4_27720	4.35	1.11e-38	phosphoenolpyruvate carboxylase 2
Pp3c21_2960	4.33	1.71e-95	protein TIFY 6a-like
Pp3c4_24020	4.32	1.78e-28	uncharacterized protein YEL023C-like isoform X1
Pp3c21_7830	4.31	3.20e-79	110 kDa U5 small nuclear ribonucleoprotein component CLO-like
Pp3c4_25770	4.31	4.66e-100	preprotein translocase subunit SCY1, chloroplastic
Pp3c2_24900	4.30	1.64e-27	NADH dehydrogenase ubiquinone 1 alpha subcomplex assembly factor-like protein (DUF498/DUF598)
Pp3c18_21960	4.28	3.30e-95	heavy metal-associated isoprenylated plant protein 30-like
Pp3c8_25020	4.28	3.50e-16	proteasome subunit beta type-2-A
Pp3c10_17120	4.27	8.61e-59	probable E3 ubiquitin-protein ligase XBOS32 isoform X1
Pp3c4_17790	4.26	6.44e-47	carbonic anhydrase, chloroplastic-like
Pp3c1_7800	4.26	1.16e-88	protein DA1-related 1-like

Pp3c6_6330	4.22	2.64e-224	60S ribosomal protein L15
Pp3c5_10720	4.19	2.25e-105	clathrin interactor 1-like
Pp3c2_24761	4.19	8.32e-92	hypothetical protein PHYPA_015829
Pp3c1_23380	4.18	2.01e-31	calphotin-like isoform X1
Pp3c19_17320	4.18	6.45e-14	dormancy/auxin associated-like protein
Pp3c26_4070	4.18	1.69e-63	hypothetical protein PHYPA_000911
Pp3c7_20170	4.17	2.97e-43	hypothetical protein PHYPA_029087
Pp3c19_18700	4.15	1.59e-80	30S ribosomal protein S21, chloroplastic-like
Pp3c19_6710	4.15	1.24e-61	disease resistance protein TAO1-like
Pp3c3_16960	4.15	8.36e-30	hypothetical protein PHYPA_029799
Pp3c12_7720	4.11	2.04e-23	BI1-like protein
Pp3c10_23730	4.09	1.36e-51	auxin-responsive protein SAUR72-like
Pp3c1_13930	4.08	1.54e-106	uncharacterized protein LOC112291597
Pp3c23_5150	4.07	4.33e-51	beta-glucosidase 4
Pp3c14_3390	4.07	8.83e-61	hypothetical protein PHYPA_002494
Pp3c5_5670	4.06	2.39e-18	fibronectin type-III domain-containing protein 3a isoform X2
Pp3c8_22400	4.05	1.08e-117	superoxide dismutase [Cu-Zn]-like
Pp3c26_3030	4.03	1.16e-54	hypothetical protein PHYPA_000862
Pp3c2_17270	4.02	8.93e-28	calcium-dependent protein kinase 26-like
Pp3c25_10190	4.00	3.10e-94	dnaJ homolog subfamily C member 2-like
Pp3c14_11550	4.00	1.53e-84	protein MOTHER of FT and TFL1-like isoform X1
Pp3c2_25760	4.00	3.54e-17	protein PECTIC ARABINOGALACTAN SYNTHESIS-RELATED-like isoform X1
Pp3c16_18830	3.97	1.91e-104	ERAD-associated E3 ubiquitin-protein ligase component HRD3A
Pp3c8_5110	3.97	4.72e-29	Zinc transport protein like
Pp3c24_16410	3.96	6.73e-41	regulation of nuclear pre-mRNA domain-containing protein 1B-like
Pp3c26_8040	3.95	1.65e-69	guanine nucleotide exchange factor SPIKE 1-like isoform X1
Pp3c26_4770	3.95	2.85e-35	YTH domain-containing protein 1-like
Pp3c23_10130	3.94	3.04e-50	coatomer subunit alpha-1
Pp3c22_12740	3.94	1.65e-74	hypothetical protein PHYPA_007965
Pp3c7_25930	3.93	1.13e-80	3-dehydrosphinganine reductase TSC10A-like
Pp3c11_25880	3.93	3.26e-134	DNA/pantothenate metabolism flavoprotein, C-terminal
Pp3c23_14050	3.92	5.11e-05	uncharacterized protein LOC112288668 isoform X3
Pp3c1_19060	3.90	4.04e-75	transmembrane protein, putative
Pp3c15_23190	3.89	7.06e-09	---NA---
Pp3c23_17280	3.88	6.39e-32	protein SCAI-like isoform X1
Pp3c24_19980	3.88	4.53e-26	transcription factor HEC2
Pp3c19_17300	3.86	6.06e-56	tubby-like F-box protein 3 isoform X1
Pp3c26_13490	3.85	2.26e-56	glycerol-3-phosphate dehydrogenase [NAD(+)] 2, chloroplastic isoform X1

Pp3c6_520	3.85	6.14e-27	kinesin-like protein KIN-14I isoform X1
Pp3c7_22440	3.85	3.10e-15	probable glycerol-3-phosphate dehydrogenase [NAD(+)] 2, cytosolic
Pp3c17_1970	3.85	6.25e-33	ribulose bisphosphate carboxylase small chain clone 512-like
Pp3c16_6520	3.84	2.29e-57	hypothetical protein PHYPA_023730
Pp3c12_9940	3.83	2.01e-24	hypothetical protein PHYPA_019808
Pp3c7_250	3.80	5.84e-50	beach domain-containing protein
Pp3c3_32210	3.79	1.83e-47	eukaryotic translation initiation factor 3 subunit K
Pp3c7_14010	3.79	7.89e-19	rab3 GTPase-activating protein catalytic subunit
Pp3c26_4220	3.78	3.26e-71	F-box/kelch-repeat protein At1g15670-like
Pp3c15_10550	3.75	5.27e-33	hypothetical protein PHYPA_002854
Pp3c3_10280	3.75	5.16e-36	probable protein phosphatase 2C 22
Pp3c12_19430	3.75	8.13e-178	Retrovirus-related Pol polyprotein from transposon RE1
Pp3c19_9970	3.75	2.71e-66	uncharacterized oxidoreductase At4g09670-like
Pp3c22_16360	3.74	3.37e-35	auxin-responsive protein SAUR72-like
Pp3c10_5660	3.74	1.26e-62	caltractin
Pp3c15_15960	3.73	1.21e-17	importin-11 isoform X1
Pp3c5_880	3.73	3.86e-30	WD repeat-containing protein LWD1
Pp3c15_16100	3.72	7.22e-58	hypothetical protein PHYPA_003086
Pp3c23_9170	3.72	2.04e-35	phospho-2-dehydro-3-deoxyheptonate aldolase 2, chloroplastic-like
Pp3c15_5450	3.70	3.39e-21	sorting nexin 2A-like
Pp3c8_24690	3.70	9.93e-21	uncharacterized protein LOC112287004
Pp3c6_9060	3.69	1.93e-63	protein FAM63B-like
Pp3c6_11170	3.67	8.65e-33	hypothetical protein PHYPA_027418
Pp3c27_8590	3.67	3.57e-21	tobamovirus multiplication protein 3-like
Pp3c10_9790	3.67	4.32e-22	uncharacterized protein LOC112280954
Pp3c18_11980	3.66	4.12e-24	3-ketoacyl-CoA thiolase 2, peroxisomal
Pp3c8_3600	3.66	2.80e-101	hypothetical protein PHYPA_012297
Pp3c21_7310	3.66	2.71e-30	serine/arginine-rich SC35-like splicing factor SCL30A
Pp3c10_10860	3.66	3.37e-111	uncharacterized protein LOC112278155
Pp3c10_24760	3.65	1.14e-57	hypothetical protein PHYPA_006195
Pp3c4_14550	3.65	1.28e-68	hypothetical protein PHYPA_020925
Pp3c27_1870	3.64	3.93e-28	probable copper-transporting ATPase HMA5
Pp3c12_2240	3.64	4.51e-18	probable sugar phosphate/phosphate translocator At3g11320
Pp3c15_7520	3.63	1.33e-48	hypothetical protein PHYPA_003742
Pp3c4_21680	3.62	6.18e-90	hypothetical protein PHYPA_021403
Pp3c20_15490	3.62	3.14e-37	---NA---
Pp3c18_15840	3.61	5.34e-146	hypothetical protein PHYPA_005417
Pp3c13_16440	3.60	2.03e-09	glycosyltransferase in CAZy family GT78

Pp3c15_6310	3.60	5.09e-32	hypothetical protein PHYPA_003691
Pp3c4_5170	3.60	1.04e-50	retrovirus-related pol polyprotein from transposon tnt 1-94
Pp3c17_21600	3.57	9.12e-30	hypothetical protein PHYPA_004746
Pp3c1_22560	3.56	1.12e-70	hypothetical protein PHYPA_011699
Pp3c19_7630	3.56	5.54e-90	hypothetical protein PHYPA_024947
Pp3c13_21030	3.55	1.19e-30	enolase 1, chloroplastic
Pp3c1_10860	3.54	1.26e-57	threonine--tRNA ligase, chloroplastic/mitochondrial 2
Pp3c2_12680	3.54	6.37e-84	UBP1-associated protein 2C-like
Pp3c21_16540	3.52	4.20e-27	hypothetical protein PHYPA_030929
Pp3c17_22550	3.51	1.24e-21	afadin- and alpha-actinin-binding protein-like isoform X1
Pp3c2_1870	3.51	1.00e-10	hypothetical protein PHYPA_016564
Pp3c1_24550	3.49	6.98e-21	anamorsin homolog
Pp3c1_34890	3.48	5.57e-17	GPI mannosyltransferase 1-like
Pp3c8_18470	3.47	3.64e-24	fimbrin-1
Pp3c12_8440	3.47	1.89e-136	hypothetical protein PHYPA_019170, partial
Pp3c10_11730	3.47	3.02e-52	vesicle-associated membrane protein 714-like
Pp3c5_23790	3.46	6.41e-05	DExH-box ATP-dependent RNA helicase DExH10
Pp3c12_17520	3.46	1.88e-17	hypothetical protein PHYPA_009814
Pp3c6_18950	3.46	4.30e-69	hypothetical protein PHYPA_026916
Pp3c7_1960	3.46	2.34e-13	molybdopterin synthase sulfur carrier subunit
Pp3c3_24240	3.46	2.77e-50	predicted protein
Pp3c11_10350	3.44	3.20e-51	hypothetical protein PHYPA_006610
Pp3c1_32560	3.43	1.10e-75	Clathrin, heavy chain
Pp3c9_3690	3.43	9.03e-19	hypothetical protein PHYPA_025859
Pp3c21_20950	3.42	4.83e-71	CLPTM1-like membrane protein cnrB
Pp3c1_29970	3.41	3.47e-55	Immunoglobulin-like protein
Pp3c18_5000	3.40	3.01e-23	---NA---
Pp3c21_21000	3.40	3.18e-65	predicted protein
Pp3c9_17830	3.39	8.75e-19	actin-related protein 3
Pp3c21_9770	3.39	1.51e-75	hypothetical protein PHYPA_007850
Pp3c4_17500	3.39	1.37e-181	hypothetical protein PHYPA_021207
Pp3c20_1301	3.38	1.45e-03	---NA---
Pp3c5_28420	3.38	1.52e-07	polyadenylate-binding protein 2-like isoform X1
Pp3c5_22560	3.37	1.91e-45	2-isopropylmalate synthase
Pp3c3_28140	3.37	1.12e-45	hypothetical protein PHYPA_022950
Pp3c1_9320	3.37	3.20e-92	receptor kinase-like protein
Pp3c8_24170	3.37	1.48e-63	Retrovirus-related Pol polyprotein from transposon TNT 1-94
Pp3c1_10460	3.36	9.04e-34	60S ribosomal protein L22-3
Pp3c22_22960	3.35	8.67e-41	putative RING-H2 finger protein ATL69
Pp3c6_12700	3.34	5.97e-13	predicted protein

Pp3c4_23320	3.33	3.56e-29	hypothetical protein PHYPA_021457
Pp3c7_12470	3.33	1.86e-32	hypothetical protein PHYPA_021773
Pp3c4_24420	3.33	8.17e-22	protein LURP-one-related 15-like
Pp3c22_15880	3.33	6.15e-18	protein translation factor SUI1 homolog
Pp3c2_28140	3.33	4.59e-43	uncharacterized protein LOC112289464
Pp3c1_7740	3.32	3.12e-29	hypothetical protein PHYPA_000398
Pp3c19_6110	3.32	5.20e-16	hypothetical protein PHYPA_024875
Pp3c4_22080	3.32	9.28e-163	putative helicase
Pp3c11_6920	3.31	1.07e-30	hypothetical protein PHYPA_010450
Pp3c21_10290	3.31	3.14e-39	NADP-dependent glyceraldehyde-3-phosphate dehydrogenase-like
Pp3c20_1460	3.31	8.33e-33	transcriptional regulator, MarR family
Pp3c22_5710	3.30	2.33e-11	cold-shock DNA binding protein
Pp3c18_19000	3.30	6.78e-30	hypothetical protein PHYPA_005534
Pp3c5_3280	3.29	2.42e-50	bifunctional protein FolD 4, chloroplastic-like
Pp3c14_7860	3.29	3.79e-22	carbonic anhydrase 2-like isoform X1
Pp3c24_14470	3.29	3.27e-17	hypothetical protein PHYPA_018369
Pp3c2_9700	3.29	1.46e-38	NADH-ubiquinone oxidoreductase 20.9 kDa subunit-like
Pp3c5_23450	3.28	1.51e-11	hypothetical protein PHYPA_013536
Pp3c11_13030	3.28	2.68e-18	signal peptide peptidase-like 1
Pp3c18_6640	3.27	4.20e-20	DExH-box ATP-dependent RNA helicase DExH11
Pp3c20_10830	3.25	1.03e-76	hypothetical protein OsI_14862
Pp3c20_12200	3.25	1.06e-14	---NA---
Pp3c20_88	3.25	3.27e-04	---NA---
Pp3c5_13990	3.25	5.24e-11	protein SUPPRESSOR OF npr1-1, CONSTITUTIVE 1-like
Pp3c10_1100	3.25	5.41e-36	xyloglucan galactosyltransferase KATAMARI1 homolog
Pp3c2_27350	3.24	2.10e-41	citrate synthase, glyoxysomal
Pp3c6_2050	3.24	1.40e-57	EPIDERMAL PATTERNING FACTOR-like protein 6
Pp3c15_17290	3.24	8.69e-05	hypothetical protein PHYPA_003134, partial
Pp3c4_15690	3.24	1.61e-39	hypothetical protein PHYPA_020979
Pp3c2_29750	3.23	5.14e-11	acetate kinase
Pp3c14_5500	3.22	6.38e-18	LRR receptor-like serine/threonine-protein kinase GSO1 isoform X1
Pp3c26_12740	3.22	6.76e-44	---NA---
Pp3c26_4040	3.22	7.08e-63	---NA---
Pp3c8_21080	3.21	1.19e-54	hypothetical protein PHYPA_013091
Pp3c1_26930	3.21	3.98e-18	light-sensor Protein kinase-like
Pp3c26_3430	3.21	4.85e-14	pro-resilin-like isoform X2
Pp3c9_19340	3.21	3.84e-04	uncharacterized protein LOC112273996 isoform X1
Pp3c8_16270	3.20	9.64e-39	F-box protein SKIP31
Pp3c8_4960	3.20	3.68e-101	uncharacterized protein LOC112286649

Pp3c15_17690	3.19	3.61e-47	Putative ribonuclease H protein
Pp3c2_27000	3.18	5.75e-27	calcium-activated potassium channel slowpoke-like isoform X1
Pp3c18_2960	3.18	2.01e-04	eukaryotic translation initiation factor 3 subunit C-like
Pp3c10_17670	3.18	1.85e-04	hypothetical protein PHYPA_031247
Pp3c14_20030	3.18	1.15e-13	peroxidase N1-like
Pp3c18_22120	3.18	1.37e-44	plasma membrane ATPase
Pp3c12_14480	3.18	3.05e-120	SH2 domain-containing protein A
Pp3c7_26250	3.17	2.15e-04	60S ribosomal protein L4-like
Pp3c26_2830	3.17	1.50e-15	hypothetical protein PHYPA_000852, partial
Pp3c16_20970	3.17	1.68e-11	putative alpha/Beta hydrolase fold protein
Pp3c17_7090	3.17	4.36e-57	transcription initiation factor TFIID subunit 14b
Pp3c7_20910	3.16	4.94e-32	hemolysin A, putative
Pp3c26_40	3.16	1.71e-04	hypothetical protein PHYPA_000906
Pp3c10_16800	3.15	1.21e-22	AP-1 complex subunit gamma-2 isoform X1
Pp3c22_20830	3.15	2.51e-13	BTB/POZ domain-containing protein At1g63850-like
Pp3c17_22500	3.15	2.50e-13	histone H2A.6-like
Pp3c3_22480	3.15	1.29e-79	hypothetical protein PHYPA_029540
Pp3c27_8400	3.15	8.41e-15	Molybdenum cofactor sulfurase 3
Pp3c14_22090	3.14	1.49e-24	glycerophosphodiester phosphodiesterase GDPD1, chloroplastic
Pp3c16_7080	3.14	1.04e-13	hypothetical protein PHYPA_023764
Pp3c7_15740	3.13	1.56e-04	hypothetical protein PHYPA_028898
Pp3c14_3310	3.13	9.67e-23	OBP32pep, putative (DUF220)
Pp3c14_6110	3.13	3.17e-04	uncharacterized protein LOC112273006 isoform X1
Pp3c18_3130	3.12	1.64e-39	transmembrane 9 superfamily member 7
Pp3c26_3070	3.11	5.71e-46	hypothetical protein PHYPA_000864
Pp3c25_580	3.11	3.13e-17	hypothetical protein PHYPA_011861
Pp3c16_19220	3.11	9.28e-24	probable transcription factor PosF21
Pp3c3_740	3.11	1.50e-14	RING-H2 finger protein ATL73-like
Pp3c22_8523	3.10	8.33e-15	40S ribosomal protein S13
Pp3c8_10260	3.10	5.96e-91	ABC transporter B family member 25
Pp3c7_3560	3.10	2.33e-11	autophagy-related protein 18h-like isoform X1
Pp3c13_16620	3.10	1.80e-50	hypothetical protein PHYPA_020128
Pp3c20_4190	3.09	3.25e-15	phi class glutathione S-transferase
Pp3c18_20140	3.09	3.89e-34	RING finger protein
Pp3c6_3720	3.08	5.30e-87	alcohol dehydrogenase class-3
Pp3c20_21833	3.08	3.40e-43	hypothetical protein PHYPA_028133, partial
Pp3c24_30	3.08	2.29e-15	MFT-like protein
Pp3c4_26880	3.08	1.77e-29	pentatricopeptide repeat-containing protein At2g35130
Pp3c20_21820	3.08	6.20e-43	Zinc knuckle CX2CX4HX4C

Pp3c20_396	3.07	2.28e-04	glyceraldehyde-3-phosphate dehydrogenase, cytosolic-like
Pp3c7_19830	3.07	1.18e-37	golgin subfamily A member 6-like protein 22
Pp3c7_19850	3.07	2.33e-37	hypothetical protein PHYPA_029069
Pp3c6_13990	3.07	3.41e-11	receptor kinase-like protein
Pp3c4_19490	3.06	2.64e-44	7-deoxyloganetin glucosyltransferase-like
Pp3c6_17010	3.06	2.78e-41	GDSL esterase/lipase At4g10955 isoform X2
Pp3c3_27280	3.06	8.68e-15	probable folate-biopterin transporter 2
Pp3c3_12890	3.06	5.64e-40	putative protease Do-like 14 isoform X1
Pp3c26_2670	3.05	3.35e-10	DUF724 domain-containing protein
Pp3c7_12530	3.05	1.78e-12	hypothetical protein PHYPA_021777
Pp3c15_5890	3.05	1.20e-101	lysine-specific histone demethylase 1 homolog 3-like isoform X1
Pp3c5_3730	3.05	3.50e-16	uncharacterized protein LOC112274973
Pp3c25_6760	3.04	2.20e-23	LRR receptor kinase BAK1
Pp3c26_2600	3.04	2.16e-12	mannose/glucose-specific lectin-like
Pp3c26_1520	3.04	2.09e-09	myb-like protein Q
Pp3c22_20810	3.04	2.26e-14	protein SPIRRIG-like
Pp3c26_1670	3.03	6.51e-10	40S ribosomal protein S10-1-like
Pp3c4_31020	3.03	1.11e-03	pleckstrin homology domain-containing protein 1-like
Pp3c24_14400	3.03	9.92e-34	uncharacterized protein LOC112290983
Pp3c7_600	3.02	1.08e-12	carbonic anhydrase, chloroplastic-like
Pp3c23_15050	3.01	5.93e-51	hypothetical protein PHYPA_015203
Pp3c5_9550	3.00	2.68e-65	hypothetical protein PHYPA_027345
Pp3c15_2490	2.99	7.02e-52	cytochrome c heme attachment protein (chloroplast)
Pp3c6_270	2.99	5.15e-11	F-box protein At5g39450 isoform X2
Pp3c26_1680	2.99	1.76e-09	phosphoadenosine phosphosulphate reductase nuclear
Pp3c1_6050	2.99	1.53e-84	probable chalcone--flavonone isomerase 3
Pp3c25_12820	2.98	3.21e-09	hypothetical protein PHYPA_018180
Pp3c23_11581	2.98	4.19e-14	mediator of RNA polymerase II transcription subunit 12
Pp3c7_6500	2.98	3.71e-80	uncharacterized protein LOC112276755 isoform X1
Pp3c1_33600	2.97	8.15e-15	multiple myeloma tumor-associated protein 2 homolog
Pp3c10_3170	2.97	2.97e-32	uncharacterized protein LOC112281426 isoform X1
Pp3c15_3560	2.96	1.94e-90	eukaryotic translation initiation factor 4E-1
Pp3c14_17840	2.96	7.05e-78	protein DMP4-like
Pp3c14_8460	2.96	3.38e-12	putative abnormal suspensor 1
Pp3c3_32650	2.96	7.02e-67	putative UPF0481 protein At3g02645
Pp3c24_4420	2.96	6.71e-29	uncharacterized protein LOC112291007
Pp3c4_6640	2.95	7.56e-26	ras-related protein RABA5e

Pp3c20_19230	2.95	4.48e-05	two-component response regulator ARR15-like
Pp3c2_16250	2.94	3.45e-22	60S ribosomal protein L38
Pp3c9_16970	2.94	7.59e-45	ethylene-responsive transcription factor ERF039-like
Pp3c3_26310	2.93	7.81e-13	hypothetical protein PHYPA_029718
Pp3c10_20210	2.93	3.13e-83	non-specific lipid transfer protein GPI-anchored 2
Pp3c8_22460	2.93	2.51e-04	p21-activated protein kinase-interacting protein 1-like
Pp3c7_24490	2.93	5.62e-55	purple acid phosphatase 23 isoform X1
Pp3c9_11330	2.93	2.15e-16	two pore calcium channel protein 1-like isoform X1
Pp3c23_5980	2.92	6.58e-10	mitochondrial inner membrane protein OXA1-like
Pp3c19_9280	2.92	8.97e-04	probable copper-transporting ATPase HMA5
Pp3c5_4140	2.91	3.12e-84	CASP-like protein 1C1
Pp3c23_11590	2.91	2.96e-09	flavin-containing monooxygenase FMO GS-OX-like 4
Pp3c16_8340	2.91	4.91e-13	transducin beta-like protein 2
Pp3c11_10560	2.91	1.39e-19	trihelix transcription factor ASR3-like
Pp3c19_20160	2.91	4.80e-24	zinc finger CCCH domain-containing protein 20
Pp3c4_23600	2.90	1.06e-13	hypothetical protein PHYPA_020385
Pp3c1_10630	2.90	5.00e-24	uncharacterized protein LOC112288147 isoform X1
Pp3c20_19460	2.89	9.99e-07	coiled-coil domain-containing protein 94 homolog
Pp3c19_21390	2.89	2.01e-14	protein SUPPRESSOR OF npr1-1, CONSTITUTIVE 1-like isoform X1
Pp3c7_10610	2.89	8.56e-33	U-box domain-containing protein 13
Pp3c1_2740	2.88	1.19e-16	hypothetical protein PHYPA_011990
Pp3c19_18410	2.88	1.29e-12	ribosomal protein S2 (mitochondrion)
Pp3c15_8180	2.87	2.77e-38	uncharacterized protein LOC112278689 isoform X1
Pp3c18_18780	2.86	5.97e-17	BTB/POZ domain-containing protein At1g67900-like
Pp3c3_18290	2.86	1.71e-62	glutamate--tRNA ligase, cytoplasmic
Pp3c16_5590	2.85	1.06e-48	EEF1A lysine methyltransferase 2-like
Pp3c27_3810	2.85	1.42e-41	protein LONGIFOLIA 1-like
Pp3c24_12310	2.85	7.12e-15	UDP-galactose/UDP-glucose transporter 3
Pp3s37_140	2.84	6.10e-16	histidine kinase 4
Pp3c11_10320	2.84	2.92e-18	hypothetical protein PHYPA_006610
Pp3c2_33330	2.84	9.12e-09	uracil-DNA glycosylase
Pp3c9_14080	2.83	5.17e-26	disease resistance protein TAO1-like
Pp3c17_14100	2.83	9.86e-52	hypothetical protein PHYPA_004364
Pp3c8_8380	2.82	4.18e-51	hypothetical protein PHYPA_012485

Pp3c26_710	2.82	4.91e-09	putative E3 ubiquitin ligase SUD1
Pp3c16_2360	2.82	1.58e-33	ribosomal-like protein, putative
Pp3c8_16240	2.82	9.19e-10	trafficking protein particle complex subunit 4-like
Pp3c15_20210	2.81	8.32e-13	basic leucine zipper 61-like isoform X1
Pp3c10_12290	2.81	2.31e-14	endoglucanase 6-like
Pp3c21_16130	2.80	1.42e-06	AAA-type ATPase-like
Pp3c16_10020	2.80	2.03e-06	LOB domain-containing protein 10
Pp3c23_22200	2.80	1.32e-13	transposon protein, putative, CACTA, En/Spm sub-class
Pp3c24_2390	2.79	2.32e-37	40S ribosomal protein S10-1-like
Pp3c16_13150	2.79	1.96e-63	cyclin-A1-4-like
Pp3c1_15390	2.79	4.55e-59	RNA polymerase sigma factor sigA
Pp3c2_27310	2.78	3.71e-96	3-oxoacyl-[acyl-carrier-protein] synthase I, chloroplastic-like
Pp3c7_2800	2.78	4.89e-34	protein WVD2-like 4
Pp3c9_3139	2.78	8.50e-13	U-box domain-containing protein 44-like
Pp3c24_2393	2.78	2.23e-36	uncharacterized protein LOC112291717
Pp3c1_25080	2.77	3.29e-38	cell division control protein 45 homolog
Pp3c18_22150	2.77	1.63e-24	hypothetical protein PHYPA_004108
Pp3c12_14478	2.77	5.53e-10	hypothetical protein PHYPA_010791
Pp3c23_1080	2.77	2.67e-18	spastin isoform X1
Pp3c22_14940	2.76	4.27e-12	hypothetical protein PHYPA_008068
Pp3c1_12220	2.76	4.20e-15	putative MADS-domain transcription factor
Pp3c12_20160	2.76	2.19e-99	uncharacterized protein LOC112285023
Pp3c27_6880	2.75	1.93e-41	conserved oligomeric Golgi complex subunit 4-like
Pp3c18_17790	2.75	1.19e-59	dolichol kinase EVAN-like
Pp3c21_14670	2.75	9.07e-14	hypothetical protein PHYPA_030830
Pp3c6_17860	2.75	1.88e-15	non-specific lipid-transfer protein 4-like
Pp3c4_8790	2.74	3.82e-03	restin homolog isoform X2
Pp3c2_37560	2.74	2.45e-09	translation initiation factor fusion with methylthioribose kinase
Pp3c13_3830	2.73	1.86e-31	2,3-bisphosphoglycerate-dependent phosphoglycerate mutase
Pp3c18_22140	2.73	2.39e-09	DNA-directed RNA polymerase III subunit RPC2
Pp3c13_17100	2.73	5.34e-55	hypothetical protein PHYPA_020146
Pp3c8_12680	2.73	1.99e-44	WUSCHEL-related homeobox 8-like
Pp3c1_41530	2.72	1.79e-20	S-adenosyl-L-methionine-dependent methyltransferases superfamily protein
Pp3c1_36360	2.72	8.35e-43	sucrose transport protein SUC3-like
Pp3c23_20910	2.71	6.33e-81	3-deoxy-manno-octulosonate cytidylyltransferase, mitochondrial-like
Pp3c10_12760	2.71	8.18e-31	aminodeoxychorismate synthase, chloroplastic isoform X1
Pp3c3_17950	2.71	7.05e-04	hypothetical protein PHYPA_029854

Pp3c6_10250	2.71	1.99e-11	Localized to the inner membrane of the chloroplast
Pp3c11_14310	2.71	7.20e-16	predicted protein
Pp3c11_14770	2.70	2.82e-81	glycosyltransferase family 92 protein RCOM_0530710-like
Pp3c17_14090	2.70	3.18e-34	hypothetical protein PHYPA_004362
Pp3c1_2700	2.70	9.41e-15	light-sensor Protein kinase-like
Pp3c2_2790	2.70	6.10e-14	mitotic-spindle organizing protein 1-like
Pp3c5_23340	2.70	1.21e-69	TLD domain-containing protein 1
Pp3c7_25640	2.69	3.63e-54	DUF21 domain-containing protein At4g14240-like
Pp3c1_470	2.69	4.41e-07	hypothetical protein PHYPA_000194
Pp3c3_22320	2.69	2.26e-48	hypothetical protein PHYPA_029533
Pp3c5_3340	2.69	3.72e-76	kinesin-like protein KIF16B isoform X1
Pp3c2_1951	2.69	3.55e-22	leucine-rich repeat extensin-like protein 5
Pp3c15_6210	2.69	1.99e-20	putative retrotransposon gag domain, Retroviral aspartyl protease
Pp3c1_33540	2.69	1.83e-14	pyruvate decarboxylase 1
Pp3c4_17130	2.69	7.61e-11	senescence-associated protein AAF, chlorolplastic-like
Pp3c24_11130	2.69	2.92e-31	uncharacterized protein LOC112289464
Pp3c7_26390	2.67	2.66e-34	hypothetical protein PHYPA_028591
Pp3c1_39250	2.67	1.16e-53	phosphate acetyltransferase
Pp3c1_2960	2.67	1.84e-19	PREDICTED: uncharacterized protein LOC107808225
Pp3c15_12420	2.66	1.43e-11	BRO1 domain containing protein
Pp3c1_35228	2.66	3.05e-19	hypothetical protein PHYPA_001383, partial
Pp3c3_1210	2.66	5.47e-63	hypothetical protein PHYPA_008552
Pp3c5_13590	2.66	1.55e-29	hypothetical protein PHYPA_014164
Pp3c7_13610	2.66	6.45e-30	hypothetical protein PHYPA_021817
Pp3c21_16610	2.66	7.32e-18	hypothetical protein PHYPA_030722
Pp3c10_8620	2.66	2.27e-12	NADH:ubiquinone oxidoreductase
Pp3c24_14460	2.66	5.44e-27	peptidyl-prolyl cis-trans isomerase CYP28, chloroplastic
Pp3c13_23010	2.66	2.36e-10	predicted protein
Pp3c1_35940	2.66	2.19e-99	thioredoxin-like protein Clot
Pp3c6_26980	2.65	3.07e-09	aldehyde oxidase GLOX-like
Pp3c10_7650	2.65	2.43e-13	cilia- and flagella-associated protein 44-like isoform X1
Pp3c16_23960	2.65	3.13e-09	E3 ubiquitin-protein ligase BRE1-like 1 isoform X1
Pp3c6_15470	2.65	2.67e-10	hypothetical protein PHYPA_026733
Pp3c10_8570	2.65	7.42e-64	malate dehydrogenase, mitochondrial
Pp3c15_12250	2.65	8.04e-09	pentatricopeptide repeat-containing protein At2g41720
Pp3c5_11050	2.64	2.50e-09	26S proteasome non-ATPase regulatory subunit 14 homolog
Pp3c1_2770	2.64	1.33e-15	hypothetical protein PHYPA_011998

Pp3c19_9850	2.64	1.34e-28	---NA---
Pp3c17_13270	2.64	5.73e-47	Nramp-domain-containing protein
Pp3c21_5600	2.64	1.93e-16	Retrovirus-related Pol polyprotein from transposon TNT 1-94
Pp3s37_40	2.64	9.29e-09	uncharacterized protein LOC112277321
Pp3c12_19130	2.63	1.05e-13	digalactosyldiacylglycerol synthase 2, chloroplastic-like
Pp3c1_39210	2.63	1.23e-52	NADP-specific glutamate dehydrogenase isoform X1
Pp3c14_3405	2.63	1.79e-25	probable cinnamyl alcohol dehydrogenase 1
Pp3c1_2880	2.63	5.21e-09	prohibitin-3, mitochondrial-like
Pp3c18_22071	2.63	4.28e-08	ubiquitin domain-containing protein 1-like isoform X2
Pp3c10_1200	2.62	8.05e-20	GDSL esterase/lipase At1g09390-like isoform X1
Pp3c1_31630	2.62	8.53e-22	hypothetical protein PHYPA_000618
Pp3c1_21920	2.62	2.64e-06	hypothetical protein PHYPA_011635
Pp3c13_21450	2.62	4.61e-44	methyl-CpG-binding domain protein 4-like protein
Pp3c13_12610	2.61	5.89e-56	histone acetyltransferase type B catalytic subunit isoform X2
Pp3c8_14030	2.61	2.02e-39	hypothetical protein PHYPA_012734
Pp3c8_19850	2.61	3.12e-21	phosphoglycerate mutase-like protein
Pp3c18_10070	2.61	3.30e-14	protein-L-isoaspartate O-methyltransferase 1-like
Pp3c14_12730	2.61	4.37e-08	ribulose bisphosphate carboxylase small chain clone 512-like
Pp3c1_2920	2.60	3.87e-09	hypothetical protein PHYPA_000054
Pp3c5_4780	2.60	4.39e-07	inactive protein kinase SELMODRAFT_444075-like
Pp3c9_1620	2.60	4.50e-45	outer envelope protein 64, mitochondrial
Pp3c7_16840	2.60	3.42e-13	ribosomal RNA small subunit methyltransferase NEP1
Pp3c25_950	2.59	9.95e-54	serine/arginine repetitive matrix protein 2 isoform X1
Pp3c17_5790	2.58	5.33e-50	hypothetical protein PHYPA_004981
Pp3c14_3280	2.58	1.39e-06	putative LOV domain-containing protein
Pp3c2_11350	2.58	2.49e-08	Retrovirus-related Pol polyprotein from transposon TNT 1-94
Pp3c20_16830	2.57	5.40e-18	EPIDERMAL PATTERNING FACTOR-like protein 4
Pp3c21_20300	2.57	2.33e-25	glutamyl-tRNA(Gln) amidotransferase subunit A
Pp3c20_5860	2.57	2.68e-49	hornerin-like isoform X2
Pp3c1_16910	2.57	1.12e-79	hypothetical protein PHYPA_018185
Pp3c20_10050	2.57	9.72e-44	---NA---
Pp3c19_2720	2.57	6.00e-07	probable ATP-dependent DNA helicase CHR12

Pp3c2_34820	2.57	5.17e-44	probable polyamine transporter At3g19553
Pp3c17_18550	2.57	1.97e-14	ultraviolet-B receptor UVR8
Pp3c2_19640	2.57	1.14e-15	uncharacterized protein LOC112290067
Pp3c16_22670	2.56	6.51e-24	cold-responsive protein kinase 1-like
Pp3c16_14730	2.56	1.18e-06	peptide methionine sulfoxide reductase A5
Pp3c4_31920	2.56	9.66e-12	transketolase, chloroplastic
Pp3c16_23100	2.55	6.15e-03	calcium-dependent protein kinase
Pp3c1_2650	2.55	2.10e-08	desiccation-related protein At2g46140
Pp3c14_21347	2.55	2.07e-07	light-sensor Protein kinase-like
Pp3c27_6210	2.55	1.84e-12	probable mannitol dehydrogenase
Pp3c1_35610	2.55	3.41e-29	retrotransposon reverse transcriptase
Pp3c15_4170	2.55	2.12e-12	uncharacterized protein LOC112278766 isoform X1
Pp3c14_1980	2.54	1.95e-32	histone H2B.8-like
Pp3c19_20620	2.54	3.48e-09	hypothetical protein PHYPA_024465
Pp3c18_15920	2.54	1.42e-35	protein AUXIN SIGNALING F-BOX 3-like
Pp3c21_11360	2.54	3.82e-53	SPX domain-containing protein 1-like
Pp3c8_19160	2.54	2.59e-44	topless-related protein 1-like isoform X1
Pp3c11_8850	2.54	2.33e-38	transmembrane protein 45A-like
Pp3c3_20980	2.53	4.18e-72	cytochrome b-c1 complex subunit 7-2-like
Pp3c10_11042	2.53	2.40e-10	gibberellin 3-beta-dioxygenase 1-like
Pp3c1_5000	2.53	1.18e-26	hypothetical protein PHYPA_000030
Pp3c9_19810	2.53	1.33e-26	mono [ADP-ribose] polymerase PARP16
Pp3s1097_10	2.53	3.84e-07	predicted protein
Pp3c5_5250	2.52	7.89e-13	probable elongation factor 1-gamma 2
Pp3c7_15720	2.52	8.37e-30	uncharacterized protein LOC112276660 isoform X2
Pp3c10_7990	2.51	1.70e-08	DExH-box ATP-dependent RNA helicase DExH15 chloroplastic
Pp3c2_18850	2.51	1.46e-22	homeobox-leucine zipper protein HOX20-like isoform X1
Pp3c16_14660	2.51	4.63e-06	hypothetical protein PHYPA_023511
Pp3c4_2660	2.51	1.22e-02	kelch domain-containing protein 4
Pp3c16_14590	2.51	3.95e-06	---NA---
Pp3c1_36490	2.51	2.33e-32	NADH dehydrogenase [ubiquinone] iron-sulfur protein 1, mitochondrial
Pp3c6_14750	2.51	2.71e-21	ribonucleoside-diphosphate reductase large subunit-like
Pp3c5_15400	2.51	6.15e-35	RING/FYVE/PHD zinc finger superfamily protein, putative isoform 2
Pp3c10_3550	2.51	1.53e-09	transcription factor ILR3-like
Pp3c1_36300	2.51	1.32e-35	uncharacterized protein LOC112292081 isoform X2
Pp3c2_6500	2.50	1.07e-15	hypothetical protein PHYPA_009447
Pp3c16_14640	2.50	5.93e-06	hypothetical protein PHYPA_019603
Pp3c20_5150	2.49	4.04e-42	hypothetical protein PHYPA_027552

Pp3c5_24760	2.49	4.09e-66	receptor homology region, transmembrane domain- and RING domain-containing protein 1-like isoform X1
Pp3c17_9830	2.49	4.36e-05	subtilisin-like protease SBT6.1
Pp3c4_20080	2.48	3.21e-12	coiled-coil domain-containing protein 18-like
Pp3c20_7230	2.48	3.02e-09	DNA replication licensing factor MCM6
Pp3c26_8990	2.48	6.75e-20	hypothetical protein PHYPA_001202
Pp3c21_18700	2.48	1.47e-35	root phototropism protein 3-like
Pp3c14_8980	2.47	1.24e-11	2-Cys peroxiredoxin BAS1, chloroplastic-like
Pp3c1_17450	2.47	3.73e-06	histone-lysine N-methyltransferase 2D-like isoform X1
Pp3c20_18130	2.47	2.11e-50	probable pre-mRNA-splicing factor ATP-dependent RNA helicase DEAH2 isoform X1
Pp3c24_4040	2.46	1.28e-08	30S ribosomal protein S A
Pp3c13_8100	2.45	2.74e-21	actin cytoskeleton-regulatory complex protein pan-1
Pp3c1_2520	2.45	8.97e-13	predicted protein
Pp3c14_12090	2.45	3.13e-09	protein COFACTOR ASSEMBLY OF COMPLEX C SUBUNIT B CCB2, chloroplastic-like
Pp3c23_15420	2.44	1.51e-03	Histone-lysine N-methyltransferase ATXR2
Pp3c20_14530	2.44	2.19e-06	hypothetical protein OsI_24495
Pp3c1_14250	2.44	3.42e-10	---NA---
Pp3c13_23030	2.44	1.35e-08	pfkB family carbohydrate kinase
Pp3c20_15000	2.44	2.08e-09	probable cinnamyl alcohol dehydrogenase 6
Pp3c11_12290	2.44	1.17e-47	protein saal1-like isoform X4
Pp3c7_22860	2.44	3.99e-11	uncharacterized protein LOC112277036
Pp3c16_17510	2.43	1.23e-26	chalcone synthase-like
Pp3c15_11920	2.43	5.24e-17	hypothetical protein PHYPA_002915
Pp3c13_22390	2.43	8.99e-34	nuclear transcription factor Y subunit B-3-like
Pp3c12_20050	2.42	3.26e-22	mitogen-activated protein kinase kinase kinase 1-like
Pp3c11_17210	2.42	1.87e-13	---NA---
Pp3c3_18870	2.42	7.30e-07	transmembrane protein 128
Pp3c18_21810	2.41	6.22e-32	heavy metal-associated isoprenylated plant protein 30-like
Pp3c25_11400	2.41	1.05e-21	hexokinase-1-like
Pp3c19_8700	2.41	1.39e-37	hypothetical protein VOLCADRAFT_95342
Pp3c21_13220	2.41	1.39e-12	pentatricopeptide repeat-containing-like protein
Pp3c8_20410	2.41	4.65e-07	putative Na P-type ATPase
Pp3c1_42570	2.41	4.49e-11	tetratricopeptide repeat protein 28-like
Pp3c24_20470	2.40	1.67e-08	hydroxyproline O-arabinosyltransferase 3-like

Pp3c5_3040	2.40	3.18e-31	hypothetical protein PHYPA_027068, partial
Pp3c6_3530	2.40	8.96e-38	nucleolar and coiled-body phosphoprotein 1-like
Pp3c3_31700	2.40	3.76e-26	protein MIZU-KUSSEI 1
Pp3c17_16260	2.40	3.04e-111	uncharacterized protein LOC112279939
Pp3c10_8200	2.39	8.51e-28	ADP-ribosylation factor 1-like
Pp3c23_12620	2.39	9.27e-33	apolipoprotein D-like
Pp3c26_12710	2.39	6.02e-13	calcium-transporting ATPase 8, plasma membrane-type-like
Pp3c14_24300	2.39	2.10e-18	E3 ubiquitin-protein ligase SPL2
Pp3c12_25770	2.39	2.99e-05	E3 ubiquitin-protein ligase UPL1-like isoform X1
Pp3c12_24670	2.39	2.42e-52	hypothetical protein PHYPA_010066
Pp3c1_2830	2.39	2.89e-20	proteasome subunit alpha type-6
Pp3c20_2485	2.39	4.65e-08	protein piccolo-like
Pp3c20_200	2.38	1.15e-27	callose synthase 12-like
Pp3c26_2640	2.38	3.17e-08	hypothetical protein PHYPA_000845
Pp3c27_8130	2.37	1.90e-67	cellulose synthase-like protein D3
Pp3c12_21260	2.37	1.23e-12	hypothetical protein PHYPA_009922
Pp3c14_17020	2.37	6.44e-90	hypothetical protein PHYPA_016864
Pp3c10_13010	2.36	3.09e-08	aldehyde oxidase GLOX-like
Pp3c20_3900	2.36	5.81e-07	BTB/POZ domain-containing protein At3g05675-like
Pp3c16_9250	2.36	3.29e-24	putative phospholipid-transporting ATPase 9 isoform X1
Pp3c6_28370	2.36	2.62e-19	tetratricopeptide repeat protein 38
Pp3c20_6780	2.36	1.98e-22	uncharacterized PKHD-type hydroxylase At1g22950-like
Pp3c11_24860	2.36	1.83e-09	uncharacterized protein LOC112280777 isoform X1
Pp3c17_15650	2.35	9.92e-80	40S ribosomal protein S17-like
Pp3c14_12710	2.35	1.03e-04	FACT complex subunit SPT16-like
Pp3c13_22780	2.35	2.11e-16	hypothetical protein PHYPA_019357
Pp3c18_16800	2.35	1.19e-14	nuclear pore complex protein NUP155
Pp3c2_6160	2.35	2.59e-17	phosphomevalonate kinase, peroxisomal-like
Pp3c8_14810	2.35	1.12e-18	polyphenol oxidase, chloroplastic-like
Pp3c17_6080	2.34	9.89e-23	chloroplast outer envelope 24 kD protein (omp24)
Pp3c4_11080	2.34	3.15e-24	hypothetical protein PHYPA_022924
Pp3c26_2630	2.34	9.92e-08	importin subunit alpha-1-like isoform X1
Pp3c4_29300	2.34	2.31e-30	---NA---
Pp3c21_10100	2.34	1.64e-05	Retrovirus-related Pol polyprotein from transposon TNT 1-94
Pp3c10_25590	2.34	3.59e-23	soul heme-binding family protein
Pp3c8_19590	2.33	2.10e-11	50S ribosomal protein L11, chloroplastic-like
Pp3c18_8540	2.33	1.52e-06	hypothetical protein PHYPA_025077

Pp3c3_25940	2.33	5.52e-22	hypothetical protein PHYPA_029700
Pp3c20_17310	2.33	9.99e-21	mitochondrial import inner membrane translocase subunit TIM22-2-like
Pp3c27_6610	2.33	4.05e-06	predicted protein
Pp3c22_20140	2.33	3.36e-07	Zinc finger, PHD-type
Pp3c26_14690	2.32	6.11e-23	40S ribosomal protein S27-2
Pp3c20_15090	2.32	2.03e-17	---NA---
Pp3c5_11730	2.32	8.28e-21	Phox homologous domain
Pp3c17_18740	2.32	3.37e-14	predicted protein
Pp3c20_9341	2.31	2.97e-16	axoneme-associated protein MST101(2) protein
Pp3c1_40420	2.31	5.17e-05	clathrin heavy chain 1
Pp3c14_5450	2.31	3.13e-12	hypothetical protein PHYPA_001981
Pp3c15_5630	2.31	2.11e-30	hypothetical protein PHYPA_003657
Pp3c5_24940	2.31	1.69e-44	multifunctional methyltransferase subunit TRM112-like protein At1g22270
Pp3c3_23910	2.31	6.23e-42	transcription factor FAMA-like isoform X1
Pp3c1_42450	2.30	2.90e-28	DNA-3-methyladenine glycosylase
Pp3c3_13510	2.30	7.13e-30	metalloendoproteinase 1-MMP-like
Pp3c2_28330	2.30	3.88e-06	probable RNA helicase SDE3
Pp3c9_570	2.30	2.60e-25	protein fluG-like
Pp3c21_15300	2.30	4.12e-20	Retrovirus-related Pol polyprotein from transposon TNT 1-94
Pp3c11_7750	2.30	1.73e-05	zinc finger protein 706-like
Pp3c7_25050	2.29	5.51e-05	cyclin-dependent protein kinase inhibitor SMR9-like
Pp3c20_20430	2.29	3.16e-28	DTW domain-containing protein 2-like
Pp3c1_2980	2.29	2.46e-12	hypothetical protein PHYPA_000533
Pp3c17_7780	2.29	1.22e-04	hypothetical protein PHYPA_005070
Pp3c8_15710	2.29	1.99e-10	hypothetical protein PHYPA_012812
Pp3c5_19530	2.29	1.03e-39	hypothetical protein PHYPA_013348
Pp3c5_14090	2.28	4.20e-08	callose synthase 9-like isoform X1
Pp3c6_28490	2.28	2.19e-22	centrosome-associated protein CEP250-like isoform X1
Pp3c7_12700	2.28	1.28e-39	coronatine-insensitive protein 1
Pp3c5_24100	2.28	1.05e-25	hypothetical protein PHYPA_013565
Pp3c8_1520	2.28	9.42e-05	phosphatidylinositol 4-kinase gamma 3
Pp3c2_18570	2.28	2.23e-09	stromal cell-derived factor 2-like protein
Pp3c8_22560	2.28	2.80e-17	TMV resistance protein N-like
Pp3c2_1940	2.27	9.55e-13	carotenoid 9,10(9',10')-cleavage dioxygenase 1-like
Pp3c13_21340	2.27	2.53e-02	hypothetical protein PHYPA_019288
Pp3c19_22400	2.27	8.85e-11	hypothetical protein PHYPA_024661
Pp3c3_37730	2.27	2.26e-10	long chain acyl-CoA synthetase 6, peroxisomal-like isoform X5
Pp3c22_20240	2.27	4.00e-07	probable plastidic glucose transporter 2 isoform X1
Pp3c3_15040	2.27	4.96e-08	transcription factor bHLH84

Pp3c15_12390	2.26	2.24e-07	hypothetical protein PHYPA_002066
Pp3c7_18490	2.26	1.28e-05	hypothetical protein PHYPA_029016
Pp3c10_10710	2.26	7.80e-18	hypothetical protein PHYPA_030630
Pp3c23_7740	2.26	6.52e-43	mitochondrial uncoupling protein 5-like
Pp3c25_1860	2.26	1.25e-08	MLP-like protein 423
Pp3c19_5710	2.26	1.10e-13	---NA---
Pp3c15_7040	2.26	1.15e-16	uncharacterized protein LOC112295054
Pp3c7_20870	2.25	8.52e-16	50S ribosomal protein L25-like
Pp3c5_24720	2.25	1.81e-16	60S ribosomal protein L31
Pp3c26_12810	2.25	2.37e-26	cytochrome P450 71A1-like
Pp3c15_20060	2.25	5.96e-08	dof zinc finger protein DOF4.3-like
Pp3c14_6070	2.25	1.20e-81	hypothetical protein PHYPA_002628
Pp3c7_7810	2.25	7.49e-17	---NA---
Pp3c21_3760	2.25	2.43e-40	probable pectinesterase 53
Pp3c26_8420	2.25	1.13e-07	Testis-expressed sequence 2 protein
Pp3c20_6660	2.24	1.11e-08	hypothetical protein PHYPA_027570
Pp3c10_17080	2.24	5.04e-14	hypothetical protein PHYPA_030053
Pp3c18_11190	2.24	1.81e-16	squamosa promoter-binding-like protein 7 isoform X1
Pp3c9_24030	2.23	3.49e-05	biogenesis of lysosome-related organelles complex 1 subunit 2
Pp3c21_1310	2.23	1.84e-08	ethylene-responsive transcription factor 3-like
Pp3c23_5960	2.23	9.09e-15	protein HASTY 1
Pp3c1_28510	2.23	6.99e-08	sugar transporter ERD6-like 4
Pp3c12_12080	2.22	6.63e-25	dihydrolipoyllysine-residue succinyltransferase component of 2-oxoglutarate dehydrogenase complex 2, mitochondrial-like isoform X4
Pp3c18_1710	2.22	1.83e-16	hypothetical protein PHYPA_005465
Pp3c11_22850	2.22	4.12e-14	hypothetical protein PHYPA_005861
Pp3c10_6710	2.22	4.59e-23	hypothetical protein PHYPA_006436
Pp3c21_19440	2.22	1.81e-04	---NA---
Pp3c20_3140	2.22	5.63e-06	protein SUPPRESSOR OF npr1-1, CONSTITUTIVE 1-like
Pp3c17_13150	2.22	2.87e-06	uncharacterized protein LOC112279533
Pp3c4_10820	2.22	1.64e-05	uncharacterized protein LOC112295118
Pp3c17_21590	2.21	1.03e-10	60S ribosomal protein L23A
Pp3c26_8290	2.21	1.10e-14	ACT domain-containing protein ACR9-like isoform X2
Pp3c7_23740	2.21	6.06e-26	bifunctional nuclease 1-like
Pp3c25_5790	2.21	2.96e-06	E3 UFM1-protein ligase 1 homolog
Pp3c11_2870	2.21	3.18e-11	glycosyltransferase family 64 protein C4
Pp3c22_15560	2.21	1.64e-14	hypothetical protein PHYPA_008089
Pp3c12_4260	2.21	1.00e-41	hypothetical protein PHYPA_019570
Pp3c22_8970	2.21	2.99e-06	non-specific lipid-transfer protein-like protein At2g13820

Pp3c2_30580	2.20	2.74e-38	50S ribosomal protein L28, chloroplastic-like
Pp3c12_5140	2.20	1.43e-07	ARF guanine-nucleotide exchange factor GNOM
Pp3c14_20010	2.20	1.22e-11	hypothetical protein PHYPA_017769
Pp3c7_430	2.20	6.12e-07	nuclear pore complex protein NUP58
Pp3c10_8000	2.20	1.00e-25	protein-L-isoaspartate O-methyltransferase 1-like
Pp3c15_22980	2.20	6.10e-17	uncharacterized protein LOC112295735
Pp3c5_18730	2.19	4.20e-27	GDSL esterase/lipase At3g26430
Pp3c19_14040	2.19	4.06e-37	hypothetical protein PHYPA_025278
Pp3c9_2080	2.19	4.01e-08	peptide methionine sulfoxide reductase A5-like isoform X1
Pp3c16_16710	2.19	1.69e-87	probable xyloglucan 6-xylosyltransferase 5
Pp3c4_20210	2.19	6.26e-70	protein SUPPRESSOR OF npr1-1, CONSTITUTIVE 1-like isoform X1
Pp3c16_25090	2.19	4.08e-05	ribosomal protein S19 (chloroplast)
Pp3c15_18090	2.18	5.31e-28	hypothetical protein PHYPA_003162
Pp3c13_2080	2.18	4.23e-06	hypothetical protein PHYPA_019263
Pp3c9_21410	2.18	3.35e-15	hypothetical protein PHYPA_026491
Pp3c14_24040	2.18	5.44e-07	oxygen-evolving enhancer protein 3-1, chloroplastic-like
Pp3c19_2670	2.18	2.96e-05	phenylalanine ammonia-lyase-like
Pp3c1_12230	2.18	8.04e-09	putative MADS-domain transcription factor
Pp3c11_5710	2.17	2.51e-56	5-amino-6-(5-phospho-D-ribitylamino)uracil phosphatase, chloroplastic-like
Pp3c1_9560	2.17	3.17e-19	hypothetical protein PHYPA_016097
Pp3c23_11240	2.17	2.78e-17	probable histone-arginine methyltransferase CARM1
Pp3c18_17810	2.17	2.89e-16	pumilio homolog 1-like
Pp3c9_4620	2.17	6.04e-07	uncharacterized protein LOC112274085
Pp3c9_16610	2.16	1.70e-12	60S acidic ribosomal protein P3-2
Pp3c21_6390	2.16	3.52e-19	casein kinase II subunit beta-3-like isoform X1
Pp3c18_15890	2.16	5.25e-33	hypothetical protein PHYPA_005419
Pp3c22_8950	2.16	8.15e-50	hypothetical protein PHYPA_014904
Pp3c13_8200	2.16	2.51e-11	hypothetical protein PHYPA_017245
Pp3c16_3330	2.16	3.92e-08	methyltransferase-like protein 13
Pp3c25_10450	2.16	1.19e-67	MLO-like protein 5
Pp3c23_5270	2.16	2.35e-05	---NA---
Pp3c20_14770	2.15	3.45e-14	cationic amino acid transporter 6, chloroplastic-like
Pp3c11_3960	2.15	6.78e-29	chaperone protein dnaJ 15-like isoform X1
Pp3c4_24710	2.15	8.68e-07	glucose-6-phosphate isomerase 1, chloroplastic
Pp3c27_4280	2.15	4.13e-22	hypothetical protein PHYPA_001484
Pp3c2_21330	2.15	2.06e-42	probable splicing factor, arginine/serine-rich 4

Pp3c8_12410	2.15	2.31e-72	R3H domain-containing protein 2-like
Pp3c10_3790	2.14	6.01e-26	Alpha-protein kinase vwkA
Pp3c26_11700	2.14	4.70e-20	hypothetical protein PHYPA_011309
Pp3c1_24700	2.14	4.14e-03	hypothetical protein PHYPA_011871, partial
Pp3c5_24680	2.14	2.72e-12	hypothetical protein PHYPA_013590
Pp3c3_20420	2.14	1.63e-40	hypothetical protein PHYPA_029965
Pp3c9_5080	2.14	1.46e-03	LRR receptor-like serine/threonine-protein kinase ERL1
Pp3c4_29120	2.14	3.03e-18	transcription factor bHLH49-like
Pp3c9_13730	2.14	9.65e-16	translation initiation factor eIF-2B subunit beta-like
Pp3c11_760	2.14	1.10e-20	uncharacterized protein LOC112285205 isoform X1
Pp3c2_25070	2.14	2.41e-04	zinc finger protein squeeze isoform X2
Pp3c9_19210	2.13	6.00e-25	galactan beta-1,4-galactosyltransferase GALS3-like
Pp3c18_18540	2.13	4.52e-26	hypothetical protein PHYPA_005513
Pp3c22_21290	2.13	4.02e-07	hypothetical protein PHYPA_008299
Pp3c10_8020	2.13	7.26e-41	NAC domain-containing protein 76-like
Pp3c21_14500	2.13	7.90e-46	tobamovirus multiplication protein 2A-like
Pp3c21_440	2.12	2.95e-06	F-box/kelch-repeat protein At5g15710-like
Pp3c4_22330	2.12	3.33e-24	hypothetical protein PHYPA_021421
Pp3c1_24600	2.12	1.29e-02	NADPH--cytochrome P450 reductase-like
Pp3c16_24050	2.12	1.96e-07	palmitoyl-acyl carrier protein thioesterase, chloroplastic-like
Pp3c8_15360	2.12	3.48e-15	predicted protein
Pp3c7_13080	2.12	1.97e-47	short-chain dehydrogenase TIC 32, chloroplastic
Pp3c2_32020	2.12	3.36e-12	testis-expressed sequence 2 protein
Pp3c6_6545	2.11	5.57e-34	cellulose synthase-like protein D3
Pp3c6_7260	2.11	1.32e-45	disease resistance protein TAO1-like
Pp3c10_21660	2.11	1.72e-05	hypothetical protein PHYPA_006069
Pp3c22_9051	2.11	1.58e-37	kinetochore protein NDC80 homolog
Pp3c22_9050	2.11	2.02e-37	transportin MOS14 isoform X1
Pp3c1_14870	2.10	3.84e-18	DNA polymerase
Pp3c16_13060	2.10	1.16e-12	hypothetical protein PHYPA_002390
Pp3c25_12800	2.10	5.39e-14	hypothetical protein PHYPA_018179
Pp3c16_2040	2.10	4.78e-11	RNA polymerase-associated protein LEO1-like
Pp3c2_6210	2.10	9.90e-13	syntaxin-81
Pp3c13_5660	2.09	9.25e-24	ATP-cone
Pp3c16_13490	2.09	3.44e-30	protein phosphatase 1 regulatory subunit pprA
Pp3c6_13600	2.09	4.43e-07	Trafficking protein particle complex subunit 5
Pp3c4_27280	2.09	8.22e-26	tRNA dimethylallyltransferase 9-like isoform X1

Pp3c23_16520	2.09	1.70e-04	uncharacterized membrane protein At1g06890
Pp3c18_20200	2.08	2.37e-26	ADP-ribosylation factor-like protein 5
Pp3c16_13280	2.08	2.28e-04	hypothetical protein PHYPA_002405
Pp3c21_9570	2.08	5.15e-17	hypothetical protein PHYPA_007837
Pp3c10_8950	2.08	1.35e-05	WAT1-related protein At4g19185-like
Pp3c1_36390	2.07	2.78e-18	ABC transporter B family member 25, mitochondrial-like
Pp3c23_1100	2.07	2.85e-12	DNA polymerase delta catalytic subunit-like
Pp3c18_4930	2.07	1.26e-13	hypothetical protein AXX17_ATUG04580
Pp3c26_8080	2.07	3.10e-34	hypothetical protein PHYPA_000101
Pp3c6_13660	2.07	6.78e-08	pentatricopeptide repeat-containing protein At5g02860
Pp3c22_16800	2.07	9.36e-05	UDP-glucose 4-epimerase GEPI48-like
Pp3c19_10140	2.07	2.81e-27	uncharacterized protein LOC112272993
Pp3c15_5060	2.06	1.79e-09	Aquaporin NIP6-1
Pp3c17_10600	2.06	7.28e-06	coiled-coil domain-containing protein SCD2-like
Pp3c4_22010	2.06	1.76e-04	exocyst complex component SEC8-like isoform X1
Pp3c2_32690	2.06	2.90e-46	hypothetical protein PHYPA_008371, partial
Pp3c14_20340	2.06	2.35e-12	hypothetical protein PHYPA_017784
Pp3c3_6480	2.06	6.70e-42	hypothetical protein PHYPA_022745
Pp3c22_10280	2.06	1.99e-07	putative ABC1 family protein
Pp3c24_14851	2.06	5.80e-04	putative chaperone protein HSP31
Pp3c11_1560	2.06	3.88e-23	Retrovirus-related Pol polyprotein from transposon TNT 1-94
Pp3c3_6890	2.06	2.17e-05	Retrovirus-related Pol polyprotein from transposon TNT 1-94
Pp3c4_11030	2.06	1.20e-30	Retrovirus-related Pol polyprotein from transposon TNT 1-94
Pp3c23_19470	2.06	8.35e-31	root phototropism protein 3-like isoform X1
Pp3c13_4250	2.06	4.40e-46	THO complex subunit 3
Pp3c6_7500	2.06	1.62e-09	uncharacterized protein LOC112294206 isoform X1
Pp3c16_880	2.05	3.24e-22	20 kDa chaperonin, chloroplastic-like
Pp3c27_6870	2.05	2.69e-25	conserved oligomeric Golgi complex subunit 4-like
Pp3c26_5390	2.05	1.14e-06	hypothetical protein PHYPA_000974
Pp3c10_22510	2.05	1.63e-05	hypothetical protein PHYPA_006105
Pp3c5_8550	2.05	1.28e-11	PITH domain-containing protein 1
Pp3c23_2252	2.05	1.41e-05	putative lipid-transfer protein DIR1
Pp3c10_6930	2.05	2.91e-11	tRNA (mo5U34)-methyltransferase
Pp3c1_30760	2.04	5.56e-28	DDB1- and CUL4-associated factor homolog 1
Pp3c9_13290	2.04	4.30e-34	enhancer of mRNA-decapping protein 4-like isoform X1

Pp3c25_330	2.04	1.20e-31	hypothetical protein PHYPA_011541
Pp3c11_9619	2.04	3.40e-04	kinesin-like protein KIN-12B
Pp3c6_1880	2.04	6.42e-18	MLO-like protein 14
Pp3c11_9670	2.04	9.97e-23	potassium channel GORK-like isoform X1
Pp3c23_16640	2.04	5.76e-10	predicted protein
Pp3c15_2600	2.04	4.34e-05	transcriptional corepressor LEUNIG isoform X1
Pp3c14_1030	2.03	2.69e-04	ATP-dependent DNA helicase PIF1-like
Pp3c22_22340	2.03	2.69e-10	glucose-6-phosphate isomerase, cytosolic
Pp3c18_20910	2.03	2.70e-18	NADH-ubiquinone oxidoreductase 11 kDa subunit
Pp3c4_1560	2.03	5.19e-18	protein ZINC INDUCED FACILITATOR-LIKE 1-like isoform X4
Pp3c14_12830	2.03	3.21e-12	ribulose bisphosphate carboxylase small chain clone 512-like
Pp3c7_229	2.03	6.38e-05	RNA polymerase II-associated protein 3 isoform X1
Pp3c6_5280	2.03	4.06e-35	TMV resistance protein N-like
Pp3c24_690	2.02	7.06e-40	3-hydroxyisobutyryl-CoA hydrolase-like protein 3, mitochondrial isoform X1
Pp3c2_27810	2.02	1.04e-16	dnaJ protein ERDJ3B-like
Pp3c16_9080	2.02	7.77e-05	glucose-1-phosphate adenylyltransferase large subunit 1
Pp3c19_18560	2.02	2.62e-03	glucose-6-phosphate/phosphate translocator 1, chloroplastic-like
Pp3c14_4830	2.02	2.94e-22	heat shock 70 kDa protein, mitochondrial
Pp3c13_20650	2.02	3.73e-12	hypothetical protein PHYPA_019112
Pp3c27_4780	2.02	7.78e-03	predicted protein
Pp3c17_5820	2.02	9.56e-11	proton pump-interactor 1-like
Pp3c6_25090	2.02	1.36e-15	putative ion channel POLLUX-like 2 isoform X1
Pp3c3_23850	2.02	2.74e-45	two-component response regulator-like PRR37 isoform X1
Pp3c22_16380	2.02	3.03e-33	vegetative cell wall protein gp1-like
Pp3c2_28350	2.01	2.66e-05	AP-1 complex subunit gamma-2 isoform X1
Pp3c23_14750	2.01	4.81e-15	box C/D snoRNA protein 1
Pp3c6_2960	2.01	2.58e-07	DNA polymerase delta catalytic subunit
Pp3c14_13540	2.01	1.25e-03	hypothetical protein PHYPA_017491
Pp3c15_17070	2.01	1.17e-03	magnesium transporter MRS2-3
Pp3c3_27940	2.00	1.29e-16	4-coumarate--CoA ligase-like 5
Pp3c7_17990	2.00	1.04e-20	50S ribosomal protein L19-1, chloroplastic
Pp3c2_30060	2.00	8.46e-17	endonuclease V isoform X1
Pp3c26_10501	2.00	1.13e-06	hypothetical protein PHYPA_011238
Pp3c8_15610	2.00	2.41e-22	hypothetical protein PHYPA_012807
Pp3c4_27650	2.00	7.95e-78	hypothetical protein PHYPA_020269
Pp3c21_18820	2.00	4.47e-18	hypothetical protein PHYPA_022665
Pp3c5_4560	2.00	4.61e-14	probable xyloglucan glycosyltransferase 5
Pp3c13_19370	2.00	8.37e-06	putative aconitate hydratase, cytoplasmic

Supporting Information Table S2: Complete list of downregulated genes in *Physcomitrium patens* during infection by *Colletotrichum gloeosporioides*.

Sequence ID	logFC	FDR	Description
Pp3c15_14300	-6.04	1.21e-134	scarecrow-like protein 28
Pp3c11_6580	-4.58	9.26e-139	hypothetical protein PHYPA_009755
Pp3c8_21760	-4.54	5.89e-39	6-phosphogluconate dehydrogenase, decarboxylating 3
Pp3c10_11330	-4.14	9.52e-76	phenylalanine ammonia-lyase-like
Pp3c5_9700	-4.02	9.11e-29	probable pectate lyase 5
Pp3c18_14350	-3.91	1.52e-63	hypothetical protein PHYPA_005345
Pp3c16_19790	-3.81	5.63e-18	metal tolerance protein C2
Pp3c17_5760	-3.68	5.79e-47	glycerophosphodiester phosphodiesterase GDPD1, chloroplastic
Pp3s350_10	-3.52	9.98e-12	high chlorophyll fluorescence phenotype 173
Pp3c13_15640	-3.41	8.28e-21	endoplasmic reticulum oxidoreductin-1
Pp3c6_10090	-3.40	1.25e-83	probable glycosyltransferase At5g03795
Pp3c15_22920	-3.39	6.59e-60	DNA polymerase delta catalytic subunit-like isoform X5
Pp3c13_13170	-3.35	5.72e-152	hypothetical protein PHYPA_019951
Pp3c13_16130	-3.32	7.40e-89	Mitochondrial import inner membrane translocase subunit tim-10 isoform 1
Pp3c13_15980	-3.32	3.57e-91	hypothetical protein PHYPA_019196
trnG	-3.28	3.32e-17	PSII L-protein
Pp3c24_13310	-3.24	6.58e-15	hypothetical protein PHYPA_018322
Pp3c22_16100	-3.21	1.29e-38	nuclear speckle RNA-binding protein B-like
Pp3c19_3900	-3.21	4.17e-45	protein NLP1
Pp3c4_8580	-3.21	6.56e-20	Sorghum bicolor protein targeted either to mitochondria or chloroplast proteins T50848
Pp3c26_5590	-3.20	2.82e-75	LEC14B protein
Pp3c20_17620	-3.19	2.69e-34	hypothetical protein PHYPA_027962
Pp3c13_15800	-3.18	4.44e-89	Protein terminal ear1-like
Pp3c10_25150	-3.18	1.12e-12	Retrovirus-related Pol polyprotein from transposon TNT 1-94
Pp3c1_17080	-3.18	4.91e-55	hypothetical protein PHYPA_018188
trnL	-3.16	1.08e-04	.
Pp3c13_16000	-3.13	2.12e-84	uncharacterized protein LOC112292868
Pp3c10_6880	-3.11	2.58e-52	protein odr-4 homolog
Pp3c13_15786	-3.11	4.92e-91	hypothetical protein PHYPA_020096
Pp3c18_18100	-3.10	1.97e-19	E3 ubiquitin-protein ligase PRT6-like isoform X2
Pp3c8_8000	-3.06	5.41e-28	uncharacterized protein LOC112286360
Pp3c24_8390	-3.05	2.56e-33	reverse transcriptase
Pp3c16_21250	-3.03	1.38e-51	probable thimet oligopeptidase isoform X1
Pp3c2_19460	-3.01	2.72e-16	NDR1/HIN1-like protein 6 isoform X1
trnE	-3.00	2.85e-03	PSII protein
Pp3c23_6560	-2.99	4.77e-11	proteasome subunit beta type-5

Pp3c16_19230	-2.99	6.68e-40	xyloglucan galactosyltransferase XLT2-like
Pp3c12_14210	-2.99	1.68e-07	uncharacterized protein LOC112285213
Pp3c13_16030	-2.96	3.70e-14	ribulose bisphosphate carboxylase small chain clone 512-like
Pp3c21_18980	-2.95	6.68e-72	hypothetical protein PHYPA_031026
Pp3c17_21350	-2.93	2.27e-18	nucleolar and coiled-body phosphoprotein 1-like
Pp3c3_10731	-2.93	1.34e-12	thioredoxin peroxidase 1
Pp3c10_3130	-2.90	1.60e-32	UPF0187 protein At3g61320, chloroplastic-like
Pp3c1_35321	-2.89	1.08e-11	CLP protease regulatory subunit CLPX1, mitochondrial isoform X2
Pp3c22_7170	-2.88	2.22e-20	hypothetical protein PHYPA_007440
Pp3c13_10520	-2.87	2.63e-17	transcription factor EGL1-like
Pp3c14_9180	-2.86	1.42e-68	YDG domain-containing protein At5g47150-like
Pp3c13_15790	-2.85	3.51e-32	phosphatidylinositol glycan anchor biosynthesis class U protein-like
Pp3c1_13170	-2.85	9.16e-73	hypothetical protein PHYPA_018919
Pp3c20_2280	-2.83	1.90e-32	filament-like plant protein 4 isoform X1
Pp3c1_2420	-2.83	3.13e-55	probable pectate lyase 5
Pp3c3_10730	-2.82	7.46e-12	4-hydroxy-tetrahydrodipicolinate synthase, chloroplastic
Pp3c6_23040	-2.82	4.29e-53	BRCT domain-containing protein
Pp3c9_5280	-2.82	4.54e-112	probable apyrase 7
Pp3c19_20760	-2.82	7.45e-18	probable tyrosine-protein phosphatase DG1060
trnV	-2.81	4.11e-09	PSI I-protein
Pp3c4_16840	-2.80	9.96e-41	putative 4-hydroxy-4-methyl-2-oxoglutarate aldolase 3
Pp3c13_21590	-2.80	8.95e-53	hypothetical protein PHYPA_019302
Pp3c4_17610	-2.79	5.29e-34	glutamate synthase 1 [NADH], chloroplastic isoform X1
Pp3c1_42230	-2.79	5.62e-15	pyrophosphate--fructose 6-phosphate 1-phosphotransferase subunit beta
Pp3c16_22640	-2.77	2.44e-14	probable membrane metalloprotease ARASP2, chloroplastic
Pp3c1_42420	-2.77	1.73e-14	hypothetical protein PHYPA_001911, partial
Pp3c12_24970	-2.76	5.42e-87	nuclear transcription factor Y subunit A-7 isoform X1
Pp3c4_1550	-2.75	2.86e-29	zinc finger CCCH domain-containing protein 64
Pp3c11_4850	-2.74	1.32e-91	GDP-fucose protein O-fucosyltransferase
Pp3c26_9830	-2.73	1.59e-07	bifunctional riboflavin kinase/FMN phosphatase
Pp3c26_15000	-2.73	4.40e-23	E3 ubiquitin-protein ligase HERC2-like isoform X1

Pp3c3_11210	-2.73	1.02e-29	probable xyloglucan endotransglucosylase/hydrolase protein 5
Pp3c13_23730	-2.72	9.69e-21	chromodomain-helicase-DNA-binding protein 9-like isoform X1
Pp3c4_1450	-2.71	1.98e-20	ethylene-responsive transcription factor ERF043-like
Pp3c15_2630	-2.71	1.12e-14	maturase-related protein (mitochondrion)
Pp3c20_21990	-2.71	2.06e-09	hypothetical protein PHYPA_022593
Pp3c4_510	-2.70	6.73e-27	NAD(P)H-quinone oxidoreductase subunit N, chloroplastic
Pp3c1_33180	-2.70	8.10e-12	protein JINGUBANG-like
Pp3c7_14650	-2.70	2.49e-14	hypothetical protein PHYPA_021858
Pp3c21_9970	-2.69	4.92e-93	long chain base biosynthesis protein 2a
Pp3c6_21520	-2.69	9.50e-45	hypothetical protein PHYPA_021944
Pp3c8_19540	-2.68	3.22e-65	B3 domain-containing protein Os11g0197600-like
Pp3c3_32420	-2.67	9.05e-37	hypothetical protein PHYPA_023159
Pp3c3_15560	-2.67	1.76e-24	uncharacterized protein LOC112284081
Pp3c17_1110	-2.66	1.38e-69	BUD13 homolog
Pp3c1_30790	-2.66	2.53e-29	hypothetical protein PHYPA_000579
Pp3c7_18910	-2.65	8.53e-06	abnormal spindle-like microcephaly-associated protein homolog isoform X1
Pp3c7_14450	-2.65	3.37e-66	retrotransposon protein, putative, Ty1-copia subclass
trnY	-2.65	3.75e-10	ribulose-1,5-bisphosphate carboxylase/oxygenase large subunit
Pp3c20_23430	-2.64	2.79e-63	triacylglycerol lipase 2-like
Pp3c19_5170	-2.63	1.87e-31	uncharacterized protein LOC112273267 isoform X1
Pp3c1_3940	-2.62	1.69e-16	predicted protein
Pp3c16_2280	-2.62	2.50e-14	hypothetical protein PHYPA_024324
Pp3c19_10230	-2.62	3.16e-45	uncharacterized protein LOC112273105
Pp3c11_9120	-2.61	5.03e-58	copalyl diphosphate synthase 1-like isoform X1
Pp3c23_1130	-2.61	9.64e-42	Retrovirus-related Pol polyprotein from transposon TNT 1-94
Pp3c10_16560	-2.61	6.18e-75	hypothetical protein PHYPA_030281
matK	-2.60	1.12e-30	ribosomal protein L22
Pp3c2_13970	-2.58	1.37e-30	U11/U12 small nuclear ribonucleoprotein 35 kDa protein
Pp3c11_4920	-2.57	3.58e-24	probable methyltransferase PMT21
Pp3c25_9350	-2.54	5.15e-53	hypothetical protein PHYPA_011202
Pp3c19_10340	-2.54	1.02e-33	hypothetical protein PHYPA_024550
Pp3c20_2620	-2.54	8.90e-42	uncharacterized protein LOC112275494
Pp3c19_14070	-2.53	2.14e-68	RING/FYVE/PHD zinc finger superfamily protein
Pp3c16_20090	-2.53	1.75e-20	hypothetical protein PHYPA_024218
Pp3c4_14700	-2.52	5.86e-45	protein TIFY 6a-like

Pp3c9_5310	-2.52	8.46e-101	hypothetical protein PHYPA_025947, partial
Pp3c1_29500	-2.51	1.77e-28	LRR receptor-like serine/threonine-protein kinase FLS2
Pp3c3_2620	-2.51	1.95e-67	ubiquitin-like-specific protease 1D
trnK	-2.50	4.85e-32	ribosomal protein L21
Pp3c20_1150	-2.49	1.65e-15	predicted protein
Pp3c19_20080	-2.49	2.31e-128	uncharacterized protein LOC112273157
Pp3c5_28500	-2.48	1.12e-62	catalase isozyme 2-like
Pp3c15_19080	-2.48	6.27e-29	cationic amino acid transporter 2, vacuolar-like
Pp3c16_19430	-2.48	5.97e-20	hypothetical protein PHYPA_024188
Pp3c9_4760	-2.48	5.34e-40	hypothetical protein PHYPA_025921
Pp3c5_640	-2.48	6.54e-35	hypothetical protein PHYPA_027224
Pp3c11_6250	-2.47	9.78e-33	glycosyltransferase-like protein
Pp3c20_20120	-2.47	5.58e-17	myosin-2 heavy chain-like
Pp3c7_21370	-2.47	6.47e-12	paired amphipathic helix protein Sin3-like 4 isoform X1
atpB	-2.47	1.09e-55	PSI P700 apoprotein A1
Pp3c2_19150	-2.47	6.73e-14	hypothetical protein PHYPA_016583
Pp3c15_22730	-2.46	1.72e-61	GDSL esterase/lipase At4g16230-like
Pp3c4_11854	-2.46	2.70e-56	HMG1/2-like protein
Pp3c5_3210	-2.46	2.33e-66	probable non-specific lipid-transfer protein AKCS9
Pp3c2_4100	-2.46	3.93e-13	putative receptor protein kinase CRINKLY4
Pp3c25_1840	-2.46	3.74e-52	hypothetical protein PHYPA_011444
Pp3c4_23200	-2.45	3.96e-08	predicted protein
Pp3c9_5060	-2.45	1.22e-77	probable xyloglucan endotransglucosylase/hydrolase protein 27
Pp3c15_22720	-2.45	3.95e-64	hypothetical protein PHYPA_003359
Pp3c8_14190	-2.44	5.77e-44	diphthine methyltransferase homolog
Pp3c12_22390	-2.44	1.82e-45	probable protein phosphatase 2C 51 isoform X1
Pp3c27_5030	-2.44	3.07e-95	protein fluG-like
Pp3c19_12910	-2.44	1.60e-53	transmembrane protein 53
Pp3c3_16280	-2.44	1.96e-28	V-type proton ATPase subunit D-like
Pp3c3_36450	-2.43	1.66e-20	hypothetical protein PHYPA_023346
Pp3c3_25110	-2.43	3.34e-63	hypothetical protein PHYPA_029660
Pp3c1_42250	-2.42	4.33e-10	60S ribosomal protein L7-2
Pp3c16_5630	-2.42	4.61e-12	AC091247_1putative polyprotein, 5'-
Pp3c4_13150	-2.41	3.30e-22	cytochrome c oxidase assembly protein COX11, mitochondrial
Pp3s775_10	-2.41	3.55e-04	hypothetical protein PHYPA_021160
Pp3c19_14710	-2.41	6.52e-38	hypothetical protein PHYPA_025308
Pp3c4_30220	-2.40	3.46e-30	bifunctional aspartokinase/homoserine dehydrogenase 1, chloroplastic-like
Pp3c2_17510	-2.40	1.05e-23	Proline-rich protein 1
Pp3c10_17170	-2.40	4.49e-11	WEB family protein At1g12150-like
Pp3c4_13140	-2.40	3.60e-22	hypothetical protein PHYPA_020859

Pp3c19_11600	-2.40	1.87e-71	hypothetical protein PHYPA_025188
Pp3c2_9980	-2.39	5.43e-29	glycerol-3-phosphate 2-O-acyltransferase 6-like
Pp3c13_4170	-2.39	1.87e-15	Retrovirus-related Pol polyprotein from transposon TNT 1-94
Pp3c12_4580	-2.39	3.26e-25	hypothetical protein PHYPA_019588
Pp3c12_7010	-2.39	3.61e-21	hypothetical protein PHYPA_019679, partial
Pp3c1_22450	-2.39	2.01e-81	---NA---
atpE	-2.38	3.71e-59	PSI P700 apoprotein A2
Pp3c19_3732	-2.38	3.56e-07	serine/threonine-protein phosphatase 4 regulatory subunit 2 isoform X2
Pp3c20_18320	-2.38	7.05e-28	hypothetical protein PHYPA_028003, partial
Pp3c3_24631	-2.37	4.78e-08	F-box protein At2g26850-like
Pp3c25_6730	-2.37	1.02e-69	pumilio homolog 1-like
Pp3c7_27000	-2.37	2.28e-61	Putative non-structural 4
Pp3c13_23660	-2.37	1.58e-16	hypothetical protein PHYPA_019393
Pp3c6_16970	-2.36	3.44e-29	ras-related protein RABH1e
Pp3c23_1972	-2.36	2.57e-13	SUN domain-containing protein 1
Pp3c4_8470	-2.36	7.29e-77	transcription factor bHLH49-like
Pp3c3_24618	-2.35	3.19e-12	golgin subfamily A member 4-like isoform X1
Pp3c15_21090	-2.35	2.20e-06	probable 1-deoxy-D-xylulose-5-phosphate synthase, chloroplastic
Pp3c3_25080	-2.35	4.13e-38	sialidase-like isoform X2
Pp3c16_11740	-2.35	5.83e-119	Transmembrane protein
Pp3c22_6380	-2.34	6.93e-83	ethylene-responsive transcription factor
Pp3c22_17930	-2.34	2.91e-36	Retrovirus-related Pol polyprotein from transposon TNT 1-94
Pp3c1_31440	-2.34	1.82e-65	Ribosomal protein L23/L15e family protein, putative
Pp3c24_20800	-2.34	1.89e-37	subtilisin-like protease SBT2.5
Pp3c21_4230	-2.33	1.68e-52	kinesin-like protein KIN-12E
Pp3c20_13600	-2.33	3.48e-18	putative lipoate-protein ligase A
Pp3c12_9500	-2.32	2.64e-67	ubiquitin carboxyl-terminal hydrolase 12
Pp3c4_3720	-2.31	1.38e-64	probable amino-acid acetyltransferase NAGS1, chloroplastic
Pp3c13_6570	-2.31	2.65e-59	protease Do-like 9
Pp3c4_22170	-2.31	1.39e-12	reverse transcriptase
Pp3c22_14530	-2.31	1.48e-80	TMV resistance protein N-like
Pp3c16_4550	-2.31	7.45e-63	hypothetical protein PHYPA_023635
Pp3c15_14090	-2.30	3.26e-10	importin subunit alpha-1-like isoform X1
Pp3c3_38220	-2.30	3.90e-12	protein SUPPRESSOR OF npr1-1, CONSTITUTIVE 1-like
Pp3c9_19520	-2.30	1.78e-57	hypothetical protein PHYPA_026402
Pp3c4_20230	-2.29	3.17e-67	disease resistance protein TAO1-like
Pp3c16_21270	-2.29	1.53e-14	fasciclin-like protein
Pp3c18_14800	-2.29	1.30e-27	peroxidase A2-like
Pp3c3_37470	-2.29	2.31e-41	protein PHR1-LIKE 2-like isoform X1

Pp3c19_20070	-2.29	8.81e-78	sulfhydryl oxidase 2-like isoform X1
Pp3c6_3820	-2.29	6.75e-07	transcription factor MYB3R-5-like isoform X3
Pp3c14_20830	-2.28	8.11e-73	mediator of RNA polymerase II transcription subunit 31-like
Pp3c23_6900	-2.28	2.12e-13	protein DOG1-like 3
Pp3c23_2010	-2.28	3.63e-24	UPF0643 protein PB2B2.08-like
Pp3c12_11700	-2.28	7.54e-58	hypothetical protein PHYPA_022665
Pp3c23_14000	-2.27	9.13e-08	biotin synthase
Pp3c3_31900	-2.27	2.69e-21	histone H3.3 isoform X1
Pp3c5_12950	-2.27	2.27e-13	probable pectinesterase 53
Pp3c25_400	-2.27	2.98e-40	protein TSS
Pp3c1_8210	-2.27	3.49e-23	UPF0051 protein in atpA 3'region-like
Pp3c19_17080	-2.26	8.24e-28	Alpha-D-phosphohexomutase superfamily
Pp3c3_29550	-2.26	6.85e-31	DNA repair endonuclease UVH1 isoform X1
Pp3c11_10950	-2.26	2.29e-45	myb family transcription factor PHL7-like
Pp3c15_12410	-2.26	1.24e-73	ras-related protein RABC1-like
Pp3c3_24600	-2.26	4.31e-13	transmembrane emp24 domain-containing protein p24beta2-like
Pp3c5_2110	-2.26	1.50e-16	hypothetical protein PHYPA_013425
Pp3c16_4520	-2.26	4.74e-57	hypothetical protein PHYPA_023633
Pp3c16_8630	-2.26	9.32e-24	hypothetical protein PHYPA_023856
Pp3c13_18780	-2.25	2.31e-24	AT-rich interactive domain-containing protein 3-like isoform X1
Pp3c25_1450	-2.25	9.26e-48	conserved oligomeric Golgi complex subunit 3
Pp3c16_3320	-2.25	1.18e-11	cytochrome c
Pp3c6_6460	-2.25	1.03e-32	iron-sulfur cluster assembly protein 1-like
Pp3c16_3930	-2.25	4.91e-48	serine/threonine-protein kinase BSK1-like isoform X1
Pp3c24_12360	-2.25	3.71e-62	hypothetical protein PHYPA_014819, partial
Pp3c3_35020	-2.24	5.79e-28	C2 domain-containing protein At1g53590-like
Pp3c5_14300	-2.24	1.36e-47	HECT-domain-containing protein
Pp3c16_10550	-2.24	2.37e-56	plastid division protein PDV2-like
Pp3c19_4310	-2.24	2.93e-25	predicted protein
Pp3c5_8580	-2.24	3.44e-16	probable sucrose-phosphatase 2 isoform X1
Pp3c4_3730	-2.24	1.71e-30	hypothetical protein PHYPA_013738
Pp3c1_18860	-2.24	7.14e-46	hypothetical protein PHYPA_031299
Pp3c11_10480	-2.24	2.27e-61	uncharacterized protein LOC112280916
Pp3c19_3840	-2.23	1.33e-100	AUGMIN subunit3
Pp3c23_2890	-2.23	2.44e-12	lysophospholipid acyltransferase LPEAT1 isoform X1
Pp3c2_24270	-2.23	2.17e-57	plant intracellular Ras-group-related LRR protein 1-like
Pp3c12_5820	-2.23	9.37e-10	hypothetical protein PHYPA_019644
Pp3c20_22420	-2.23	5.20e-69	uncharacterized protein LOC112275598

Pp3c11_15160	-2.22	3.19e-24	26S proteasome non-ATPase regulatory subunit 11 homolog
Pp3c2_28940	-2.22	2.04e-19	60S ribosomal protein L21-1
Pp3c16_8870	-2.22	7.86e-08	probable 26S proteasome subunit YTA6 isoform X1
Pp3c3_6760	-2.22	1.48e-12	RHOMBOID-like protein 13
Pp3c20_13120	-2.22	1.36e-53	hypothetical protein AXX17_ATUG04770
Pp3s116_10	-2.22	6.07e-68	hypothetical protein PHYPA_000255
Pp3c25_1440	-2.22	5.90e-63	hypothetical protein PHYPA_018282
Pp3c20_4990	-2.22	5.06e-59	hypothetical protein PHYPA_028382, partial
Pp3c6_11210	-2.22	1.27e-40	uncharacterized protein LOC112275223
Pp3c9_19800	-2.21	3.62e-38	dnaJ protein homolog
Pp3c9_20000	-2.21	4.60e-21	microtubule-associated protein 70-1-like
Pp3c11_10680	-2.21	6.00e-69	probable 3-hydroxyisobutyrate dehydrogenase-like 1, mitochondrial
Pp3c3_3230	-2.21	3.34e-07	ribosome biogenesis protein BOP1 homolog
Pp3c20_4670	-2.21	3.99e-34	Serine/threonine protein phosphatase 2A 55 kDa regulatory subunit B beta isoform
Pp3c18_9930	-2.20	7.63e-12	12-oxophytodienoate reductase 3
Pp3c19_5900	-2.20	4.31e-10	beta-amylase 2, chloroplastic-like
Pp3c24_12430	-2.20	7.53e-21	biotin carboxyl carrier protein of acetyl-CoA carboxylase
Pp3s242_10	-2.20	5.18e-56	mitochondrial dicarboxylate/tricarboxylate transporter DTC
Pp3c3_1650	-2.20	1.17e-08	TMV resistance protein N-like
Pp3c21_9980	-2.20	6.01e-98	hypothetical protein PHYPA_007864
Pp3c2_24160	-2.20	7.76e-60	uncharacterized protein LOC112289304
Pp3c14_3220	-2.19	2.81e-04	elongation factor 1-alpha-like
Pp3c5_10730	-2.19	7.90e-53	GDSL esterase/lipase At5g55050-like
Pp3c24_8780	-2.19	3.51e-35	protein translocase subunit SECA2, chloroplastic isoform X2
Pp3c11_21460	-2.19	2.26e-14	UPF0613 protein PB24D3.06c
Pp3c6_20300	-2.19	4.00e-47	WD40 repeat-like protein
Pp3c11_23670	-2.18	3.74e-52	chloroplast envelope membrane protein-like
Pp3c19_4313	-2.18	2.92e-23	dynein heavy chain 7, axonemal-like
Pp3s116_40	-2.18	7.11e-55	RGG repeats nuclear RNA binding protein A-like
Pp3c11_4830	-2.18	1.20e-14	vegetative cell wall protein gp1-like
Pp3c10_3020	-2.18	5.87e-70	hypothetical protein PHYPA_005627
Pp3c20_8260	-2.18	1.91e-48	hypothetical protein PHYPA_027651
Pp3c22_11130	-2.17	3.07e-12	Drug/metabolite transporter
Pp3c16_12600	-2.17	1.74e-44	galactan beta-1,4-galactosyltransferase GALS1-like
Pp3c13_5930	-2.17	2.39e-56	heat shock protein 83
Pp3c5_7150	-2.17	5.36e-47	KAT8 regulatory NSL complex subunit 3
Pp3c21_12100	-2.17	1.47e-92	LRR receptor-like serine/threonine-protein kinase GSO1 isoform X1
Pp3c20_1900	-2.17	5.53e-18	peroxisomal 2,4-dienoyl-CoA reductase-like

Pp3c3_29530	-2.17	2.78e-28	probable splicing factor 3A subunit 1
Pp3c7_120	-2.17	7.82e-32	protein ENHANCED DISEASE RESISTANCE 2-like
trnF	-2.17	1.56e-04	PSII 47kDa protein
Pp3c19_1490	-2.17	1.28e-30	universal stress protein PHOS34-like
Pp3c14_12070	-2.17	9.16e-60	hypothetical protein PHYPA_017420
Pp3c17_20310	-2.16	7.77e-42	ethylene-responsive transcription factor ERF084-like
Pp3c22_2660	-2.16	4.68e-27	GA20 oxidase-like protein
Pp3c26_1140	-2.16	3.56e-20	nuclear-pore anchor
Pp3c5_7180	-2.16	1.16e-46	phenylalanine ammonia-lyase-like
Pp3c22_7690	-2.16	6.71e-88	predicted protein
Pp3c3_34790	-2.16	4.39e-51	hypothetical protein PHYPA_023263
Pp3c5_7110	-2.16	4.27e-47	uncharacterized protein LOC112274862 isoform X2
Pp3c12_20650	-2.15	2.35e-62	condensin complex subunit 2-like
Pp3c22_10850	-2.15	1.36e-51	disease resistance-like protein CSA1
Pp3c6_26200	-2.15	4.46e-35	histone H2B.8-like
Pp3c17_14680	-2.15	7.98e-50	inactive protein kinase SELMODRAFT_444075-like
Pp3c26_630	-2.15	2.18e-43	predicted protein
Pp3c1_7380	-2.15	8.56e-88	probable protein phosphatase 2C 15 isoform X1
Pp3c6_200	-2.15	2.02e-12	Retrovirus-related Pol polyprotein from transposon TNT 1-94
Pp3c17_14681	-2.15	6.89e-50	hypothetical protein PHYPA_004393
Pp3c11_2650	-2.15	1.38e-33	hypothetical protein PHYPA_009679
Pp3c24_13190	-2.14	4.89e-15	60S ribosomal protein L28-1-like
Pp3c11_12080	-2.14	9.75e-70	eukaryotic translation initiation factor 3 subunit C-like
Pp3c6_6830	-2.14	1.15e-57	F-box protein At1g47056-like
Pp3c20_15220	-2.14	1.27e-13	---NA---
Pp3c18_13090	-2.14	1.20e-46	uncharacterized protein LOC112279900
Pp3s1006_30	-2.14	2.30e-04	uncharacterized protein LOC112290578 isoform X3
Pp3c12_15820	-2.13	3.63e-30	FT-interacting protein 1-like
Pp3c1_32200	-2.13	2.40e-50	germin-like protein 1-3
Pp3c10_15730	-2.13	1.50e-86	polyadenylate-binding protein-interacting protein 7-like
Pp3c5_19300	-2.13	2.96e-25	hypothetical protein PHYPA_013332
Pp3c1_27990	-2.13	1.45e-73	uncharacterized protein LOC112285443 isoform X1
Pp3c25_15260	-2.12	1.67e-50	CAX-interacting protein 4-like
Pp3c16_15270	-2.12	1.57e-79	DEAD-box ATP-dependent RNA helicase 42
Pp3c7_23980	-2.12	2.66e-84	DNA-(apurinic or apyrimidinic site) lyase
Pp3c21_6200	-2.12	4.59e-19	Glycosyl hydrolase, five-bladed beta-propellor domain-containing protein

Pp3c13_20640	-2.12	1.79e-30	Prestalk-specific protein tagB
Pp3c10_4440	-2.12	2.56e-27	probable protein phosphatase 2C 60 isoform X1
Pp3c18_8820	-2.12	4.66e-61	Retrovirus-related Pol polyprotein from transposon TNT 1-94
Pp3c15_16700	-2.12	9.37e-46	ribosomal protein L16 (mitochondrion)
Pp3c23_1980	-2.12	5.75e-15	sodium-dependent phosphate transport protein 1, chloroplastic
Pp3c2_4940	-2.12	1.08e-18	uncharacterized endoplasmic reticulum membrane protein YGL010W-like
Pp3c4_28370	-2.12	1.79e-69	vasohibin-2 isoform X3
Pp3c13_15240	-2.12	4.59e-13	hypothetical protein PHYPA_020063
Pp3c3_35180	-2.11	1.21e-70	lanC-like protein GCL1
Pp3c9_19950	-2.11	1.01e-31	putative clathrin assembly protein At5g35200
Pp3c4_31680	-2.11	1.02e-06	RNA helicase nonsense mRNA reducing factor
Pp3c13_2310	-2.11	7.83e-43	SAL1 phosphatase-like
Pp3c2_22630	-2.11	1.37e-33	hypothetical protein PHYPA_015741
Pp3c1_1330	-2.11	4.93e-64	hypothetical protein PHYPA_018926
Pp3c7_24650	-2.11	9.44e-38	hypothetical protein PHYPA_029322, partial
Pp3c20_5760	-2.11	2.29e-51	---NA---
Pp3c7_11090	-2.10	5.50e-57	aspartyl protease family protein 2-like
Pp3c24_18690	-2.10	4.38e-40	autophagy-related protein 8C
Pp3c14_11030	-2.10	6.64e-70	calnexin homolog
Pp3c8_5370	-2.10	9.00e-33	F-box only protein 6-like
Pp3c4_8171	-2.10	6.22e-64	hydroxyproline O-arabinosyltransferase 3-like
Pp3c6_18710	-2.10	1.81e-39	proteasome subunit alpha type-7
Pp3c12_22330	-2.10	8.35e-06	protein ECERIFERUM 3
Pp3c26_6740	-2.10	3.75e-36	serine carboxypeptidase-like
Pp3c12_9890	-2.10	3.14e-49	serine/threonine-protein kinase HT1-like
Pp3c7_11100	-2.10	5.63e-56	trihelix transcription factor ASR3-like
Pp3c18_13350	-2.10	2.53e-23	---NA---
Pp3c10_9740	-2.09	6.07e-68	casein kinase 1-like protein 1
Pp3c7_22220	-2.09	1.12e-07	pol-like protein
Pp3c1_4010	-2.09	8.23e-53	U-box domain-containing protein 43-like
Pp3c27_6070	-2.09	1.27e-17	hypothetical protein PHYPA_001573, partial
Pp3c7_12850	-2.09	1.50e-42	hypothetical protein PHYPA_021789
Pp3c19_12750	-2.09	4.88e-39	hypothetical protein PHYPA_025225
Pp3c14_7930	-2.08	7.74e-25	aldehyde oxidase GLOX-like
Pp3c17_4440	-2.08	1.21e-10	arginyl-tRNA--protein transferase 2-like
Pp3c12_5220	-2.08	7.77e-15	enoyl-[acyl-carrier-protein] reductase [NADH] 1, chloroplastic-like
Pp3c24_20960	-2.08	1.44e-41	protein DETOXIFICATION 16-like
Pp3c8_1940	-2.08	4.02e-55	protein GLE1
Pp3c20_2980	-2.08	9.54e-47	protein NLP1
Pp3c4_8330	-2.08	5.06e-57	receptor kinase-like protein

Pp3c10_13720	-2.08	9.67e-04	ureide permease 1-like
Pp3c26_12570	-2.08	4.42e-68	hypothetical protein PHYPA_011344
Pp3c18_8240	-2.08	1.23e-57	hypothetical protein PHYPA_025065
Pp3c13_14980	-2.08	3.95e-44	---NA---
Pp3c1_30428	-2.07	3.66e-16	ATP-dependent RNA helicase DBP2-like
Pp3c17_3830	-2.07	2.29e-49	transcription factor TCP15-like
Pp3c12_8680	-2.07	2.94e-13	transmembrane protein 120 homolog
Pp3c16_3080	-2.07	5.24e-10	zinc finger CCCH domain-containing protein 35-like
Pp3c2_17110	-2.07	1.09e-51	hypothetical protein PHYPA_016472
Pp3c1_14760	-2.07	4.18e-23	hypothetical protein PHYPA_017974
Pp3c27_440	-2.06	8.19e-13	deoxyhypusine synthase isoform X1
Pp3c15_10950	-2.06	3.96e-21	mitogen-activated protein kinase kinase kinase YODA-like
Pp3c13_1510	-2.06	4.84e-06	Retrovirus-related Pol polyprotein from transposon TNT 1-94
Pp3c16_2080	-2.06	9.73e-55	Retrovirus-related Pol polyprotein from transposon TNT 1-94
Pp3c7_6700	-2.06	4.42e-05	transcription factor GAMYB-like
Pp3c15_12810	-2.06	2.71e-47	tubulin alpha-3 chain-like
Pp3c2_29140	-2.06	3.53e-23	ubiquitin-conjugating enzyme E2 2
Pp3c11_15790	-2.06	9.85e-49	V-type proton ATPase subunit a3-like isoform X1
Pp3c12_4540	-2.06	2.47e-58	hypothetical protein PHYPA_019585
Pp3c9_5650	-2.05	1.08e-06	60S ribosomal protein L37-3
Pp3c1_19190	-2.05	7.97e-64	ammonium transporter 1 member 3-like
Pp3c24_10800	-2.05	1.46e-28	Embryogenesis-associated protein EMB8
Pp3c16_23250	-2.05	1.27e-28	GATA zinc finger protein
Pp3c7_14840	-2.05	5.01e-21	putative dihydroflavonol-4-reductase
Pp3c5_2700	-2.05	4.62e-37	putative nuclease HARBI1
Pp3c7_20320	-2.05	5.89e-43	transcription elongation factor B polypeptide 3
Pp3c23_22230	-2.05	2.48e-39	hypothetical protein PHYPA_014494
Pp3c23_6070	-2.05	1.52e-57	hypothetical protein PHYPA_015644
Pp3c24_5330	-2.05	4.81e-07	hypothetical protein PHYPA_018827
Pp3c1_18350	-2.05	1.10e-17	ATP-dependent DNA helicase SRS2-like protein At4g25120
Pp3c24_19080	-2.04	7.67e-29	calcium sensing receptor, chloroplastic-like
Pp3c7_23810	-2.04	1.18e-61	calmodulin-binding ion transporter-like protein
Pp3c1_13800	-2.04	2.10e-07	G-type lectin S-receptor-like serine/threonine-protein kinase SD2-5
Pp3c24_17170	-2.04	1.36e-34	nuclear pore complex protein NUP54
Pp3c3_34180	-2.04	9.15e-52	protein CONSERVED IN THE GREEN LINEAGE AND DIATOMS 27, chloroplastic-like
Pp3c24_12180	-2.04	1.34e-50	hypothetical protein PHYPA_014424
Pp3c2_12860	-2.04	4.31e-08	hypothetical protein PHYPA_016259

Pp3c6_28830	-2.03	1.53e-75	dihydroceramide fatty acyl 2-hydroxylase FAH1-like
Pp3c9_9200	-2.03	1.75e-40	electron transfer flavoprotein-ubiquinone oxidoreductase, mitochondrial-like isoform X1
Pp3c9_20020	-2.03	1.62e-25	microtubule-associated protein 70-1-like
Pp3c21_11130	-2.03	2.79e-42	phospholipase D alpha 1
Pp3c9_15440	-2.03	7.52e-47	polyphenol oxidase, chloroplastic-like
Pp3c1_4350	-2.03	8.01e-35	hypothetical protein PHYPA_001949, partial
Pp3c4_20310	-2.03	1.57e-09	hypothetical protein PHYPA_025225
Pp3c10_22780	-2.03	3.72e-35	---NA---
Pp3c1_3040	-2.02	3.08e-17	ACT domain-containing protein ACR3-like isoform X4
Pp3c6_10350	-2.02	3.53e-12	chlorophyllide a oxygenase, chloroplastic
Pp3c26_11090	-2.02	3.32e-40	cytosolic purine 5'-nucleotidase-like isoform X1
Pp3c11_4100	-2.02	2.47e-29	ENHANCER OF AG-4 protein 2
Pp3c24_19680	-2.02	5.21e-13	J domain-containing protein spf31
Pp3c18_7850	-2.02	2.26e-42	myosin heavy chain kinase A-like isoform X3
Pp3c25_4520	-2.02	9.12e-21	protein MANNAN SYNTHESIS-RELATED 1-like isoform X2
Pp3c13_24600	-2.02	2.91e-31	stress-response A/B barrel domain-containing protein UP3-like
Pp3c12_25900	-2.02	6.36e-37	hypothetical protein PHYPA_010127
Pp3c13_16600	-2.02	9.42e-07	hypothetical protein PHYPA_020126
Pp3c12_21540	-2.02	1.22e-55	uncharacterized protein LOC112284578
Pp3c14_25660	-2.02	1.31e-13	uncharacterized protein LOC112290419
Pp3c4_22100	-2.01	1.06e-51	calcineurin B-like protein 3
Pp3c8_17920	-2.01	5.43e-28	DNA-directed RNA polymerase II subunit 4
Pp3c4_8159	-2.01	3.18e-53	leucine-rich repeat receptor-like serine/threonine/tyrosine-protein kinase SOBIR1
Pp3c15_16860	-2.01	8.11e-06	oxygen-evolving enhancer protein 2, chloroplastic-like
Pp3c25_7030	-2.01	1.56e-36	probable magnesium transporter NIPA8
Pp3c2_4740	-2.01	1.10e-56	protein S-acyltransferase 21
Pp3c5_3190	-2.01	9.03e-08	Thioredoxin superfamily protein isoform 1
Pp3c3_26700	-2.01	7.65e-14	vicilin-like seed storage protein At2g18540 isoform X2
Pp3c15_11070	-2.01	2.57e-45	hypothetical protein PHYPA_002883
Pp3c6_10610	-2.01	1.04e-31	hypothetical protein PHYPA_007844
Pp3c2_13420	-2.01	1.63e-24	hypothetical protein PHYPA_016289
Pp3c6_27020	-2.01	1.31e-04	hypothetical protein PHYPA_021549
Pp3c19_4880	-2.01	1.56e-27	hypothetical protein PHYPA_024468
Pp3c6_21060	-2.00	7.07e-48	diphthamide biosynthesis protein 3-like
Pp3c17_23380	-2.00	1.63e-37	mitochondrial inner membrane protease subunit 2-like isoform X1

Pp3c3_1020	-2.00	3.03e-30	putative DD1A protein
Pp3c12_6700	-2.00	3.54e-47	serine/threonine-protein kinase 16-like
Pp3c16_5790	-2.00	3.28e-39	squalene monooxygenase-like
Pp3c11_800	-2.00	2.17e-30	hypothetical protein PHYPA_010493
Pp3c16_16040	-2.00	2.59e-70	hypothetical protein PHYPA_024003
Pp3c15_14300	-6.04	1.21e-134	scarecrow-like protein 28
Pp3c11_6580	-4.58	9.26e-139	hypothetical protein PHYPA_009755
Pp3c8_21760	-4.54	5.89e-39	6-phosphogluconate dehydrogenase, decarboxylating 3
Pp3c10_11330	-4.14	9.52e-76	phenylalanine ammonia-lyase-like
Pp3c5_9700	-4.02	9.11e-29	probable pectate lyase 5
Pp3c18_14350	-3.91	1.52e-63	hypothetical protein PHYPA_005345
Pp3c16_19790	-3.81	5.63e-18	metal tolerance protein C2
Pp3c17_5760	-3.68	5.79e-47	glycerophosphodiester phosphodiesterase GDPD1, chloroplastic
Pp3s350_10	-3.52	9.98e-12	high chlorophyll fluorescence phenotype 173
Pp3c13_15640	-3.41	8.28e-21	endoplasmic reticulum oxidoreductin-1
Pp3c6_10090	-3.40	1.25e-83	probable glycosyltransferase At5g03795
Pp3c15_22920	-3.39	6.59e-60	DNA polymerase delta catalytic subunit-like isoform X5
Pp3c13_13170	-3.35	5.72e-152	hypothetical protein PHYPA_019951
Pp3c13_16130	-3.32	7.40e-89	Mitochondrial import inner membrane translocase subunit tim-10 isoform 1
Pp3c13_15980	-3.32	3.57e-91	hypothetical protein PHYPA_019196
trnG	-3.28	3.32e-17	PSII L-protein
Pp3c24_13310	-3.24	6.58e-15	hypothetical protein PHYPA_018322
Pp3c22_16100	-3.21	1.29e-38	nuclear speckle RNA-binding protein B-like
Pp3c19_3900	-3.21	4.17e-45	protein NLP1
Pp3c4_8580	-3.21	6.56e-20	Sorghum bicolor protein targeted either to mitochondria or chloroplast proteins T50848
Pp3c26_5590	-3.20	2.82e-75	LEC14B protein
Pp3c20_17620	-3.19	2.69e-34	hypothetical protein PHYPA_027962
Pp3c13_15800	-3.18	4.44e-89	Protein terminal ear1-like
Pp3c10_25150	-3.18	1.12e-12	Retrovirus-related Pol polyprotein from transposon TNT 1-94
Pp3c1_17080	-3.18	4.91e-55	hypothetical protein PHYPA_018188
trnL	-3.16	1.08e-04	.
Pp3c13_16000	-3.13	2.12e-84	uncharacterized protein LOC112292868
Pp3c10_6880	-3.11	2.58e-52	protein odr-4 homolog
Pp3c13_15786	-3.11	4.92e-91	hypothetical protein PHYPA_020096
Pp3c18_18100	-3.10	1.97e-19	E3 ubiquitin-protein ligase PRT6-like isoform X2
Pp3c8_8000	-3.06	5.41e-28	uncharacterized protein LOC112286360
Pp3c24_8390	-3.05	2.56e-33	reverse transcriptase
Pp3c16_21250	-3.03	1.38e-51	probable thimet oligopeptidase isoform X1
Pp3c2_19460	-3.01	2.72e-16	NDR1/HIN1-like protein 6 isoform X1
trnE	-3.00	2.85e-03	PSII protein

Pp3c23_6560	-2.99	4.77e-11	proteasome subunit beta type-5
Pp3c16_19230	-2.99	6.68e-40	xyloglucan galactosyltransferase XLT2-like
Pp3c12_14210	-2.99	1.68e-07	uncharacterized protein LOC112285213
Pp3c13_16030	-2.96	3.70e-14	ribulose bisphosphate carboxylase small chain clone 512-like
Pp3c21_18980	-2.95	6.68e-72	hypothetical protein PHYPA_031026
Pp3c17_21350	-2.93	2.27e-18	nucleolar and coiled-body phosphoprotein 1-like
Pp3c3_10731	-2.93	1.34e-12	thioredoxin peroxidase 1
Pp3c10_3130	-2.90	1.60e-32	UPF0187 protein At3g61320, chloroplastic-like
Pp3c1_35321	-2.89	1.08e-11	CLP protease regulatory subunit CLPX1, mitochondrial isoform X2
Pp3c22_7170	-2.88	2.22e-20	hypothetical protein PHYPA_007440
Pp3c13_10520	-2.87	2.63e-17	transcription factor EGL1-like
Pp3c14_9180	-2.86	1.42e-68	YDG domain-containing protein At5g47150-like
Pp3c13_15790	-2.85	3.51e-32	phosphatidylinositol glycan anchor biosynthesis class U protein-like
Pp3c1_13170	-2.85	9.16e-73	hypothetical protein PHYPA_018919
Pp3c20_2280	-2.83	1.90e-32	filament-like plant protein 4 isoform X1
Pp3c1_2420	-2.83	3.13e-55	probable pectate lyase 5
Pp3c3_10730	-2.82	7.46e-12	4-hydroxy-tetrahydrodipicolinate synthase, chloroplastic
Pp3c6_23040	-2.82	4.29e-53	BRCT domain-containing protein
Pp3c9_5280	-2.82	4.54e-112	probable apyrase 7
Pp3c19_20760	-2.82	7.45e-18	probable tyrosine-protein phosphatase DG1060
trnV	-2.81	4.11e-09	PSI I-protein
Pp3c4_16840	-2.80	9.96e-41	putative 4-hydroxy-4-methyl-2-oxoglutarate aldolase 3
Pp3c13_21590	-2.80	8.95e-53	hypothetical protein PHYPA_019302
Pp3c4_17610	-2.79	5.29e-34	glutamate synthase 1 [NADH], chloroplastic isoform X1
Pp3c1_42230	-2.79	5.62e-15	pyrophosphate--fructose 6-phosphate 1-phosphotransferase subunit beta
Pp3c16_22640	-2.77	2.44e-14	probable membrane metalloprotease ARASP2, chloroplastic
Pp3c1_42420	-2.77	1.73e-14	hypothetical protein PHYPA_001911, partial
Pp3c12_24970	-2.76	5.42e-87	nuclear transcription factor Y subunit A-7 isoform X1
Pp3c4_1550	-2.75	2.86e-29	zinc finger CCCH domain-containing protein 64
Pp3c11_4850	-2.74	1.32e-91	GDP-fucose protein O-fucosyltransferase
Pp3c26_9830	-2.73	1.59e-07	bifunctional riboflavin kinase/FMN phosphatase
Pp3c26_15000	-2.73	4.40e-23	E3 ubiquitin-protein ligase HERC2-like isoform X1

Pp3c3_11210	-2.73	1.02e-29	probable xyloglucan endotransglucosylase/hydrolase protein 5
Pp3c13_23730	-2.72	9.69e-21	chromodomain-helicase-DNA-binding protein 9-like isoform X1
Pp3c4_1450	-2.71	1.98e-20	ethylene-responsive transcription factor ERF043-like
Pp3c15_2630	-2.71	1.12e-14	maturase-related protein (mitochondrion)
Pp3c20_21990	-2.71	2.06e-09	hypothetical protein PHYPA_022593
Pp3c4_510	-2.70	6.73e-27	NAD(P)H-quinone oxidoreductase subunit N, chloroplastic
Pp3c1_33180	-2.70	8.10e-12	protein JINGUBANG-like
Pp3c7_14650	-2.70	2.49e-14	hypothetical protein PHYPA_021858
Pp3c21_9970	-2.69	4.92e-93	long chain base biosynthesis protein 2a
Pp3c6_21520	-2.69	9.50e-45	hypothetical protein PHYPA_021944
Pp3c8_19540	-2.68	3.22e-65	B3 domain-containing protein Os11g0197600-like
Pp3c3_32420	-2.67	9.05e-37	hypothetical protein PHYPA_023159
Pp3c3_15560	-2.67	1.76e-24	uncharacterized protein LOC112284081
Pp3c17_1110	-2.66	1.38e-69	BUD13 homolog
Pp3c1_30790	-2.66	2.53e-29	hypothetical protein PHYPA_000579
Pp3c7_18910	-2.65	8.53e-06	abnormal spindle-like microcephaly-associated protein homolog isoform X1
Pp3c7_14450	-2.65	3.37e-66	retrotransposon protein, putative, Ty1-copia subclass
trnY	-2.65	3.75e-10	ribulose-1,5-bisphosphate carboxylase/oxygenase large subunit
Pp3c20_23430	-2.64	2.79e-63	triacylglycerol lipase 2-like
Pp3c19_5170	-2.63	1.87e-31	uncharacterized protein LOC112273267 isoform X1
Pp3c1_3940	-2.62	1.69e-16	predicted protein
Pp3c16_2280	-2.62	2.50e-14	hypothetical protein PHYPA_024324
Pp3c19_10230	-2.62	3.16e-45	uncharacterized protein LOC112273105
Pp3c11_9120	-2.61	5.03e-58	copalyl diphosphate synthase 1-like isoform X1
Pp3c23_1130	-2.61	9.64e-42	Retrovirus-related Pol polyprotein from transposon TNT 1-94
Pp3c10_16560	-2.61	6.18e-75	hypothetical protein PHYPA_030281
matK	-2.60	1.12e-30	ribosomal protein L22
Pp3c2_13970	-2.58	1.37e-30	U11/U12 small nuclear ribonucleoprotein 35 kDa protein
Pp3c11_4920	-2.57	3.58e-24	probable methyltransferase PMT21
Pp3c25_9350	-2.54	5.15e-53	hypothetical protein PHYPA_011202
Pp3c19_10340	-2.54	1.02e-33	hypothetical protein PHYPA_024550
Pp3c20_2620	-2.54	8.90e-42	uncharacterized protein LOC112275494
Pp3c19_14070	-2.53	2.14e-68	RING/FYVE/PHD zinc finger superfamily protein
Pp3c16_20090	-2.53	1.75e-20	hypothetical protein PHYPA_024218
Pp3c4_14700	-2.52	5.86e-45	protein TIFY 6a-like

Pp3c9_5310	-2.52	8.46e-101	hypothetical protein PHYPA_025947, partial
Pp3c1_29500	-2.51	1.77e-28	LRR receptor-like serine/threonine-protein kinase FLS2
Pp3c3_2620	-2.51	1.95e-67	ubiquitin-like-specific protease 1D
trnK	-2.50	4.85e-32	ribosomal protein L21
Pp3c20_1150	-2.49	1.65e-15	predicted protein
Pp3c19_20080	-2.49	2.31e-128	uncharacterized protein LOC112273157
Pp3c5_28500	-2.48	1.12e-62	catalase isozyme 2-like
Pp3c15_19080	-2.48	6.27e-29	cationic amino acid transporter 2, vacuolar-like
Pp3c16_19430	-2.48	5.97e-20	hypothetical protein PHYPA_024188
Pp3c9_4760	-2.48	5.34e-40	hypothetical protein PHYPA_025921
Pp3c5_640	-2.48	6.54e-35	hypothetical protein PHYPA_027224
Pp3c11_6250	-2.47	9.78e-33	glycosyltransferase-like protein
Pp3c20_20120	-2.47	5.58e-17	myosin-2 heavy chain-like
Pp3c7_21370	-2.47	6.47e-12	paired amphipathic helix protein Sin3-like 4 isoform X1
atpB	-2.47	1.09e-55	PSI P700 apoprotein A1
Pp3c2_19150	-2.47	6.73e-14	hypothetical protein PHYPA_016583
Pp3c15_22730	-2.46	1.72e-61	GDSL esterase/lipase At4g16230-like
Pp3c4_11854	-2.46	2.70e-56	HMG1/2-like protein
Pp3c5_3210	-2.46	2.33e-66	probable non-specific lipid-transfer protein AKCS9
Pp3c2_4100	-2.46	3.93e-13	putative receptor protein kinase CRINKLY4
Pp3c25_1840	-2.46	3.74e-52	hypothetical protein PHYPA_011444
Pp3c4_23200	-2.45	3.96e-08	predicted protein
Pp3c9_5060	-2.45	1.22e-77	probable xyloglucan endotransglucosylase/hydrolase protein 27
Pp3c15_22720	-2.45	3.95e-64	hypothetical protein PHYPA_003359
Pp3c8_14190	-2.44	5.77e-44	diphthine methyltransferase homolog
Pp3c12_22390	-2.44	1.82e-45	probable protein phosphatase 2C 51 isoform X1
Pp3c27_5030	-2.44	3.07e-95	protein fluG-like
Pp3c19_12910	-2.44	1.60e-53	transmembrane protein 53
Pp3c3_16280	-2.44	1.96e-28	V-type proton ATPase subunit D-like
Pp3c3_36450	-2.43	1.66e-20	hypothetical protein PHYPA_023346
Pp3c3_25110	-2.43	3.34e-63	hypothetical protein PHYPA_029660
Pp3c1_42250	-2.42	4.33e-10	60S ribosomal protein L7-2
Pp3c16_5630	-2.42	4.61e-12	AC091247_1putative polyprotein, 5'-
Pp3c4_13150	-2.41	3.30e-22	cytochrome c oxidase assembly protein COX11, mitochondrial
Pp3s775_10	-2.41	3.55e-04	hypothetical protein PHYPA_021160
Pp3c19_14710	-2.41	6.52e-38	hypothetical protein PHYPA_025308
Pp3c4_30220	-2.40	3.46e-30	bifunctional aspartokinase/homoserine dehydrogenase 1, chloroplastic-like
Pp3c2_17510	-2.40	1.05e-23	Proline-rich protein 1
Pp3c10_17170	-2.40	4.49e-11	WEB family protein At1g12150-like
Pp3c4_13140	-2.40	3.60e-22	hypothetical protein PHYPA_020859

Pp3c19_11600	-2.40	1.87e-71	hypothetical protein PHYPA_025188
Pp3c2_9980	-2.39	5.43e-29	glycerol-3-phosphate 2-O-acyltransferase 6-like
Pp3c13_4170	-2.39	1.87e-15	Retrovirus-related Pol polyprotein from transposon TNT 1-94
Pp3c12_4580	-2.39	3.26e-25	hypothetical protein PHYPA_019588
Pp3c12_7010	-2.39	3.61e-21	hypothetical protein PHYPA_019679, partial
Pp3c1_22450	-2.39	2.01e-81	---NA---
atpE	-2.38	3.71e-59	PSI P700 apoprotein A2
Pp3c19_3732	-2.38	3.56e-07	serine/threonine-protein phosphatase 4 regulatory subunit 2 isoform X2
Pp3c20_18320	-2.38	7.05e-28	hypothetical protein PHYPA_028003, partial
Pp3c3_24631	-2.37	4.78e-08	F-box protein At2g26850-like
Pp3c25_6730	-2.37	1.02e-69	pumilio homolog 1-like
Pp3c7_27000	-2.37	2.28e-61	Putative non-structural 4
Pp3c13_23660	-2.37	1.58e-16	hypothetical protein PHYPA_019393
Pp3c6_16970	-2.36	3.44e-29	ras-related protein RABH1e
Pp3c23_1972	-2.36	2.57e-13	SUN domain-containing protein 1
Pp3c4_8470	-2.36	7.29e-77	transcription factor bHLH49-like
Pp3c3_24618	-2.35	3.19e-12	golgin subfamily A member 4-like isoform X1
Pp3c15_21090	-2.35	2.20e-06	probable 1-deoxy-D-xylulose-5-phosphate synthase, chloroplastic
Pp3c3_25080	-2.35	4.13e-38	sialidase-like isoform X2
Pp3c16_11740	-2.35	5.83e-119	Transmembrane protein
Pp3c22_6380	-2.34	6.93e-83	ethylene-responsive transcription factor
Pp3c22_17930	-2.34	2.91e-36	Retrovirus-related Pol polyprotein from transposon TNT 1-94
Pp3c1_31440	-2.34	1.82e-65	Ribosomal protein L23/L15e family protein, putative
Pp3c24_20800	-2.34	1.89e-37	subtilisin-like protease SBT2.5
Pp3c21_4230	-2.33	1.68e-52	kinesin-like protein KIN-12E
Pp3c20_13600	-2.33	3.48e-18	putative lipoate-protein ligase A
Pp3c12_9500	-2.32	2.64e-67	ubiquitin carboxyl-terminal hydrolase 12
Pp3c4_3720	-2.31	1.38e-64	probable amino-acid acetyltransferase NAGS1, chloroplastic
Pp3c13_6570	-2.31	2.65e-59	protease Do-like 9
Pp3c4_22170	-2.31	1.39e-12	reverse transcriptase
Pp3c22_14530	-2.31	1.48e-80	TMV resistance protein N-like
Pp3c16_4550	-2.31	7.45e-63	hypothetical protein PHYPA_023635
Pp3c15_14090	-2.30	3.26e-10	importin subunit alpha-1-like isoform X1
Pp3c3_38220	-2.30	3.90e-12	protein SUPPRESSOR OF npr1-1, CONSTITUTIVE 1-like
Pp3c9_19520	-2.30	1.78e-57	hypothetical protein PHYPA_026402
Pp3c4_20230	-2.29	3.17e-67	disease resistance protein TAO1-like
Pp3c16_21270	-2.29	1.53e-14	fasciclin-like protein
Pp3c18_14800	-2.29	1.30e-27	peroxidase A2-like
Pp3c3_37470	-2.29	2.31e-41	protein PHR1-LIKE 2-like isoform X1

Pp3c19_20070	-2.29	8.81e-78	sulfhydryl oxidase 2-like isoform X1
Pp3c6_3820	-2.29	6.75e-07	transcription factor MYB3R-5-like isoform X3
Pp3c14_20830	-2.28	8.11e-73	mediator of RNA polymerase II transcription subunit 31-like
Pp3c23_6900	-2.28	2.12e-13	protein DOG1-like 3
Pp3c23_2010	-2.28	3.63e-24	UPF0643 protein PB2B2.08-like
Pp3c12_11700	-2.28	7.54e-58	hypothetical protein PHYPA_022665
Pp3c23_14000	-2.27	9.13e-08	biotin synthase
Pp3c3_31900	-2.27	2.69e-21	histone H3.3 isoform X1
Pp3c5_12950	-2.27	2.27e-13	probable pectinesterase 53
Pp3c25_400	-2.27	2.98e-40	protein TSS
Pp3c1_8210	-2.27	3.49e-23	UPF0051 protein in atpA 3'region-like
Pp3c19_17080	-2.26	8.24e-28	Alpha-D-phosphohexomutase superfamily
Pp3c3_29550	-2.26	6.85e-31	DNA repair endonuclease UVH1 isoform X1
Pp3c11_10950	-2.26	2.29e-45	myb family transcription factor PHL7-like
Pp3c15_12410	-2.26	1.24e-73	ras-related protein RABC1-like
Pp3c3_24600	-2.26	4.31e-13	transmembrane emp24 domain-containing protein p24beta2-like
Pp3c5_2110	-2.26	1.50e-16	hypothetical protein PHYPA_013425
Pp3c16_4520	-2.26	4.74e-57	hypothetical protein PHYPA_023633
Pp3c16_8630	-2.26	9.32e-24	hypothetical protein PHYPA_023856
Pp3c13_18780	-2.25	2.31e-24	AT-rich interactive domain-containing protein 3-like isoform X1
Pp3c25_1450	-2.25	9.26e-48	conserved oligomeric Golgi complex subunit 3
Pp3c16_3320	-2.25	1.18e-11	cytochrome c
Pp3c6_6460	-2.25	1.03e-32	iron-sulfur cluster assembly protein 1-like
Pp3c16_3930	-2.25	4.91e-48	serine/threonine-protein kinase BSK1-like isoform X1
Pp3c24_12360	-2.25	3.71e-62	hypothetical protein PHYPA_014819, partial
Pp3c3_35020	-2.24	5.79e-28	C2 domain-containing protein At1g53590-like
Pp3c5_14300	-2.24	1.36e-47	HECT-domain-containing protein
Pp3c16_10550	-2.24	2.37e-56	plastid division protein PDV2-like
Pp3c19_4310	-2.24	2.93e-25	predicted protein
Pp3c5_8580	-2.24	3.44e-16	probable sucrose-phosphatase 2 isoform X1
Pp3c4_3730	-2.24	1.71e-30	hypothetical protein PHYPA_013738
Pp3c1_18860	-2.24	7.14e-46	hypothetical protein PHYPA_031299
Pp3c11_10480	-2.24	2.27e-61	uncharacterized protein LOC112280916
Pp3c19_3840	-2.23	1.33e-100	AUGMIN subunit3
Pp3c23_2890	-2.23	2.44e-12	lysophospholipid acyltransferase LPEAT1 isoform X1
Pp3c2_24270	-2.23	2.17e-57	plant intracellular Ras-group-related LRR protein 1-like
Pp3c12_5820	-2.23	9.37e-10	hypothetical protein PHYPA_019644
Pp3c20_22420	-2.23	5.20e-69	uncharacterized protein LOC112275598

Pp3c11_15160	-2.22	3.19e-24	26S proteasome non-ATPase regulatory subunit 11 homolog
Pp3c2_28940	-2.22	2.04e-19	60S ribosomal protein L21-1
Pp3c16_8870	-2.22	7.86e-08	probable 26S proteasome subunit YTA6 isoform X1
Pp3c3_6760	-2.22	1.48e-12	RHOMBOID-like protein 13
Pp3c20_13120	-2.22	1.36e-53	hypothetical protein AXX17_ATUG04770
Pp3s116_10	-2.22	6.07e-68	hypothetical protein PHYPA_000255
Pp3c25_1440	-2.22	5.90e-63	hypothetical protein PHYPA_018282
Pp3c20_4990	-2.22	5.06e-59	hypothetical protein PHYPA_028382, partial
Pp3c6_11210	-2.22	1.27e-40	uncharacterized protein LOC112275223
Pp3c9_19800	-2.21	3.62e-38	dnaJ protein homolog
Pp3c9_20000	-2.21	4.60e-21	microtubule-associated protein 70-1-like
Pp3c11_10680	-2.21	6.00e-69	probable 3-hydroxyisobutyrate dehydrogenase-like 1, mitochondrial
Pp3c3_3230	-2.21	3.34e-07	ribosome biogenesis protein BOP1 homolog
Pp3c20_4670	-2.21	3.99e-34	Serine/threonine protein phosphatase 2A 55 kDa regulatory subunit B beta isoform
Pp3c18_9930	-2.20	7.63e-12	12-oxophytodienoate reductase 3
Pp3c19_5900	-2.20	4.31e-10	beta-amylase 2, chloroplastic-like
Pp3c24_12430	-2.20	7.53e-21	biotin carboxyl carrier protein of acetyl-CoA carboxylase
Pp3s242_10	-2.20	5.18e-56	mitochondrial dicarboxylate/tricarboxylate transporter DTC
Pp3c3_1650	-2.20	1.17e-08	TMV resistance protein N-like
Pp3c21_9980	-2.20	6.01e-98	hypothetical protein PHYPA_007864
Pp3c2_24160	-2.20	7.76e-60	uncharacterized protein LOC112289304
Pp3c14_3220	-2.19	2.81e-04	elongation factor 1-alpha-like
Pp3c5_10730	-2.19	7.90e-53	GDSL esterase/lipase At5g55050-like
Pp3c24_8780	-2.19	3.51e-35	protein translocase subunit SECA2, chloroplastic isoform X2
Pp3c11_21460	-2.19	2.26e-14	UPF0613 protein PB24D3.06c
Pp3c6_20300	-2.19	4.00e-47	WD40 repeat-like protein
Pp3c11_23670	-2.18	3.74e-52	chloroplast envelope membrane protein-like
Pp3c19_4313	-2.18	2.92e-23	dynein heavy chain 7, axonemal-like
Pp3s116_40	-2.18	7.11e-55	RGG repeats nuclear RNA binding protein A-like
Pp3c11_4830	-2.18	1.20e-14	vegetative cell wall protein gp1-like
Pp3c10_3020	-2.18	5.87e-70	hypothetical protein PHYPA_005627
Pp3c20_8260	-2.18	1.91e-48	hypothetical protein PHYPA_027651
Pp3c22_11130	-2.17	3.07e-12	Drug/metabolite transporter
Pp3c16_12600	-2.17	1.74e-44	galactan beta-1,4-galactosyltransferase GALS1-like
Pp3c13_5930	-2.17	2.39e-56	heat shock protein 83
Pp3c5_7150	-2.17	5.36e-47	KAT8 regulatory NSL complex subunit 3
Pp3c21_12100	-2.17	1.47e-92	LRR receptor-like serine/threonine-protein kinase GSO1 isoform X1
Pp3c20_1900	-2.17	5.53e-18	peroxisomal 2,4-dienoyl-CoA reductase-like

Pp3c3_29530	-2.17	2.78e-28	probable splicing factor 3A subunit 1
Pp3c7_120	-2.17	7.82e-32	protein ENHANCED DISEASE RESISTANCE 2-like
trnF	-2.17	1.56e-04	PSII 47kDa protein
Pp3c19_1490	-2.17	1.28e-30	universal stress protein PHOS34-like
Pp3c14_12070	-2.17	9.16e-60	hypothetical protein PHYPA_017420
Pp3c17_20310	-2.16	7.77e-42	ethylene-responsive transcription factor ERF084-like
Pp3c22_2660	-2.16	4.68e-27	GA20 oxidase-like protein
Pp3c26_1140	-2.16	3.56e-20	nuclear-pore anchor
Pp3c5_7180	-2.16	1.16e-46	phenylalanine ammonia-lyase-like
Pp3c22_7690	-2.16	6.71e-88	predicted protein
Pp3c3_34790	-2.16	4.39e-51	hypothetical protein PHYPA_023263
Pp3c5_7110	-2.16	4.27e-47	uncharacterized protein LOC112274862 isoform X2
Pp3c12_20650	-2.15	2.35e-62	condensin complex subunit 2-like
Pp3c22_10850	-2.15	1.36e-51	disease resistance-like protein CSA1
Pp3c6_26200	-2.15	4.46e-35	histone H2B.8-like
Pp3c17_14680	-2.15	7.98e-50	inactive protein kinase SELMODRAFT_444075-like
Pp3c26_630	-2.15	2.18e-43	predicted protein
Pp3c1_7380	-2.15	8.56e-88	probable protein phosphatase 2C 15 isoform X1
Pp3c6_200	-2.15	2.02e-12	Retrovirus-related Pol polyprotein from transposon TNT 1-94
Pp3c17_14681	-2.15	6.89e-50	hypothetical protein PHYPA_004393
Pp3c11_2650	-2.15	1.38e-33	hypothetical protein PHYPA_009679
Pp3c24_13190	-2.14	4.89e-15	60S ribosomal protein L28-1-like
Pp3c11_12080	-2.14	9.75e-70	eukaryotic translation initiation factor 3 subunit C-like
Pp3c6_6830	-2.14	1.15e-57	F-box protein At1g47056-like
Pp3c20_15220	-2.14	1.27e-13	---NA---
Pp3c18_13090	-2.14	1.20e-46	uncharacterized protein LOC112279900
Pp3s1006_30	-2.14	2.30e-04	uncharacterized protein LOC112290578 isoform X3
Pp3c12_15820	-2.13	3.63e-30	FT-interacting protein 1-like
Pp3c1_32200	-2.13	2.40e-50	germin-like protein 1-3
Pp3c10_15730	-2.13	1.50e-86	polyadenylate-binding protein-interacting protein 7-like
Pp3c5_19300	-2.13	2.96e-25	hypothetical protein PHYPA_013332
Pp3c1_27990	-2.13	1.45e-73	uncharacterized protein LOC112285443 isoform X1
Pp3c25_15260	-2.12	1.67e-50	CAX-interacting protein 4-like
Pp3c16_15270	-2.12	1.57e-79	DEAD-box ATP-dependent RNA helicase 42
Pp3c7_23980	-2.12	2.66e-84	DNA-(apurinic or apyrimidinic site) lyase
Pp3c21_6200	-2.12	4.59e-19	Glycosyl hydrolase, five-bladed beta-propellor domain-containing protein

Pp3c13_20640	-2.12	1.79e-30	Prestalk-specific protein tagB
Pp3c10_4440	-2.12	2.56e-27	probable protein phosphatase 2C 60 isoform X1
Pp3c18_8820	-2.12	4.66e-61	Retrovirus-related Pol polyprotein from transposon TNT 1-94
Pp3c15_16700	-2.12	9.37e-46	ribosomal protein L16 (mitochondrion)
Pp3c23_1980	-2.12	5.75e-15	sodium-dependent phosphate transport protein 1, chloroplastic
Pp3c2_4940	-2.12	1.08e-18	uncharacterized endoplasmic reticulum membrane protein YGL010W-like
Pp3c4_28370	-2.12	1.79e-69	vasohibin-2 isoform X3
Pp3c13_15240	-2.12	4.59e-13	hypothetical protein PHYPA_020063
Pp3c3_35180	-2.11	1.21e-70	lanC-like protein GCL1
Pp3c9_19950	-2.11	1.01e-31	putative clathrin assembly protein At5g35200
Pp3c4_31680	-2.11	1.02e-06	RNA helicase nonsense mRNA reducing factor
Pp3c13_2310	-2.11	7.83e-43	SAL1 phosphatase-like
Pp3c2_22630	-2.11	1.37e-33	hypothetical protein PHYPA_015741
Pp3c1_1330	-2.11	4.93e-64	hypothetical protein PHYPA_018926
Pp3c7_24650	-2.11	9.44e-38	hypothetical protein PHYPA_029322, partial
Pp3c20_5760	-2.11	2.29e-51	---NA---
Pp3c7_11090	-2.10	5.50e-57	aspartyl protease family protein 2-like
Pp3c24_18690	-2.10	4.38e-40	autophagy-related protein 8C
Pp3c14_11030	-2.10	6.64e-70	calnexin homolog
Pp3c8_5370	-2.10	9.00e-33	F-box only protein 6-like
Pp3c4_8171	-2.10	6.22e-64	hydroxyproline O-arabinosyltransferase 3-like
Pp3c6_18710	-2.10	1.81e-39	proteasome subunit alpha type-7
Pp3c12_22330	-2.10	8.35e-06	protein ECERIFERUM 3
Pp3c26_6740	-2.10	3.75e-36	serine carboxypeptidase-like
Pp3c12_9890	-2.10	3.14e-49	serine/threonine-protein kinase HT1-like
Pp3c7_11100	-2.10	5.63e-56	trihelix transcription factor ASR3-like
Pp3c18_13350	-2.10	2.53e-23	---NA---
Pp3c10_9740	-2.09	6.07e-68	casein kinase 1-like protein 1
Pp3c7_22220	-2.09	1.12e-07	pol-like protein
Pp3c1_4010	-2.09	8.23e-53	U-box domain-containing protein 43-like
Pp3c27_6070	-2.09	1.27e-17	hypothetical protein PHYPA_001573, partial
Pp3c7_12850	-2.09	1.50e-42	hypothetical protein PHYPA_021789
Pp3c19_12750	-2.09	4.88e-39	hypothetical protein PHYPA_025225
Pp3c14_7930	-2.08	7.74e-25	aldehyde oxidase GLOX-like
Pp3c17_4440	-2.08	1.21e-10	arginyl-tRNA--protein transferase 2-like
Pp3c12_5220	-2.08	7.77e-15	enoyl-[acyl-carrier-protein] reductase [NADH] 1, chloroplastic-like
Pp3c24_20960	-2.08	1.44e-41	protein DETOXIFICATION 16-like
Pp3c8_1940	-2.08	4.02e-55	protein GLE1
Pp3c20_2980	-2.08	9.54e-47	protein NLP1
Pp3c4_8330	-2.08	5.06e-57	receptor kinase-like protein

Pp3c10_13720	-2.08	9.67e-04	ureide permease 1-like
Pp3c26_12570	-2.08	4.42e-68	hypothetical protein PHYPA_011344
Pp3c18_8240	-2.08	1.23e-57	hypothetical protein PHYPA_025065
Pp3c13_14980	-2.08	3.95e-44	---NA---
Pp3c1_30428	-2.07	3.66e-16	ATP-dependent RNA helicase DBP2-like
Pp3c17_3830	-2.07	2.29e-49	transcription factor TCP15-like
Pp3c12_8680	-2.07	2.94e-13	transmembrane protein 120 homolog
Pp3c16_3080	-2.07	5.24e-10	zinc finger CCCH domain-containing protein 35-like
Pp3c2_17110	-2.07	1.09e-51	hypothetical protein PHYPA_016472
Pp3c1_14760	-2.07	4.18e-23	hypothetical protein PHYPA_017974
Pp3c27_440	-2.06	8.19e-13	deoxyhypusine synthase isoform X1
Pp3c15_10950	-2.06	3.96e-21	mitogen-activated protein kinase kinase kinase YODA-like
Pp3c13_1510	-2.06	4.84e-06	Retrovirus-related Pol polyprotein from transposon TNT 1-94
Pp3c16_2080	-2.06	9.73e-55	Retrovirus-related Pol polyprotein from transposon TNT 1-94
Pp3c7_6700	-2.06	4.42e-05	transcription factor GAMYB-like
Pp3c15_12810	-2.06	2.71e-47	tubulin alpha-3 chain-like
Pp3c2_29140	-2.06	3.53e-23	ubiquitin-conjugating enzyme E2 2
Pp3c11_15790	-2.06	9.85e-49	V-type proton ATPase subunit a3-like isoform X1
Pp3c12_4540	-2.06	2.47e-58	hypothetical protein PHYPA_019585
Pp3c9_5650	-2.05	1.08e-06	60S ribosomal protein L37-3
Pp3c1_19190	-2.05	7.97e-64	ammonium transporter 1 member 3-like
Pp3c24_10800	-2.05	1.46e-28	Embryogenesis-associated protein EMB8
Pp3c16_23250	-2.05	1.27e-28	GATA zinc finger protein
Pp3c7_14840	-2.05	5.01e-21	putative dihydroflavonol-4-reductase
Pp3c5_2700	-2.05	4.62e-37	putative nuclease HARBI1
Pp3c7_20320	-2.05	5.89e-43	transcription elongation factor B polypeptide 3
Pp3c23_22230	-2.05	2.48e-39	hypothetical protein PHYPA_014494
Pp3c23_6070	-2.05	1.52e-57	hypothetical protein PHYPA_015644
Pp3c24_5330	-2.05	4.81e-07	hypothetical protein PHYPA_018827
Pp3c1_18350	-2.05	1.10e-17	ATP-dependent DNA helicase SRS2-like protein At4g25120
Pp3c24_19080	-2.04	7.67e-29	calcium sensing receptor, chloroplastic-like
Pp3c7_23810	-2.04	1.18e-61	calmodulin-binding ion transporter-like protein
Pp3c1_13800	-2.04	2.10e-07	G-type lectin S-receptor-like serine/threonine-protein kinase SD2-5
Pp3c24_17170	-2.04	1.36e-34	nuclear pore complex protein NUP54
Pp3c3_34180	-2.04	9.15e-52	protein CONSERVED IN THE GREEN LINEAGE AND DIATOMS 27, chloroplastic-like
Pp3c24_12180	-2.04	1.34e-50	hypothetical protein PHYPA_014424
Pp3c2_12860	-2.04	4.31e-08	hypothetical protein PHYPA_016259

Pp3c6_28830	-2.03	1.53e-75	dihydroceramide fatty acyl 2-hydroxylase FAH1-like
Pp3c9_9200	-2.03	1.75e-40	electron transfer flavoprotein-ubiquinone oxidoreductase, mitochondrial-like isoform X1
Pp3c9_20020	-2.03	1.62e-25	microtubule-associated protein 70-1-like
Pp3c21_11130	-2.03	2.79e-42	phospholipase D alpha 1
Pp3c9_15440	-2.03	7.52e-47	polyphenol oxidase, chloroplastic-like
Pp3c1_4350	-2.03	8.01e-35	hypothetical protein PHYPA_001949, partial
Pp3c4_20310	-2.03	1.57e-09	hypothetical protein PHYPA_025225
Pp3c10_22780	-2.03	3.72e-35	---NA---
Pp3c1_3040	-2.02	3.08e-17	ACT domain-containing protein ACR3-like isoform X4
Pp3c6_10350	-2.02	3.53e-12	chlorophyllide a oxygenase, chloroplastic
Pp3c26_11090	-2.02	3.32e-40	cytosolic purine 5'-nucleotidase-like isoform X1
Pp3c11_4100	-2.02	2.47e-29	ENHANCER OF AG-4 protein 2
Pp3c24_19680	-2.02	5.21e-13	J domain-containing protein spf31
Pp3c18_7850	-2.02	2.26e-42	myosin heavy chain kinase A-like isoform X3
Pp3c25_4520	-2.02	9.12e-21	protein MANNAN SYNTHESIS-RELATED 1-like isoform X2
Pp3c13_24600	-2.02	2.91e-31	stress-response A/B barrel domain-containing protein UP3-like
Pp3c12_25900	-2.02	6.36e-37	hypothetical protein PHYPA_010127
Pp3c13_16600	-2.02	9.42e-07	hypothetical protein PHYPA_020126
Pp3c12_21540	-2.02	1.22e-55	uncharacterized protein LOC112284578
Pp3c14_25660	-2.02	1.31e-13	uncharacterized protein LOC112290419
Pp3c4_22100	-2.01	1.06e-51	calcineurin B-like protein 3
Pp3c8_17920	-2.01	5.43e-28	DNA-directed RNA polymerase II subunit 4
Pp3c4_8159	-2.01	3.18e-53	leucine-rich repeat receptor-like serine/threonine/tyrosine-protein kinase SOBIR1
Pp3c15_16860	-2.01	8.11e-06	oxygen-evolving enhancer protein 2, chloroplastic-like
Pp3c25_7030	-2.01	1.56e-36	probable magnesium transporter NIPA8
Pp3c2_4740	-2.01	1.10e-56	protein S-acyltransferase 21
Pp3c5_3190	-2.01	9.03e-08	Thioredoxin superfamily protein isoform 1
Pp3c3_26700	-2.01	7.65e-14	vicilin-like seed storage protein At2g18540 isoform X2
Pp3c15_11070	-2.01	2.57e-45	hypothetical protein PHYPA_002883
Pp3c6_10610	-2.01	1.04e-31	hypothetical protein PHYPA_007844
Pp3c2_13420	-2.01	1.63e-24	hypothetical protein PHYPA_016289
Pp3c6_27020	-2.01	1.31e-04	hypothetical protein PHYPA_021549
Pp3c19_4880	-2.01	1.56e-27	hypothetical protein PHYPA_024468
Pp3c6_21060	-2.00	7.07e-48	diphthamide biosynthesis protein 3-like
Pp3c17_23380	-2.00	1.63e-37	mitochondrial inner membrane protease subunit 2-like isoform X1

Pp3c3_1020	-2.00	3.03e-30	putative DD1A protein
Pp3c12_6700	-2.00	3.54e-47	serine/threonine-protein kinase 16-like
Pp3c16_5790	-2.00	3.28e-39	squalene monooxygenase-like
Pp3c11_800	-2.00	2.17e-30	hypothetical protein PHYPA_010493
Pp3c16_16040	-2.00	2.59e-70	hypothetical protein PHYPA_024003
Pp3c15_14300	-6.04	1.21e-134	scarecrow-like protein 28
Pp3c11_6580	-4.58	9.26e-139	hypothetical protein PHYPA_009755
Pp3c8_21760	-4.54	5.89e-39	6-phosphogluconate dehydrogenase, decarboxylating 3
Pp3c10_11330	-4.14	9.52e-76	phenylalanine ammonia-lyase-like
Pp3c5_9700	-4.02	9.11e-29	probable pectate lyase 5
Pp3c18_14350	-3.91	1.52e-63	hypothetical protein PHYPA_005345
Pp3c16_19790	-3.81	5.63e-18	metal tolerance protein C2
Pp3c17_5760	-3.68	5.79e-47	glycerophosphodiester phosphodiesterase GDPD1, chloroplastic
Pp3s350_10	-3.52	9.98e-12	high chlorophyll fluorescence phenotype 173
Pp3c13_15640	-3.41	8.28e-21	endoplasmic reticulum oxidoreductin-1
Pp3c6_10090	-3.40	1.25e-83	probable glycosyltransferase At5g03795
Pp3c15_22920	-3.39	6.59e-60	DNA polymerase delta catalytic subunit-like isoform X5
Pp3c13_13170	-3.35	5.72e-152	hypothetical protein PHYPA_019951
Pp3c13_16130	-3.32	7.40e-89	Mitochondrial import inner membrane translocase subunit tim-10 isoform 1
Pp3c13_15980	-3.32	3.57e-91	hypothetical protein PHYPA_019196
trnG	-3.28	3.32e-17	PSII L-protein
Pp3c24_13310	-3.24	6.58e-15	hypothetical protein PHYPA_018322

Supporting Information Table S3: Data of upregulated and downregulated genes represented in Figure 4.

Biological process	Sequence ID	Gene name	Description	LogFC	FDR
Phenyl-propanoids pathway	Pp3c21_7680	*pal*	Phenylalanine ammonia-lyase	8.40	1.50e-23
	Pp3c2_32410	*pal*	Phenylalanine ammonia-lyase	8.19	4.58e-41
	Pp3c21_7670	*pal*	Phenylalanine ammonia-lyase	8.18	1.18e-20
	Pp3c19_13690	*pal*	Phenylalanine ammonia-lyase	7.04	5.45e-35
	Pp3c13_9000	*pal*	Phenylalanine ammonia-lyase	6.73	3.00e-226
	Pp3c2_30610	*pal*	Phenylalanine ammonia-lyase	6.67	7.51e-157
	Pp3c1_18830	*pal*	Phenylalanine ammonia-lyase	6.37	3.04e-185
	Pp3c1_18940	*pal*	Phenylalanine ammonia-lyase	6.33	3.22e-176
	Pp3c26_2420	*pal*	Phenylalanine ammonia-lyase	4.83	3.04e-91
	Pp3c19_2670	*pal*	Phenylalanine ammonia-lyase	2.18	2.96e-05
	Pp3c5_7180	*pal*	Phenylalanine ammonia-lyase	-2.16	1.16e-46
	Pp3c10_11330	*pal*	Phenylalanine ammonia-lyase	-4.14	9.52e-76
	Pp3c2_27270	*chi*	Chalcone-flavonone isomerase	2.99	1.53e-84
	Pp3c16_23390	*cad*	Cinnamyl alcohol dehydrogenase	2.63	1.79e-25
	Pp3c19_2720	*cad*	Cinnamyl alcohol dehydrogenase 6	2.44	2.08e-09
	Pp3c16_17510	*chs*	Chalcone synthase	2.43	1.23e-26
	Pp3c3_27940	*4cl*	4-Coumarate-CoA ligase-5	2.00	1.29e-16
	Pp3c14_24040	*pel*	Pectate lyase-like	6.66	5.89E-79

Cell wall compounds biosynthesis	Pp3c13_17633	*csla*	beta-Mannosyltransferase 1	5.27	8.98e-14
	Pp3c4_20210	*tbl*	Protein trichome birefringence	4.89	2.9e-13
	Pp3c1_28020	*pme*	Pectinesterase 53	4.46	1.26e-32
	Pp3c3_31700	*pagr*	Protein pectic arabinogalactan synthesis-related	4.00	3.54e-17
	Pp3c11_9619	*lrx*	Leucine-rich repeat extensin-like protein 5	2.69	3.55e-22
	Pp3c20_200	*cals*	Callose synthase 12	2.38	1.15e-27
	Pp3c5_14090	*cals*	Callose synthase 9	2.28	4.2e-8
	Pp3c27_8130	*csld*	Cellulose synthase	2.37	1.9e-67
	Pp3c6_6545	*csld*	Cellulose synthase	2.11	5.57e-34
	Pp3c9_19210	*gals*	Galactan beta-1,4-galactosyltransferase	2.13	6e-25
	Pp3c22_16800	*gp*	Vegetative cell wall protein gp1	2.02	3.03e-33
Defense response	Pp3c2_36350	*erg*	Elicitor-responsive protein 3	6.56	7.85e-61
	Pp3c21_14500	*tpr*	Topless-related protein 1-like	6.07	1.93e-203
	Pp3c19_6710	*tao*	Disease resistance protein TAO1	4.15	1.24e-61
	Pp3c9_14080	*tao*	Disease resistance protein TAO1	2.83	5.17e-26
	Pp3c6_7260	*tao*	Disease resistance protein TAO1	2.11	1.32e-45
	Pp3c11_12290	*snc1*	Protein suppressor of NPR1-1, constitutive 1	3.25	5.24e-11
	Pp3c22_20810	*snc1*	Protein suppressor of NPR1-1, constitutive 1	2.89	2.01e-14
	Pp3c23_17280	*snc1*	Protein suppressor of NPR1-1, constitutive 1	2.22	5.63e-6

	Pp3c5_13990	*snc1*	Protein suppressor of NPR1-1, constitutive 1	2.19	6.26e-70
	Pp3c10_20210	*nramp*	Nramp-domain-containing protein	2.64	5.73e-47
	Pp3c13_4250	*tmv_rpn*	TMV resistance protein N-like	2.28	2.8e-17
	Pp3c7_23740	*bbd*	bifunctional nuclease 1-like	2.21	6.06e-26
	Pp3c1_10860	*tmv-rpn*	TMV resistance protein N-like	2.03	4.06e-35
	Pp3c2_2790	*mlp*	MLP-like protein 423	2.26	1.25e-08
	Pp3c3_6500	*mlo*	MLO-like protein 5	2.16	1.19e-67
	Pp3c12_20050	*mlo*	MLO-like protein 14	2.04	6.42e-18
	Pp3c13_9000	*dir*	Putative lipid-transfer protein DIR1	2.05	1.41e-5
	Pp3c7_120	*edr*	Protein Enhanced Disease Resistance 2	-2.17	7.82e-32
LRR-receptors	Pp3c5_23340	*traf*	TNF receptor-associated factor homolog 1a	6.97	7.32e-50
	Pp3c6_10250	*bak*	LRR receptor kinase BAK1	3.04	2.2e-23
	Pp3c2_30610	*erl*	LRR receptor-like ser/thr-protein kinase ERL1	2.14	1.46e-3
	Pp3c7_22440	*lrr-pk*	Probable leucine-rich repeat receptor-like protein kinase At1g68400	6.22	6.62e-61
	Pp3c21_12100	*gso*	LRR receptor-like ser/thr-protein kinase GSO1	-2.17	1.47e-92
ROS	Pp3c17_9830	*sod*	Superoxide dismutase [Cu-Zn]-like	4.05	1.08e-117
	Pp3c9_2080	*prx*	Peroxidase N1-like	3.18	1.15e-13

	Pp3c6_26980	*glox*	Aldehyde oxidase GLOX-like	2.65	3.07e-09
	Pp3c10_13010	*glox*	Aldehyde oxidase GLOX-like	2.36	3.09e-08
	Pp3c18_14800	*prx*	Peroxidase A2-like	-2.29	1.3e-27
	Pp3c3_10731	*prx*	Thioredoxin peroxidase 1	-2.93	1.34e-12
	Pp3c5_28500	*cat*	Catalase isozyme 2	-2.48	1.12e-62
Photosynthesis / Chloroplast	Pp3s350_10	*hcf*	High chlorophyll fluorescence phenotype 173	-3.52	9.98e-12
	Pp3c13_16030	*rbc*	Ribulose Bisphosphate Carboxylase small chain clone 512-like	-2.96	3.7e-14
	trnY	*rbc*	Ribulose-1,5-Bisphosphate Carboxylase/Oxygenase large subunit	-2.65	3.75e-10
	trnG	*psb*	Photosystem II L-protein	-3.28	3.32e-17
	trnE	*psb*	Photosystem II protein	-3.00	2.85e-3
	trnV	*psa*	Photosystem I-protein	-2.81	4.11e-09
	atpB	*psa*	Photosystem P700 apoprotein A1	-2.47	1.09e-55
	atpE	*psa*	Photosystem P700 apoprotein A2	-2.38	3.71e-59
	trnF	*psb*	Photosystem II 47kDa protein	-2.17	1.56e-4
	Pp3c4_510	*ndhN*	NAD(P)H-quinone oxidoreductase subunit N, chloroplastic	-2.70	6.73e-27
	Pp3c11_23670	*cemA*	Chloroplast envelope membrane protein	-2.18	3.74e-52
	Pp3c6_10350	*cao*	Chlorophyllide a oxygenase, chloroplastic	-2.02	3.53e-12

Transcription factor	Pp3c2_32400	*erf*	Ethylene-Responsive Transcription Factor RAP 2.11	6.39	8.19e-60
	Pp3c12_11940	*bzip*	bZIP Transcription factor 60	5.28	3.7e-60
	Pp3c24_19980	*tcp*	Transcription factor TCP15	4.93	3.01e-10
	Pp3c17_3830	*tcp*	Transcription factor TCP15	-2.07	2.29e-49
	Pp3c8_16240	*bhlh*	Transcription factor bHLH66	4.73	2.3e-59
	Pp3c3_23910	*myb*	Transcription factor MYB21	4.38	8.4e-40
	Pp3c6_2730	*hec*	Transcription factor HEC2	3.88	4.53e-26
	Pp3c17_3860	*marR*	Transcriptional regulator, MarR family	3.31	8.33e-33
	Pp3c3_32210	*eif*	Eukaryotic translation initiation factor 3 subunit K	3.79	1.83e-47
	Pp3c18_2960	*eif*	Eukaryotic translation initiation factor 3 subunit C	3.18	2.01e-4
	Pp3c10_3550	*tfIID*	Transcription initiation factor TFIID subunit 14b	3.17	4.36e-57
	Pp3c10_16800	*ap*	AP-1 complex subunit gamma-2	3.15	1.21e-22
	Pp3c12_25330	*posF*	Probable transcription factor PosF21	3.11	9.28e-24
	Pp3c15_3560	*eif*	Eukaryotic translation initiation factor 4E-1	2.96	1.94e-90
	Pp3c9_16970	*erf*	Ethylene-Responsive Transcription Factor ERF039	2.94	7.59e-45
	Pp3c22_9050	*asr3*	Trihelix transcription factor ASR3	2.91	1.39e-19
	Pp3c15_20210	*bzip*	Basic leucine zipper 61	2.81	8.32e-13

	Pp3c6_25090	*mads*	Putative MADS-domain transcription factor	2.76	4.2e-15
	Pp3c23_2252	*mads*	Putative MADS-domain transcription factor	2.18	8.04e-09
	Pp3c3_15040	*ilr*	Transcription factor ILR3	2.51	1.53e-09
	Pp3c2_18850	*hox*	Homeobox-leucine zipper protein HOX20	2.51	1.46e-22
	Pp3c17_13270	*nf-yb*	Nuclear transcription factor Y subunit B-3	2.43	8.99e-34
	Pp3c4_29120	*fama*	Transcription factor FAMA	2.31	6.23e-42
	Pp3c15_20060	*dof*	DOF zinc finger protein DOF4.3	2.25	5.96e-08
	Pp3c21_1310	*erf*	Ethylene-Responsive Transcription Factor 3	2.23	1.84e-08
	Pp3c5_24940	*nac*	NAC domain-containing protein 76	2.13	7.26e-41
	Pp3c7_17000	*lug*	Transcriptional corepressor LEUNIG	2.04	4.34e-5
	Pp3c2_28350	*ap*	AP-1 complex subunit gamma-2	2.01	2.66e-5
	Pp3c7_20320	*elo*	Transcription elongation factor B polypeptide 3	-2.05	5.89e-43
	Pp3c7_6700	*gamyb*	Transcription factor GAMYB	-2.06	4.42e-5
	Pp3c11_12080	*eif*	Eukaryotic translation initiation factor 3 subunit C	-2.14	9.75e-70
	Pp3c17_20310	*erf*	Ethylene-Responsive Transcription Factor ERF084	-2.16	7.77e-42
	Pp3c11_10950	*myb*	MYB family transcription factor PHL7	-2.26	2.29e-45

	Pp3c6_3820	*myb*	Transcription factor MYB3R-5	-2.29	6.75e-7
	Pp3c4_8470	*bhlh*	Transcription factor bHLH49	-2.36	7.29e-77
	Pp3c4_1450	*erf*	Ethylene-responsive transcription factor ERF043	-2.71	1.98e-20
	Pp3c13_10520	*egl*	Transcription factor EGL1	-2.87	2.63e-17
	Pp3c20_2980	*nlp*	Protein NLP1	-2.08	9.54e-47
	Pp3c19_3900	*nlp*	Protein NLP1	-3.21	4.17e-45
Ca2+ signaling	Pp3c27_3620	*crck*	Calmodulin-binding receptor-like cytoplasmic kinase 2	4.39	2.89e-65
	Pp3c2_17270	*cpk*	Calcium-dependent protein kinase 26-like	4.02	8.93e-28
	Pp3c10_5660	*calt*	Caltractin	3.74	1.26e-62
	Pp3c2_27000	*slo*	Calcium-activated potassium channel slowpoke-like	3.18	5.75e-27
	Pp3c4_27280	*tpc*	Two pore calcium channel protein 1	2.93	2.15e-16
	Pp3c16_23100	*cpk*	Calcium-dependent protein kinase	2.55	6.15e-3
	Pp3c26_12710	*aca*	Calcium-transporting ATPase 8, plasma membrane-type	2.39	6.02e-13
	Pp3c4_22100	*cbl*	Calcineurin B-like protein 3	-2.01	1.06e-51
	Pp3c24_19080	*cas*	Calcium sensing receptor, chloroplastic	-2.04	7.67e-29
